# Microglia Rank signaling regulates GnRH neuronal function and the Hypothalamic-Pituitary-Gonadal axis

**DOI:** 10.1126/science.aeb6999

**Published:** 2026-05-21

**Authors:** Alejandro Collado-Sole, Nozha Borjini, Jing Zhai, Francisco Ruiz-Pino, Gonzalo Soria-Alcaide, Cintia Folgueira, Celia García-Vilela, Beatriz Romero-de la Rosa, Victor Lopez, Yassine Zouaghi, An Jacobs, Bella Mora-Romero, Alexandra Barranco, Guillermo Yoldi, Karine Rizzoti, Guadalupe Sabio, Gema Perez-Chacon, Patricia G. Santamaria, Jose Antonio Esteban, Nathalie Journiac, Vincent Prevot, Alberto Pascual, Rafael Fernández-Chacón, Manuel Tena-Sempere, Nelly Pitteloud, Eva Gonzalez-Suarez

**Affiliations:** 1 Tumor Biology and Immunology Program, Spanish National Cancer Research Centre (CNIO), 28029 Madrid, Spain; 2 Instituto de Biomedicina de Sevilla (IBiS), Hospital Universitario Virgen del Rocío/CSIC/ Universidad de Sevilla, 41013 Seville, Spain; 3 Department of Medical Physiology and Biophysics, School of Medicine, University of Seville, 41009 Seville, Spain; 4 Centro de Investigación Biomédica en Red sobre Enfermedades Neurodegenerativas (CIBERNED), 28029 Madrid, Spain; 5 Service of Endocrinology, Diabetology, and Metabolism, Lausanne University Hospital; Lausanne 1011, Switzerland; 6 Faculty of Biology and Medicine, University of Lausanne; Lausanne 1005, Switzerland; 7 Instituto Maimónides de Investigación Biomédica de Córdoba/ Hospital Universitario Reina Sofia; 14004 Córdoba, Spain; 8 Department of Cell Biology, Physiology and Immunology, University of Córdoba; 14071 Córdoba, Spain; 9 CIBER Fisiopatología de la Obesidad y Nutrición, Instituto de Salud Carlos III; 14004 Córdoba, Spain; 10 Centro Nacional de Investigaciones Cardiovasculares (CNIC), 28029 Madrid, Spain; 11 Grupo Fisiopatología Endocrina, Departamento de Endocrinología, Instituto de Investigación Sanitaria de Santiago de Compostela, Complexo Hospitalario Universitario de Santiago (CHUS/SERGAS), 15706 Santiago de Compostela, Spain; 12 Department of Molecular Neuropathology, Centro de Biología Molecular Severo Ochoa ( CSIC-UAM), 28049 Madrid, Spain; 13 Department of Pediatrics, Stanford University, Stanford CA 94304, California, United States; 14 Department of Medicine, Endocrinology, Diabetology, and Metabolism Unit, CHUV; Lausanne 1005, Switzerland; 15 Departamento de Biología Celular, Facultad de Biología, Universidad de Sevilla, 41009 Seville, Spain; 16 Oncobell, Bellvitge Biomedical Research Institute, IDIBELL; 08908 Barcelona, Spain; 17 Laboratory of Stem Cell Biology and Developmental Genetics, The Francis Crick Institute; NW1 1AT London, United Kingdom; 18 Centre for Endocrinology, William Harvey Research Institute, Faculty of Medicine and Dentistry, Queen Mary University of London, E1 4NS London, UK; 19 Tumor Microenvironment and Immunomodulation Unit, Instituto de Salud Carlos III (ISCIII), 28222 Madrid, Spain; 20 Lille University, Inserm, CHU Lille, Lille Neuroscience and Cognition, UMR-S 1172, Labex DistAlz, 59000 Lille, France; 21 Laboratory of Development and Plasticity of the Neuroendocrine Brain, FHU 1000 Days for Health, EGID, 59045 Lille, France

## Abstract

The hypothalamic-pituitary-gonadal axis (HPG) controls pubertal development, sexual maturation, and fertility. We identified a role of hypothalamic microglia in controlling the HPG axis through Rank signaling. Whole-body and microglia Rank depletion led to hypogonadotropic hypogonadism (HH) resulting from an alteration in gonadotropin-releasing hormone (GnRH) neuron function. In addition, we identified rare gene variants of *RANK* in patients with HH. Transcriptional profiling upon Rank loss revealed defective microglia activation and morphological alterations in the median eminence, decreasing the contacts and engulfment of GnRH terminal projections and impairing GnRH neuronal responses to kisspeptin. Overall, our data uncover the microglia as regulator of GnRH neuronal function through Rank signaling, with potential implications for reproductive maturation and fertility.

Reproductive hormones are closely related to the receptor activator of nuclear factor-kappa B (RANK) signaling pathway, regulating its activation in the bone and in the mammary gland. In the mammary epithelium, RANK ligand (RANKL) is the main paracrine mediator of progesterone, controlling mammary epithelial expansion, alveologenesis and lactation (1–4). In the bone, the drop in estrogen in menopause triggers overactivation of RANK signaling in osteoclasts, causing exacerbated bone turnover and osteoporosis (5). Reproductive hormones regulate puberty onset, which is dictated by the activation of the hypothalamic-pituitary-gonadal (HPG) axis (6). Specifically, gonadotropin-releasing hormone (GnRH) neurons are the main effectors for the control of the HPG axis in the brain, being localized in the preoptic area (PoA) of the hypothalamus, and constitute a neuronal network with projections to the median eminence (ME) (7). Their pulsatile secretion is mainly dictated by the kisspeptin system (8) and determines the patterns of secretion of gonadotropins, follicle-stimulating hormone (FSH) and luteinizing hormone (LH), thereby controlling puberty onset, gonadal development and fertility.

Here, we provide mechanistic evidence indicating that microglia hypothalamic Rank signaling is involved in the activation of the HPG axis, with a primary action on the regulation of GnRH neuron function. Through GnRH neurons, microglia Rank signaling regulates gonadotropins, the production of sex hormones and, consequently, gonadal development and reproduction in both sexes. Inducible Rank deletion in puberty and adulthood disrupts the HPG axis in males. In parallel, we have identified different *RANK* gene variants in patients with congenital hypogonadotropic hypogonadism (CHH), a human syndrome that shares several phenotypic characteristics with full-body and microglia-specific Rank-deficient mouse models, reinforcing the importance of Rank signaling within microglia in HPG regulation, pubertal onset, and fertility.

## Rank loss leads to severe hypogonadotropic hypogonadism in mice

**Fig. 1:**
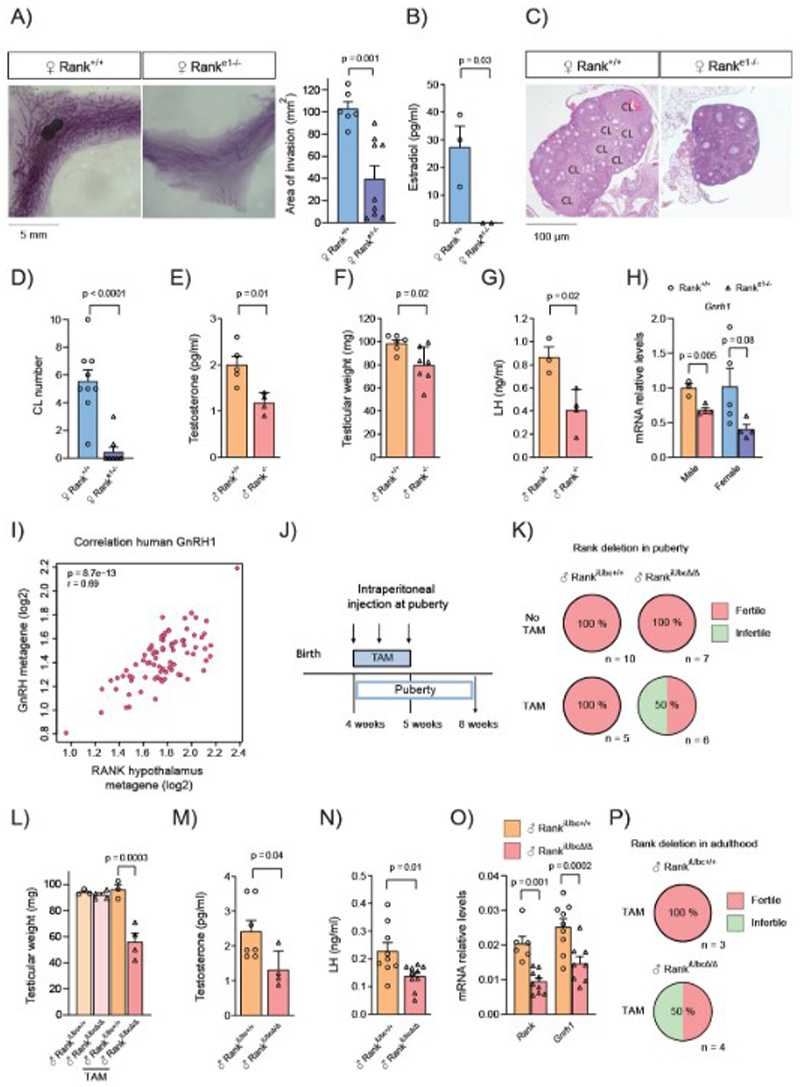
Whole-body Rank deletion induces HH in mice. (A) Representative whole mounts and quantification of epithelial invasion in mammary glands from Rank^+/+^ and Rank^e1-/-^ mice. (B) Estradiol (E2) concentration in serum from Rank^+/+^ and Rank^e1-/-^ females. (C) Representative images of H&E staining of Rank^+/+^ and Rank^e1-/-^ ovaries. (D) Quantification of corpora lutea (CL) in Rank^+/+^ and Rank^e1-/-^ ovaries from panel c. (E) Testosterone concentration in serum from Rank^+/+^ and Rank^-/-^ mice. (F) Testicular weight in Rank^+/+^ and Rank^-/-^ mice. (G) LH concentration in serum from Rank^+/+^ and Rank^-/-^ males. (H) *Gnrh1* gene expression in the hypothalamus from Rank^ex1-/-^ male and female mice relative to control littermates. (I) Correlation between RANK metagene and GnRH metagenes analyzed in the human hypothalamus (GTEx) using GEPIA2 (Gene Expression Profiling Interactive Analysis version 2). The correlation index was calculated using Pearson correlation. (J) TAM treatment protocol to induce Rank deletion at the onset of puberty. Rank^iUbcΔ/Δ^ males and their Rank^iUbc+/+^ littermates were treated at 4 weeks of age with 3 doses of TAM (100 mg/kg) every other day for one week and euthanized 4 weeks after the first treatment. (K) Percentage of fertile Rank^iUbcΔ/Δ^ / Rank^iUbc+/+^ male mice upon Rank deletion at puberty, including TAM control and experimental treated (as shown in J) and untreated male mice. (L) Testicular weight of Rank^iUbc+/+^ and Rank^iUbcΔ/Δ^ males upon Rank deletion at puberty, including TAM control and experimental treated mice (M) Testosterone concentration in serum from Rank^iUbc+/+^ and Rank^iUbcΔ/Δ^ males upon Rank deletion at puberty. (N) LH concentration in blood from Rank^iUbcΔ/Δ^ males and control littermates upon Rank deletion in puberty. (O) *Gnrh1* and *Rank* mRNA amounts in the hypothalamus from Rank^iUbcΔ/Δ^ males and control littermates upon Rank deletion during puberty. (P) Percentage of fertile TAM-treated Rank^iUbc+/+^ mice and Rank^iUbcΔ/Δ^ male mice upon Rank deletion in adulthood (TAM treatment as shown in Fig S3I). Analyses were performed in 8-week-old mice (A–H, K–O) and 15-week-old mice (P). Data are represented as mean +/-SEM with each dot representing a mouse; P values were calculated by unpaired two-tailed t-test and indicated when statistically significant (A-H; L-O).

Whereas the role of Rank signaling in mammary gland development during pregnancy is well-established, its role during puberty, when mice undergo sexual maturation, remains unknown. To understand this aspect, we analyzed a constitutive Rank knockout mouse model lacking exons 4-7 (Rank^-/-^) (9). A strong defect in mammary gland invasion was observed in the absence of Rank (Fig. S1A). Defective mammary invasion was confirmed in a Rank knockout mouse model generated by deleting *Rank* exon 1, Rank^e1-/-^ (Fig. 1A). Rank^e1-/-^ mice displayed reduced body weight and osteopetrosis (Fig. S1B-C), mimicking previous Rank loss-of-function models (9). In contrast, depletion of Rank in the mammary epithelium (using a Rank^k5Δ/Δ^mouse model (10)), did not affect fat pad invasion (Fig. S1D). In fact, Rank^+/+^ or Rank^-/-^-derived mammary epithelial cells injected into the cleared fat pad of control mice, similarly invaded the fat pad (Fig. S1E).

As estradiol (E2) is the main ovarian hormone driving mammary invasion (11) and we observed an accumulation of estrogen-positive cells (ER+) in Rank^e1-/-^ mammary glands (Fig. S1F), we hypothesized that the reduced mammary gland development in Rank^e1-/-^ mice may be due to their E2 amounts (12). Indeed, we found a drastic reduction in E2 in Rank^e1-/-^ serum (Fig. 1B). However, concentration of cholesterol was similar in control and Rank^e1-/-^ serum (Fig. S1G), suggesting a defective synthesis of E2 instead of the lack of its precursor. Ovaries from Rank^e1-/-^ mice showed reduced mRNA expression of steroidogenesis proteins, responsible for E2 synthesis (Fig. S1H), while no changes were observed in other steroidogenic tissues, such as the adrenal gland (Fig. S1I). Histological analyses revealed an absence of corpora lutea in ovaries from most Rank^e1-/-^ females (up to 80% of analyzed mice) (Fig. 1C-D), denoting a deficiency in ovulation. Moreover, Rank-null mice had smaller (Fig. S1J) and lighter uteri than their control littermates (Fig. S1K), in line with the reduced E2 concentration. Together, these results demonstrate that full-body Rank deletion results in defective ovulation and E2 synthesis. Next, we analyzed whether Rank loss would also impact gonadal development in males. The presence of hypogonadism in Rank^-/-^ males was confirmed by the lower amounts of testosterone in serum, the reduced mRNA expression of testicular steroidogenic factors, the smaller testes and area of their seminiferous tubules (Fig. 1E-F, S2A-B).

As both, Rank-null males and females displayed hypogonadism, we wondered whether the hormones responsible for gonadal development, FSH and LH, might be altered. LH concentration was were reduced in serum from Rank^-/-^ males (Fig. 1G) and a reduction of approximately 60% in *Lhb* and *Fshb* mRNA expression was found in the pituitary glands, whereas other pituitary hormones such as *Prl, Gh* and *Tshb* were not altered (Fig. S2C). In Rank^e1-/-^ females, lower *Lhb, Fshb* and *Prl* mRNA amounts were detected in the pituitary gland (Fig. S2D). The reduction of *Prl* mRNA in females is likely driven by the reduction in E2, which regulates prolactin production (13). FSH and LH are regulated by GnRH released from hypothalamic GnRH neurons (7) and indeed, the analysis of *Gnrh1* mRNA in Rank^e1-/-^ hypothalamus pointed to a clear downregulation of this neuropeptide in male and females (Fig. 1H). In line with the role of GnRH neurons in the initiation of puberty, Rank^e1-/-^ mice displayed delayed balanopreputial separation and vaginal opening as external signs of puberty onset (Fig. S2E-F).

We then investigated RANK signaling in the human hypothalamus and its plausible role in the control of GnRH production, interrogating available transcriptomic data from healthy hypothalamus (GTEx, n = 170) (14). To assess RANK signaling activation status, we computed RANK metagene, which included the top 100 genes co-expressed with *RANK* mRNA (Table S1). RANK metagene strongly correlated with signatures of RANK pathway activation (r > 0.69) and with GnRH metagene (r = 0.69), whereas we found a lower correlation (r < 0.35) with other neuropeptide signaling molecules, such as growth-hormone-releasing hormone and corticotropin-releasing hormones (Fig. 1I, S2G-H, Table S1). Analyses of mouse hypothalamus corroborated the specific correlation of *Rank* with *Gnrh1* mRNA expression (Fig. S2I). In sum, these results strongly suggest a control of *Gnrh1* expression by RANK signaling. As a consequence, Rank loss induced severe HH in mice.

## Patients with congenital hypogonadotropic hypogonadism harbor rare *RANK* variants

Rank-deficient mice show physiological similarities with patients with CHH, who present defective pubertal development due to alterations in GnRH neurons. In approximately 50% of CHH cases, no specific gene mutation has been documented (15). Therefore, the identification of the whole spectrum of molecular alterations underlying the defective regulation of GnRH neurons in CHH remains to be elucidated. Whole-exome sequencing of 564 unrelated CHH probands identified six rare heterozygous variants in *TNFRSF11A* (RANK) in six unrelated probands who do not carry any known CHH gene defect, each with a minor allele frequency (MAF) < 0.1% in gnomAD v4.0 (16), collectively found in 1% of the cohort (n = 6), including four CHH individuals with anosmia (termed Kallmann syndrome) (Table 1). Two variants—p.K240E and p.E382G—met stringent in silico criteria for predicted pathogenicity and mapped to highly conserved residues (Fig. S3A, Table 1), suggesting functional consequences. The p.K240E variant (Proband B) segregated with delayed puberty in a sibling and was also present in an unaffected mother, consistent with incomplete penetrance and variable expressivity (Fig. S3B). Based on these findings, we expanded our analysis to rare predicted-deleterious variants in genes comprising the RANK metagene. We identified 76 unique heterozygous rare variants predicted to be pathogenic across 73 probands (13.1%) (Table S2), including 16 putative loss-of-function events. Several metagene components were recurrently affected, including *ATP8B4* (n = 7), *ENTPD4* (n = 5), *LRMP* (n = 4), *RGS3* (n = 4), *MEF2A* (n = 4), and *PTGS1* (n = 4). Two individuals carried multiple damaging variants in distinct metagene components, raising the possibility of oligogenic inheritance in a subset of CHH cases (Table S2). Notably, 70% of the probands harboring a rare variant in *RANK* or the *RANK* metagene did not carry rare variants in known CHH genes. Together, the identification of damaging variants in *RANK* and *RANK* metagene in CHH patients alongside the phenotypes observed in *Rank*-deficient mouse models supports a role for RANK signaling in the regulation of the human HPG axis.

## Postnatal Rank deletion suppresses the HPG axis in male mice, leading to infertility

Impaired migration of the GnRH neurons from the olfactory placode is one of the causes of CHH, which is accompanied by anosmia in patients with Kallmann syndrome (17). The fact that some CHH patients carrying *RANK* mutations are normosmic (Table 1) suggest that RANK may regulate GnRH neuronal homeostasis postnatally. To explore this hypothesis, we generated a tamoxifen (TAM) inducible loss-of-function mouse model (Rank^iUbcΔ/Δ^). Since TAM is a selective ER modulator (18), known to affect the HPG axis in females by itself (19), we restricted our analysis to TAM-treated male mice. Rank^iUbcΔ/Δ^ males and their control littermates Rank^iUbc+/+^ were treated with TAM for one week at pubertal onset and analyzed at adulthood (Fig. 1J). Unlike constitutive Rank-null mice, Rank^iUbcΔ/Δ^ males did not display osteopetrosis nor reduced body weight (Fig. S3C-D). TAM-treated Rank^iUbcΔ/Δ^ male were sterile or subfertile and showed reduced testicular weight (Fig. 1K-L, Fig. S3E), in contrast to control Rank^iUbc+/+^ TAM-treated and untreated Rank^iUbcΔ/Δ^ and Rank^iUbc+/+^ males. Fertility and gonadal weight were not altered in TAM-treated Rank^iUbc+/+^ mice, reflecting the minimal impact of TAM itself (Fig. 1K-L, Fig. S3E). Rank depletion postnatally reduced testosterone amounts (Fig. 1M) and the area of seminiferous tubules (Fig. S3F), suggesting impairment of spermatogenesis. Moreover, Rank^iUbcΔ/Δ^ males showed lower amounts of LH, reduced mRNAs of steroidogenic factors in testes and reduced mRNA of gonodotropins in the pituitary glands; while no major changes in non-gonadotropin hormones were observed (Fig. 1N, S3G-H). Importantly, *Gnrh1* and *Rank* mRNA amounts in Rank^iUbcΔ/Δ^ mice were reduced in the hypothalamus, confirming the depletion of *Rank* in the hypothalamic tissue (Fig. 1O). Next, we investigated the consequences of depleting Rank after mice had reached sexual maturation (Fig. S3I). Likewise, adult Rank deletion led to lower hypothalamic amounts of mRNA of *Gnrh1* and *Rank* and induced hypogonadism, reflected by reduced testicular weight and infertility in half of Rank^iUbcΔ/Δ^ mice (Fig. 1P, S3J-K). Together, these results corroborate that Rank signaling not only controls the HPG axis during embryogenesis, but also during puberty and adulthood, through a tight regulatory network.

## Microglia, the main source of Rank in the hypothalamus, regulates the HPG axis

**Fig. 2:**
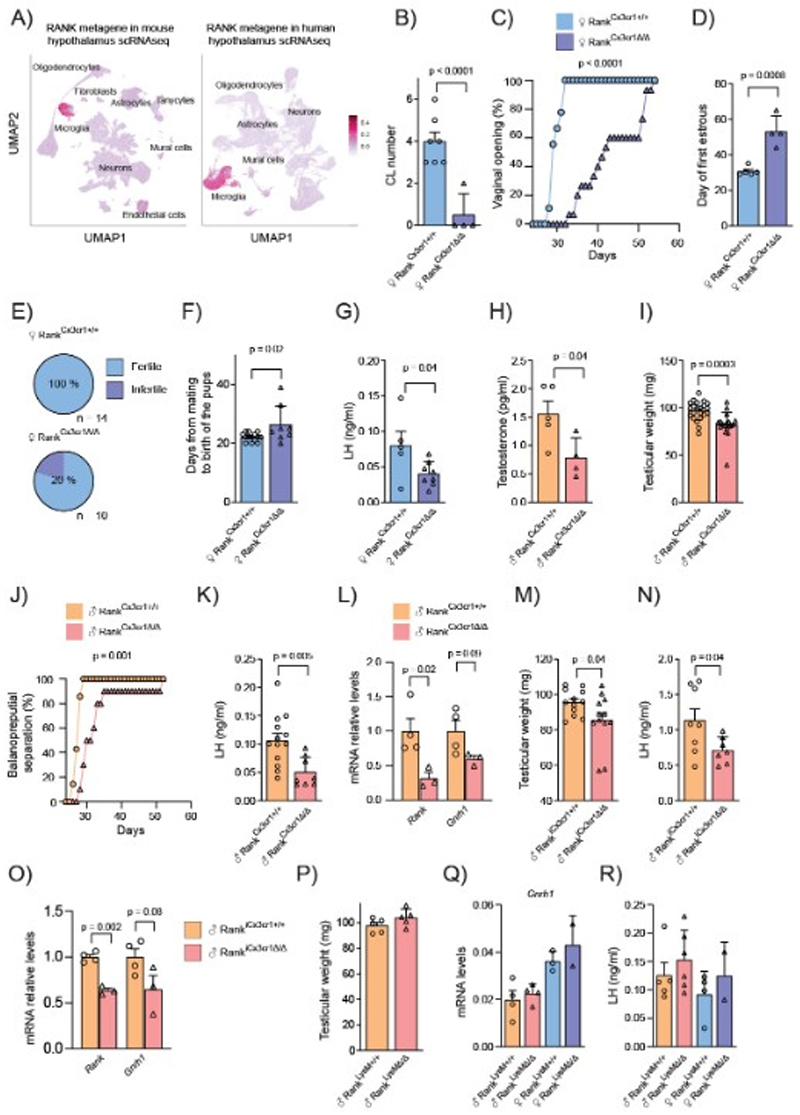
Microglia Rank loss impairs the HPG axis. (A) Left plot: RANK metagene expression in mouse hypothalamus from an integrated analysis of 17 scRNAseq datasets, including embryonic and adult mice (PMID: 36266547). Right plot: RANK metagene expression in human hypothalamus scRNAseq dataset from human embryos (GSE169109). (B) Quantification of corpora lutea (CL) in control and Rank^Cx3cr1Δ/Δ^ ovaries. (C) Graph showing the cumulative percentage of mice that achieved vaginal opening state. P-values calculated by the Gehan-Breslow-Wilcoxon matched-pairs test are shown (WT vs. Rank^Cx3cr1Δ/Δ^, χ^2^ = 22.45, P < 0.0001, n = 9 and n = 13). (D) Day of the first estrous cycle determined by daily vaginal smear after vaginal opening from control and Rank^Cx3cr1Δ/Δ^ females. (E) Percentage of fertile Rank^Cx3cr1+/+^ (n = 14) and Rank^Cx3cr1Δ/Δ^ female mice. (F) Days from mating to the birth of the pups of animals from panel E, excluding the infertile mice. (G) LH concentration in blood from Rank^Cx3cr1Δ/Δ^ and control Rank^Cx3cr1+/+^ females. (H) Testosterone concentration in serum from Rank^Cx3cr1Δ/Δ^ male mice and control littermates. (I) Testicular weight of Rank^Cx3cr1Δ/Δ^ male mice and control littermates. (J) Graph showing the cumulative percentage of males that achieved balanopreputial separation in Rank^Cx3cr1Δ/Δ^ male mice and controls. Statistical differences were tested using the Gehan-Breslow-Wilcoxon matched-pairs test: Rank^Cx3cr1+/+^ vs. Rank^Cx3cr1Δ/Δ^, χ^2^ = 7.897, P = 0.0034, n = 7 and n = 8. (K) LH concentration in blood from Rank^Cx3cr1Δ/Δ^ and control Rank^Cx3cr1+/+^ males. (l) mRNA amount of *Gnrh1* and *Rank* in the hypothalamus from Rank^Cx3cr1Δ/Δ^ relative to control male mice. (M) Testicular weight of Rank^iCx3cr1+/+^ and Rank^iCx3cr1Δ/Δ^ upon TAM treatment at puberty. (N) LH concentration in blood from Rank^iCx3cr1+/+^ and Rank^iCx3cr1Δ/Δ^ mice. (O) mRNA amount of *Rank* and *Gnrh1* mRNA expression levels in the hypothalamus from Rank^iCx3cr1Δ/Δ^ male mice and control littermates. (P) Testicular weight in Rank^LysMΔ/Δ^ and control littermates. (Q) *Gnrh1* mRNA amounts of Rank^LysM+/+^ and Rank^LysMΔ/Δ^ mice in hypothalamus from male and female mice. (R) LH concentration in blood from Rank^LysM/+^ and Rank^LysMΔ/Δ^ male and female mice. Analyses were performed in 8-week-old mice (B–R). Data are represented as mean +/-SEM with each dot representing a mouse; p values were calculated by unpaired two-tailed t-test and indicated when statistically significant (B, D, F-I, K-R).

Next, to assess whether the defects in the HPG axis observed upon Rank loss could be due to an intrinsic role of Rank signaling in GnRH neurons, we generated Rank^Gnrh1Δ/Δ^ mice. No defects in testicular or uteri weight, area of seminiferous tubules or presence of corpora lutea were found in Rank^Gnrh1Δ/Δ^ mice (Fig. S4A-D), indicating that Rank loss in GnRH neurons does not impair the establishment of the HPG axis. Therefore, we asked which Rank-expressing cell population in the hypothalamus might be responsible for regulating the GnRH neurons. Analysis of scRNAseq datasets from the mouse (20) and human hypothalamus (21, 22) revealed that the *Rank/RANK* genes and corresponding metagenes are almost exclusively expressed in the microglia, irrespectively of the sex (Fig. 2A, S5A-C). In fact, the RANK metagene exhibited enrichment in well-established microglia pathways, such as phagocytosis, and show a strong correlation with a scRNA-seq-derived-microglia gene signature (23), with nine overlapping genes (Fig S5D-F). We then hypothesized that Rank+ microglia might regulate the HPG axis. As there is no current evidence demonstrating a role for microglia in the regulation of GnRH neurons, we used the Csf1r inhibitor, PLX3397 to deplete microglia at puberty onset in control males and females and assessed its impact on the reproductive system. PLX3397 treatment eliminated microglia almost completely in the hypothalamus in both sexes (Fig. S6A). Microglial depletion by PLX3397 treatment disrupted the development of reproductive tissues, resulting in reduced testicular weight, a marked decrease in seminiferous tubular area, and a lower number of corpora lutea in the ovaries (Fig. S6B-D), supporting the role of microglia in the regulation of the HPG axis.

## Rank loss in microglia impairs the HPG axis in mice

To directly assess whether microglia regulate the HPG axis through Rank signaling, we generated two embryonic myeloid Rank loss-of-function mouse models: Rank^Csf1rΔ/Δ^ and Rank^Cx3cr1Δ/Δ^. Rank^Csf1rΔ/Δ^ mice, depleted for *Rank* in myeloid progenitors, showed a similar developmental phenotype as Rank-null mice (Fig. S7A-B). Meanwhile, Rank^Cx3cr1Δ/Δ^ mice displayed no apparent phenotype in the vertebral bone, although some mice showed defective tooth eruption and decreased body weight (Fig. S7C-D). In both models, the loss of Rank in microglia in females induced HH, similar to global Rank deletion, denoted by an almost complete absence of corpora lutea, reduced uteri weight, defective mammary invasion and increased frequency of ER+ cells in the mammary epithelia (Fig. 2B, S7E-I). The analysis of vaginal opening and the day of first estrous revealed a substantial delay in Rank^Cx3cr1Δ/Δ^ females (Fig. 2C-D). Indeed, adult Rank^Cx3cr1Δ/Δ^ female displayed irregular estrous (Fig. S7J) and were sterile (20% of them) or subfertile (Fig. 2E-F). In line with the observed dysfunctionalities of the HPG axis, Rank^Cx3cr1Δ/Δ^ females showed lower LH amounts and reduced gonadotrophins mRNA in the pituitary glands (Fig. 2G, S7K). In Rank^Csf1rΔ/Δ^ females, we did not observe changes in LH concentration, but found lower *Gnrh1* expression in the hypothalamus (Fig. S7L-M).

Similarly, Rank^Csf1rΔ/Δ^ and Rank^Cx3cr1Δ/Δ^ males displayed lower amounts of testosterone, decreased testicular weight and reduced area of seminiferous tubules (Fig. 2H-I and S8A-C), an indicator of impaired spermatogenesis. Despite, Rank^Cx3cr1Δ/Δ^ males remained fertile (Fig. S8D-E). Rank^Csf1rΔ/Δ^ and Rank^Cx3cr1Δ/Δ^ males exhibited a delay in the balanopreputial separation (Fig. 2J, Fig. S8F), indicating an impact of microglia Rank loss on pubertal onset also in males. Consistently, Rank^Cx3cr1Δ/Δ^ males also displayed lower LH amounts and reduced *Lhb*, but not *Fshb*, mRNA expression in the pituitary glands (Fig. 2K, S8G). Hypothalamic Rank deletion was confirmed in Rank^Cx3cr1Δ/Δ^ males and reduced *Gnrh1* expression was found in Rank^Csf1rΔ/Δ^ males (Fig. 2L, S8H). To confirm the specific role of microglia Rank signaling in the postnatal control of the HPG axis, we developed two independent TAM-inducible microglia loss-of-function mouse models: Rank^iCx3cr1Δ/Δ^ and Rank^iTmem119Δ/Δ^. Microglia Rank depletion at pubertal onset in males (Fig. 1J) did not affect body weight in these models (Fig. S8I-J). Rank^iCx3cr1Δ/Δ^ adult mice showed lower testicular weight, reduced seminiferous tubule area and fewer cells in the seminiferous epithelium, confirming defective testicular cytoarchitecture (Fig. 2M, S8K-L). In line with this defective gonadal development, lower amounts of LH along with a reduction of *Lhb*, but not of *Fshb*, mRNA expression in the pituitary tissue of Rank^iCx3cr1Δ/Δ^ males were observed (Fig. 2N, S8M), concomitant with a decrease in the hypothalamic expression of *Rank* (Fig. 2O). In Rank^iTmem119Δ/Δ^ males, we did not observe changes in testicular weight (Fig. S8N) or LH amounts (Fig. S8O), but hypothalamic *Rank* and *Gnrh1* mRNA expression was reduced (Fig. S8P). Together, results from the four independent mouse models, demonstrate that embryonic or postnatal microglia Rank loss leads to HH.

## Rank loss in peripheral myeloid cells does not impair fertility or sexual maturation

*Csf1r* and *Cx3cr1* models also display recombination in peripheral cells (24). To assess whether Rank loss in peripheral myeloid cells might contribute to the HH phenotype, we generated Rank^LysMΔ/Δ^ mice, which maintains *Rank* expression in the hypothalamus (Fig. S9A), but recombines efficiently in peripheral myeloid cells (24). Rank^LysMΔ/Δ^ mice displayed no changes in body weight, no evidence of bone defects and were fertile (Fig. S9B-E). The day of vaginal opening, the weight of uteri and testes, and gonadal histology (Fig. 2P, S9F-I) were not affected by the loss of Rank in peripheral myeloid cells. Additionally, comparable mammary invasion during puberty and similar ER+ cell frequency within mammary glands was observed in Rank^LysMΔ/Δ^ females (Fig. S9J-K). The amount of hypothalamic *Gnrh1* mRNA and LH concentration (Fig. 2Q, R) in Rank^LysMΔ/Δ^ mice was comparable to that of controls. Since Rank expression is limited to microglia in the hypothalamus, and the HH phenotype was observed in Rank^Csf1rΔ/Δ^, Rank^Cx3cr1Δ/Δ^ and Rank^iCx3cr1Δ/Δ^ mice, but absent in Rank^LysMΔ/Δ^ mice (where Rank is deleted in peripheral myeloid cells), our findings demonstrate that Rank signaling in microglia regulates *Gnrh1* mRNA expression and, in turn, modulates the HPG axis and fertility.

## Transcriptomic profiling at the single-cell level reveals reduced microglia activation upon Rank loss

**Fig. 3:**
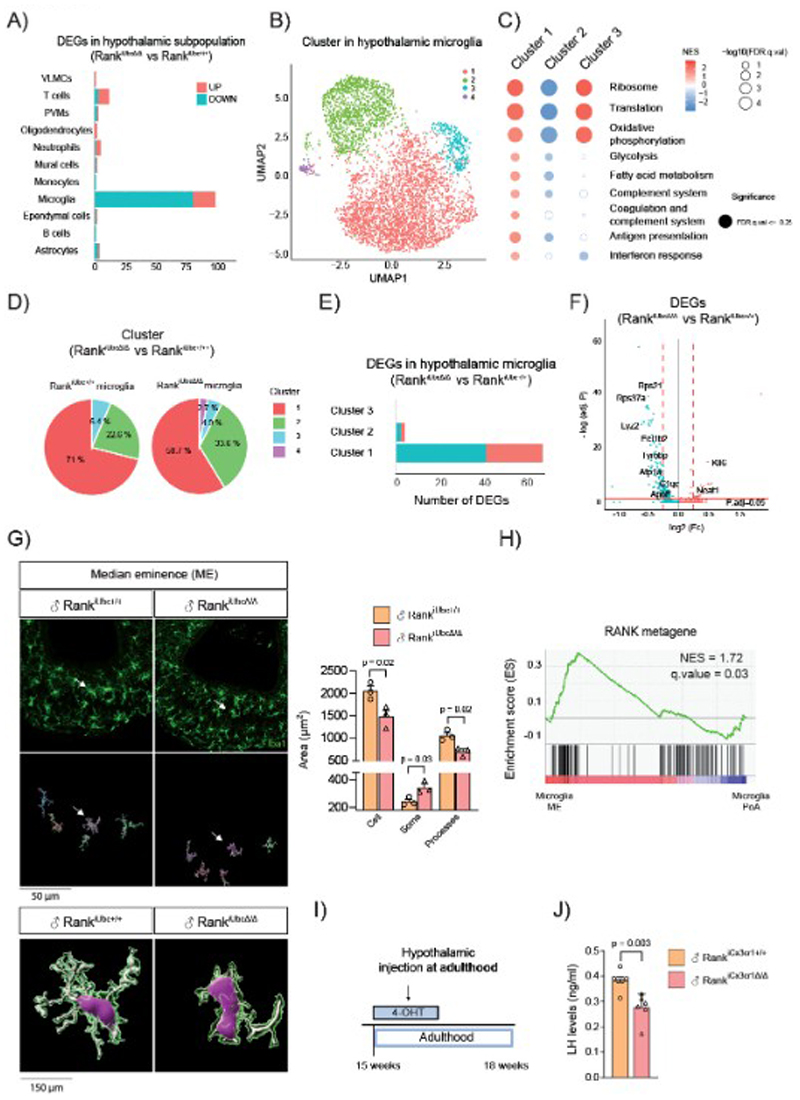
Loss of Rank induces transcriptomic changes in the microglia and alters its morphology in the ME. (A) Bar plots showing differential expression genes (DEGs) upon pubertal Rank loss between Rank^iUbcΔ/Δ^ and Rank^iUbc+/+^ male mice within each hypothalamic cluster by scRNAseq. VLMCs = Vascular and leptomeningeal cells, PVMs = Perivascular macrophages. (B) UMAP of hypothalamic microglia showing four different subpopulations by the combination of Rank^iUbcΔ/Δ^ and Rank^iUbc+/+^ cells upon Rank deletion in puberty. The fourth microglia cluster did not pass the quality control due to its limited abundance (57 out of 2100 microglia cells) and the expression of the female-specific gene *Xist* in males (C) Bubble plot depicting the variation of selected gene set signatures from cluster 1, 2 and 3. Filled bubbles indicate enriched significant gene signatures (FDR < 0.25). Red and blue colors indicate upregulated (NES>0) and downregulated (NES<0). (D) Circle charts of all microglia clusters by percentage in Rank^iUbcΔ/Δ^ and control mice. (E) Bar plot showing differentially expressed genes (DEGs) between Rank^iUbcΔ/Δ^ and Rank^iUbc+/+^ mice across microglia clusters. Blue part indicates downregulated genes, and red part indicates upregulated genes. (F) Volcano plots of DEGs in Rank^iUbcΔ/Δ^ versus Rank^iUbc+/+^ in microglia. Significant DEGs above the horizontal red line based on P-adjusted value < 0.05, blue and red bubbles represent significantly downregulated or upregulated genes, respectively. Fc, fold-change. (G) Representative immunofluorescence (IF) images of hypothalamic microglia cells (Iba1+ in green) in the median eminence (ME) from Rank^iUbcΔ/Δ^ and Rank^iUbc+/+^ male mice upon pubertal Rank loss, Reconstructed microglia images generated using IMARIS software (marked with white arrow) and quantification of microglial morphological parameters including total microglial area, cell body area and processes area are shown. (H) GSEA of the RANK hypothalamic metagene between ME and preoptic area (PoA) microglia. (I) TAM treatment procedure to delete Rank in the ME. Mice (12-15 weeks) were treated at adulthood with 10 mM 4-hydroxytamoxifen (4-OHT) intracranially in the ME and were euthanized 4 weeks after the first treatment. (J) LH concentration in blood from Rank^iCx3cr1Δ/Δ^ and control littermates after 4 weeks of intracranial injection with 4-OHT. Data are represented as mean ± SEM, with each dot representing a mouse, P values were calculated using unpaired two-tailed t-test and indicated when statistically significant (G, J). Rank^iUbcΔ/Δ^ and Rank^iUbc+/+^ male mice were TAM treated as shown in Fig. 1J (A-G)

To elucidate the molecular consequences of Rank loss on the microglia, single-cell transcriptional profiling was performed on the hypothalamus from control and Rank^iUbcΔ/Δ^ males four weeks after pubertal Rank deletion. A pool of five hypothalami was analysed after a Percoll centrifugation step to enrich for microglia. Following quality control filtering, 5,807 from control and 3,990 Rank^iUbcΔ/Δ^ cells were sequenced (70,000 reads/cell). Clustering analysis using Uniform Manifold Approximation and Projection (UMAP) and known hypothalamic cell types markers (20, 25–27) revealed twelve hypothalamic clusters including microglia, astrocytes, oligodendrocytes, among others (Fig. S10A-B). Rank^iUbcΔ/Δ^ microglia displayed the highest number of differentially expressed genes (DEGs), while other hypothalamic populations, such perivascular macrophages (PVMs) or astrocytes, displayed very few DEGs, reinforcing the notion that microglia is the most affected hypothalamic population upon Rank loss (Fig. 3A, Table S3).

In the microglia cluster, three main subpopulations were found (Fig. 3B). Cluster 1 represents the predominant population enriched in common microglial homeostatic genes, such as *P2ry12* and *Cx3cr1*, as well as phagocytic genes, including *C1qa* and *Lys2* (Fig. S10C). Gene set enrichment analysis (GSEA) indicated an active population with higher protein synthesis, metabolism, and complement system pathways (Fig. 3C, Table S4). Indeed, cluster 1 shares transcriptional similarities with metabolic and proliferative microglia found in embryogenesis, during inflammatory response and in aging (28, 29) (Fig. S10D, Table S4). Cluster 2 displays a “mirror” image of cluster 1, denoting an “inactive” microglia subcluster with downregulation of most pathways analyzed, including translation and metabolism, and negative associations with proliferative and inflammatory pathways (Fig. 3C, S10D, Table S4). Cluster 2 is characterized by the expression of the transcription factor, *Mef2a*, a regulator of microglia development (30), and the microglia-specific receptor *Tgfbr1* (31) (Fig. S10C). Cluster 3 represents metabolically active microglia, but with less inflammation and a notable enrichment in ribosomal and respiratory pathways (Fig. 3C, S10D, Table S4).

Comparison of the relative frequencies of microglial clusters between genotypes revealed that Rank loss leads to a reduction in the “active” cluster 1 (from 71% to 58%), accompanied by an increase in the “inactive” cluster 2 abundance (from 22.6% in controls to 33.6% in Rank^iUbcΔ/Δ^ mice) (Fig. 3D). Moreover, the majority of DEGs and pathways were found downregulated in the whole microglia and in cluster 1 upon Rank loss (Fig. 3A, 3E-F, Table S3, S5), supporting the role of Rank signaling in maintaining active hypothalamic microglia. Rank^iUbcΔ/Δ^ microglia showed downregulation of key mediators of the Trem2/Tyrobp signaling pathway (*Tyrobp, ApoE*), the complement system (*C1qa, C1qc)*, phagocytic system (*Lyz2, Gpr84, S100a8, S100a9, Ctss*), protein translation (*Eef1b2, Eef1d)* and mitochondrial respiration (Fig. 3F, S10E, Table S3). Similar changes were observed specifically within cluster 1 upon Rank loss, where several mediators of phagocytosis, complement system, translation and metabolism were reduced (Fig. S10F, Table S5). Together, the reduction in the relative frequency of cluster 1 and specific changes within this cluster suggest a general reduction in microglia activation upon pubertal Rank loss. Rankl stimulation in primary microglia cultures from mice, as well as in the BV2 microglia cell line, led to the upregulation of established RANK/NF-κB target genes such as *Birc3* and *Nfkb2* as well as key mediators of the Trem2/Tyrobp phagocytic signaling pathway, including *C1qc, ApoE* and *LysM* genes (Fig. S10H), reinforcing Rank signaling as a regulator of microglia activation. Altogether, these results reveal three distinct states of hypothalamic microglia and expose the central role of Rank signaling in the maintenance of homeostatic microglia with higher metabolic and phagocytic activity.

## Rank loss induces morphological alterations in microglia of the median eminence

Transcriptomic analyses revealed the central role of Rank signaling in the maintenance of microglia activation. Then, we assessed whether and how Rank loss affects the number and morphology of microglia in different hypothalamic regions where GnRH neurons reside. No changes in the number of microglia cells (Iba1+) or morphology were found in Rank^iUbcΔ/Δ^ mice in the PoA, where most GnRH cell bodies are located (Fig. S11A-B), suggesting a minimal impact of Rank loss in this area. Next, we evaluated microglia in the mediobasal hypothalamus, where the GnRH neuron terminals are located and secrete their transmitters into the ME. We focused on the ME and on the arcuate nucleus (ARC), as Kiss1 neurons in the ARC regulate the pulsatile secretion of GnRH neurons. An increase in the number of microglia was found in the ME, but not the ARC of Rank^iUbcΔ/Δ^ mice (Fig. S11C). Similarly, Rank^iUbcΔ/Δ^ mice exhibited a microglia smaller area, bigger soma, and less processes in the ME, but not in the ARC (Fig. 3G, S11D, movies S1-S2). Although Rank-deficient microglia exhibit amoeboid morphology, typically associated with activation, transcriptomic analyses indicate a hypoactive state (Table S3), highlighting that morphology alone does not predict functional activation. Indeed, analysis of astrocyte area in the ME revealed no differences between control and Rank-depleted mice discarding astroglial activation or inflammation (Fig. S11E).

To confirm that morphological changes are induced by Rank loss in microglia, we analyzed the microglia morphology of the ME in Rank^iCx3cr1Δ/Δ^ mice crossed with the tdTomato reporter mouse line upon TAM treatment at puberty, which shows high recombination efficiency (tdtomato+/Iba1+) in the ME (Fig. S12A). Similar to the phenotype seen with ubiquitous Rank deletion, Rank^iCx3cr1Δ/Δ^ mice displayed smaller microglia in the ME with fewer processes and larger soma (Fig. S12B), indicating that microglia Rank loss induces morphological changes in the ME microglia, consistent with alterations in microglia activation (32).

We hypothesized that Rank signaling may be more active in the ME microglia and therefore particularly sensitive to Rank loss. Indeed, we integrated two public scRNA-seq datasets: one from the PoA (33) and another from the ME (34). UMAP analysis of the combined microglia populations from both datasets revealed a clear distinction between ME and PoA microglia (Fig. S12C). Although Rank is expressed in microglia from both regions (Fig. S12D), ME microglia exhibited enrichment of the RANK metagene, indicating increased RANK pathway activity in this area (Fig. 3H). To demonstrate the importance of microglia Rank signaling in this region, we injected 4-hydroxytamoxifen into the ME of adult Rank^iCx3cr1Δ/Δ^ and control mice crossed with the tdtomato reporter line (Fig. 3I). The presence of recombined microglia cells in the ME (Iba+/tdtomato+) of Rank^iUbcΔ/Δ^ confirmed the depletion of Rank in this hypothalamic population (Fig. S12E). We also observed a reduction of LH amounts in Rank^iCx3cr1Δ/Δ^ mice (Fig. 3J), indicating a disruption in the HPG axis. Together, morphological assessments reveal a central role of Rank signaling in the regulation of microglia activation, particularly in the ME, a hypothalamic area crucial for the control of the reproductive axis.

## Microglia Rank loss impairs GnRH neuronal function via reduced microglia-GnRH neuron interactions in the ME

We established that Rank loss induces microglia morphological changes in the ME, accompanied by a transcriptomic profile indicative of less active microglia. We then aimed to determine how GnRH neurons are affected. First, we assessed the impact of embryonic Rank loss in the microglia on GnRH neuron numbers. We performed whole-brain tissue clearing and immunostaining against GnRH using iDISCO (immunolabeling-enabled three-dimensional imaging of solvent-cleared organs) in Rank^Cx3cr1Δ/Δ^ males. We did not observe any apparent differences in the number of GnRH neurons in the whole brain (Fig. S13A). Quantification of GnRH neurons in the PoA, where most GnRH neurons reside, from independent Rank^Cx3cr1Δ/Δ^ male mice confirmed these results (Fig. S13B), indicating that embryonic Rank deletion in microglia does not affect GnRH neuronal migration. Accordingly, no differences in sniffing capacity were observed in Rank^Cx3cr1Δ/Δ^ mice in habituation-dishabituation tests (Fig. S13C), a phenotype typically seen in mice with CHH and deficiencies in GnRH neuronal migration. As expected, in adult Rank^iUbcΔ/Δ^ mice, GnRH neuronal numbers in the PoA were not altered, and no differences in odor tests were observed (Fig. S13D-E). These results highlight that Rank deletion does not alter the number of GnRH neurons, nor affect their migration. Instead, the regulatory role of Rank appears to be directly related to GnRH neuronal functionality.

**Fig. 4:**
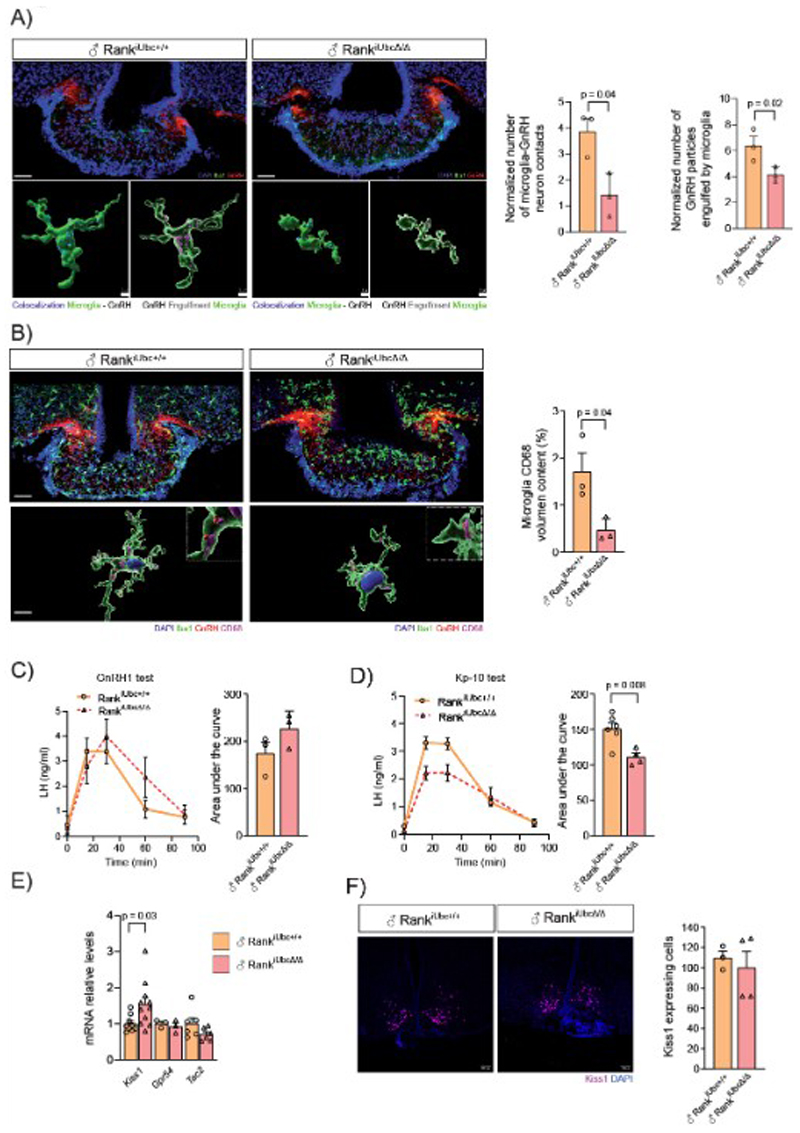
Rank loss reduces GnRH neuron-microglia contacts and phagocytosis and disrupts GnRH function. (A) Top: Representative immunofluorescence (IF) images of microglial cells (Iba1+, green) and GnRH neurons (GnRH, red) in the median eminence (ME) of control and Rank^iUbcΔ/Δ^ mice upon pubertal Rank loss. Nuclei are labeled with DAPI (blue). Bottom: Reconstruction of microglia cells using IMARIS in the ME of control and Rank^iUbcΔ/Δ^ mice. Colocalization (left, blue) indicates areas of interaction between microglia and GnRH immunoreactive particles. Colocalization (right, pink) indicates engulfment of GnRH particles inside microglia. Bar blots show quantification of GnRH neuron-microglia interactions and GnRH engulfment by microglia normalized to microglia cell area. (B) Top: Representative IF images of microglia cells (Iba1+, green) GnRH neurons (GnRH, red) and CD68 (pink) in the ME of control and Rank^iUbcΔ/Δ^ mice. Nuclei are labeled with DAPI (blue). Bottom: Reconstruction of microglia cells using IMARIS in the ME of control and Rank^iUbcΔ/Δ^ mice with GnRH content (red) and CD68 (pink). Quantification of percentage of CD68 within microglia volume is shown. (C) Time-course of LH levels in response to GnRH treatment in Rank^iUbcΔ/Δ^ and control male mice (n = 3). Right panel shows the quantification of area under the curve (AUC). (D) Time-course of LH levels in response to Kisspeptin-10 (Kp-10) treatment in Rank^iUbc+/+^ (n = 6) Rank^iUbcΔ/Δ^ (n = 3) male mice upon Rank deletion at puberty. The right panel shows the quantification (AUC) from the graph on the left panel. (E) Gene expression of *Kiss1*, kisspeptin receptor (*gpr54*) and neurokinin B (*Tac2*) in control and Rank^iUbcΔ/Δ^ hypothalamus upon Rank deletion at puberty. (F) Number of Kiss1 neurons by RNAscope in control and Rank^iUbcΔ/Δ^ hypothalamus. Number of Kiss1+ clusters was quantified in the ARC area of the mediobasal hypothalamus. Data are represented as mean ± SEM, with each dot representing a mouse, P values were calculated using unpaired two-tailed t-test and indicated when statistically significant (A-F). Rank^iUbcΔ/Δ^ and Rank^iUbc+/+^ male mice were TAM treated as shown in Fig. 1J.

Next, we evaluated how the less active microglia observed upon Rank loss might communicate with GnRH neurons. In males, ARC Kiss1 neurons communicate with GnRH neurons at the level of their terminals near the ME (35). We observed a reduction in microglia–GnRH nerve terminal contacts in the ME of Rank^iUbcΔ/Δ^ mice (Fig. 4A, movies S3-S4). Additionally, microglia in Rank-deficient mice showed reduced engulfment of GnRH-immunoreactive content, suggesting decreased remodeling of GnRH neuroendocrine terminals (Fig. 4A) likely due to diminished microglia activity following Rank loss, as observed in our scRNAseq analyses. Analysis of the phagocytic marker CD68 revealed fewer phagosomes within microglia, indicating reduced phagocytosis (Fig. 4B). Fewer GnRH neuron–microglia contacts in the ME and reduced engulfment of GnRH within microglia were also observed in microglia-specific Rank-deficient mice (Rank^iCx3cr1Δ/Δ^), highlighting that this phenotype results from the loss of Rank specifically in microglia (Fig. S14).

The function of GnRH neurons is to secrete GnRH in a pulsatile way into the pituitary gland. The main regulators of the pulsatile secretion of GnRH neurons are the subset of Kisspeptin/NeurokininB (NKB)/Dynorphin (KNDy) neurons in the ARC, near the ME. Therefore, we examined whether and how the GnRH/kisspeptin neuronal system was affected upon Rank loss. We subjected male Rank^Cx3cr1Δ/Δ^, Rank^iUbcΔ/Δ^ and corresponding controls to treatment with exogenous Kisspeptin-10 (Kp-10) (36) and GnRH treatment and analyzed LH amounts *in vivo*, as a bona fide marker of GnRH-pituitary (gonadotroph) functionality. These pharmacological tests showed a preserved pituitary responsiveness in Rank^iUbcΔ/Δ^/Rank^Cx3cr1Δ/Δ^ mice to exogenous GnRH (Fig. 4C and Fig. S15A). In contrast, a defect in the response to Kp-10 was observed in both Rank^iUbcΔ/Δ^ and Rank^Cx3cr1Δ/Δ^ mice (Fig. 4D and Fig. S15B), supporting a primary defect at the GnRH neuron level. To confirm such impaired GnRH neuronal function, assessment of LH pulsatility test, a standardized readout of GnRH neuronal activity, was performed, showing a reduced pulse frequency in Rank^iCx3cr1Δ/Δ^ mice (Fig. S15C-D).

No changes in *Tac2* (encoding NKB) mRNA or NKB protein amounts were observed in Rank^iUbcΔ/Δ^ hypothalamus (Fig. 4B and Fig. S15E). Notably, in Rank^iUbcΔ/Δ^ hypothalamus, *Kiss1* mRNA expression was upregulated, but the number of Kiss1 neurons or the gene expression of kisspeptin receptor, *Gpr54* were not altered (Fig. 4E-F).The increase in *Kiss1* mRNA amounts was confirmed in the hypothalamus of microglia Rank-depleted mice (Rank^Csf1rΔ/Δ^ and Rank^iCx3cr1Δ/Δ^; Fig. S15F-G), and no changes in *Tac2* expression was found in Rank^iCx3cr1Δ/Δ^ mice. The increased *Kiss1* mRNA expression likely reflects a compensatory response to the impairment of GnRH neuronal function. Together, these findings indicate that Rank loss induces both morphological and functional alterations in ME microglia, leading to less phagocytic microglia and reduced contacts with GnRH nerve terminals. This impaired microglia–GnRH neuron interaction disrupts GnRH neuron function and responsiveness to Kiss1 signaling, without changes in the number of Kiss1 neurons, ultimately resulting in hypogonadism through dysfunction of the HPG axis.

## Discussion

Our current data from ten independent Rank-deficient mouse models, human hypothalamic samples, patients with CHH and scRNAseq data provide strong evidence that microglia Rank signaling is a central regulator of sexual maturation and fertility through the control of GnRH neuronal function. Using mouse models, displaying either global or microglia-restricted Rank depletion, we demonstrate that microglia Rank loss impairs microglia activation, alters microglia morphology in the ME and their interactions with GnRH neurons, disrupting GnRH neuronal functionality and leading to HH. Of note, constitutive full and microglia Rank knockout mice exhibited bone defects and reduced body weight that might influence the hypogonadal phenotype (37). To avoid such confounding effects, we developed inducible mouse models allowing timed deletion of Rank (Rank^iUbcΔ/Δ^, Rank^iC3xcr1Δ/Δ^ and Rank^iTmem119Δ/Δ^), which also resulted in a defective regulation of the HPG axis, without impacting body weight nor bone homeostasis. Compelling evidence has documented that the influence of reduced body weight on reproduction is mainly due to changes in different neuroendocrine signals, including low leptin amounts in circulation and reduced *Kiss1* expression (38). Thus, although reduced body weight in global or microglia Rank-null mice might contribute to the suppression of the HPG axis *per se*, the fact that downregulation of *Gnrh1* mRNA also occurred in Rank^iUbcΔ/Δ^ or Rank^iCx3cr1Δ/Δ^ mice, which did not show reduced body weight, argues against the possibility that the impairment of the HPG axis due to Rank ablation is merely secondary.

We unveiled that Rank signaling regulates GnRH neurons within the hypothalamus and, consequently, the HPG axis, not only during puberty, but also in adulthood, indicating that Rank signaling regulates fertility even after sexual maturation. Previous studies have shown that Rank signaling is essential for testicular development (39); however, its role in the regulation of GnRH neuron had not been clarified. In women, genome-wide association studies revealed single nucleotide polymorphisms in *RANK* and *RANKL* genes, which have been linked to a delay in the onset of the first menstrual cycle as well as menopause (40–42). These data support a functional role for human RANK signaling throughout the female reproductive cycle.

Rank-deficient females exhibit a more profound HH than males, showing substantial delays in pubertal onset, infertility, and absence of ovulation, whereas males retain sperm production and are fertile. This may be attributed to sex-specific differences in the regulation of GnRH neurons or in microglia, which are known to differ between males and females (43, 44), highlighting the need for a more thorough study of the mechanisms of GnRH neuron between sexes. One of the limitations of the inducible loss-of-function mouse model is its dependency on TAM treatment. In females, TAM impairs the HPG axis even at low doses (45), which prevented us from properly assessing fertility phenotypes caused by postnatal Rank loss in females. TAM also affects the male HPG axis and, at high doses induces defective spermatogenesis and hypogonadism (46, 47). Although we acknowledge that TAM treatment may affect our experimental system, the fact that control mice treated during puberty remain fertile and do not display hypogonadism suggests that TAM has only a minimal impact on the HPG axis under our treatment protocol.

HH is also promoted by mutations in the GPR54 (49, 50). Kisspeptins, endogenous peptide ligands of GPR54, are secreted by Kiss1 neurons and constitute the main activators of GnRH secretion. Our data demonstrate that Rank controls the function of the HPG axis primarily at the level of GnRH neurons and not Kiss1 neurons, as a defective response of GnRH neurons was observed under pharmacological treatment with Kp-10, the most potent GnRH secretagogue known to date. Despite the number of Kiss1 neurons was not altered, we found an increase in *Kiss1* gene expression in the hypothalamus. The latter seemingly represents a compensatory mechanism to overcome the suppression of GnRH secretion, which is likely driven by the elimination of the negative feedback of sex steroids hormones upon microglia Rank ablation. In fact, sexual hormones have been shown to suppress *Kiss1* expression in the mediobasal hypothalamus (51, 52); hence, HH in Rank-depleted mice is expected to enhance *Kiss1* expression. Yet, in the absence of Rank, *Gnrh1* expression is persistently low despite the apparent compensatory rise of kisspeptin drive. Rank signaling pathway is therefore key to the central control of the HPG axis, via regulation of GnRH neurons, thus unveiling potential therapeutic targets for syndromes bound to infertility. CHH is a rare disorder that results from the failure of GnRH secretion, either due to defective migration of GnRH neurons or perturbation of GnRH neuronal homeostasis. In both cases, CHH is linked to delayed/absent puberty and infertility (17). CHH is characterized by vast genetic heterogeneity (53). We have now identified gene variants in *RANK* and in the genes comprising the *RANK* metagene in CHH patients. Although we did not perform functional studies on *RANK* mutations, two of these mutations (p.K240E, p.E382G) were predicted to be damaging, supporting their involvement in RANK protein function. The variable expressivity and incomplete penetrance in one family carrying *RANK* mutations are consistent with a complex interaction between RANK pathway and the network of genes regulating the HPG axis.

Moreover, our results expose a link between Rank signaling and microglia functionality, as well as the involvement of microglia in the regulation of GnRH neuron. Although the main regulation of GnRH neuron is attributed to neurons, such as Kiss1 neurons (54), evidence suggests that glial cells, mainly tanycytes and astrocytes, could contribute to this regulation (55–57). Despite an association between hypothalamic microglia and GnRH neurons has been suggested (58–60), mechanistic evidence for such a connection was missing. Microglia cells are the primary immune cells of the central nervous system, similar to macrophages (61), and were originally thought to be a dormant population (62). However, microglia is also involved in physiological functions, such as mediating the communication between neuronal cells (63). Building on the knowledge that Rank pathway is active in microglia (64–66), we now demonstrate that microglia of the hypothalamus regulate GnRH neuron function through Rank signaling. Although scRNAseq analyses have effectively described neuronal subpopulations at the hypothalamic level, the characterization of microglial subpopulations remains limited due to microglia scarcity (20). Our results now reveal that hypothalamic microglia exist in three distinct stages, with the most abundant being an activated state that is highly dependent on Rank signaling. Rank depletion not only reduces the abundance of the activated microglia subpopulation, but also compromises its functionality by impairing complement activation, protein translation and energy homeostasis. Alterations in protein synthesis disrupt microglia priming, phagocytosis, motility and synapse regulation (67, 68).

Rank loss causes profound transcriptomic changes in the microglia and morphological alterations in the ME. The ME is the projection field of GnRH neurons, which plays a precise regulation of GnRH release. The amoeboid morphology caused by Rank loss in the ME is suggestive of hyperactive/inflamed microglia. Paradoxically, the reduction in microglia-GnRH neuron contacts, GnRH immunoreactive particles engulfment by the microglia and the phagocytic marker CD68 is indicative of dysfunctional/hypoactive microglia with no signs of inflammation, in agreement with the transcriptomic results. Accumulating evidence indicates that microglia functionality cannot be inferred exclusively from their morphology and must be complemented by transcriptomic and functional analyses (69). Our data provide evidence that engulfment of GnRH nerve terminals by hypothalamic microglia in the ME is essential for their functionality and for the regulation of the HPG axis, in line with the known role of microglia in regulating synaptic communication and pruning of specific neurons (70).

In sum, our comprehensive studies in mouse models and human samples support that Rank-expressing microglia are key regulators of microglia activation, GnRH neuron functionality and, in turn, sexual development and fertility, uncovering RANK as a potential therapeutic target in endocrine disorders and fertility syndromes, as well as a candidate gene for molecular diagnosis of CHH disorder.

## Materials and Methods

### CHH Patients

The CHH study group consisted of 564 individuals (286 with Kallmann syndrome and 278 with normosomic CHH). The data are from the clinical trial NCT01601171 (https://clinicaltrials.gov/study/NCT01601171?locn=CHUV%20Centre%20Hospitalier%20Universitaire%20Vaudois&cond=gnrh&rank=2). Data are collected at the Centre Hospitalier Universitaire Vaudois (CHUV), in Lausanne, Switzerland. Recruitment and data collection period: 2012-03 - 2030-03. Primary outcome measure: Rare sequence variant(s) in gene(s). Secondary outcome measures: functionality of identified rare sequence variants (mutations), mode of inheritance, genotype-phenotype correlation. The diagnosis of CHH was determined by three factors: i) absence or incomplete development of puberty by the age of 17; ii) low or normal levels of gonadotropins with low levels of testosterone or estradiol; and iii) abnormal function of the anterior pituitary gland and abnormal imaging of the hypothalamic-pituitary region (71). Olfaction was assessed through either self-reported information or formal testing (72). Genetic testing was conducted on probands and their family members whenever available. This study was approved by the ethics committee of the University of Lausanne and all participants provided written consent prior to their involvement.

### CHH Genetic analyses

We extracted genomic DNA from peripheral blood samples using the Puregene Blood Kit (Qiagen) as per the manufacturer’s instructions. Exome capture was carried out using SureSelect All Exon capture v2 or v5 (Agilent Technologies), and the samples were sequenced on the HiSeq2500 (Illumina) at BGI (BGI, Shenzen). We used an in-house pipeline to analyze the raw sequences (FASTQ files) that utilized the Burrows-Wheeler Alignment algorithm (BWA) (73) for mapping the reads to the human reference sequence (GRCh38) and the Genome Analysis Toolkit (GATK) (74) to detect single nucleotide variants (SNVs) and insertion/deletions. The identified variants were annotated using Annovar version 20191024 (75) and gnomAD v4.0 for minor allele frequency (MAF), CADD (76) and AlphMissense (77) for pathogenicity scores. We selected variants passing multiple quality filters, with a coverage and quality score higher than 10 and 30 respectively, an allelic depth ratio higher than 0.2, and not present in segmentally duplicated regions (78). Furthermore, rare variants present frequently in a local genetic database of healthy individuals (n=300), in excess of five pedigrees in the cohort, or flagged in gnomAD were considered systematic sequencing artifacts and discarded. We established a MAF threshold of 0.1% and excluded all variants with a higher popmax MAF in gnomAD. This is accounting for CHH prevalence in the general population (71) and recessive genes. For RSVs in RANK and RANK metagenes, we further applied pathogenicity filtering by retaining variants with either a CADD score > 20 or an AlphaMissense score > 0.5, in order to exclude likely benign variants. In addition, mutations in known CHH genes, as assessed by ACMG criteria, were investigated in our CHH cohort.

### Mice

All research involving animals was performed at the IDIBELL and CNIO Animal Facilities in compliance with protocols approved by the IDIBELL and CNIO Committees on Animal Care and following national and European Union regulations. All animal experiments were approved by the ethical Committee for Animal Experimentation (CBA07_2021) from the Autonomous Community of Madrid. Rank flox/flox (Rank^fl/fl^) (MGI:J:219915) mice were provided by Dr. Joseph Penninger (79) and crossed with K5-Cre (MGI:1926815) to generate Rank^K5Δ/Δ^, Csf1r-Cre (MGI:5763756) to generate Rank^Csf1rΔ/Δ^ (80), Gnrh1-Cre (MGI:3691288) to generate Rank^Gnrh1Δ/Δ^, Cx3cr1-Cre (MGI:5467983) (81) to generate Rank^Cx3cr1Δ/Δ^, LysMCre (MGI: 1934631) to generate Rank^LysMΔ/Δ^, UbcCreERT2 (MGI:1926815) to generate Rank^iUbcΔ/Δ^, Cx3cr1-CreERT2 (MGI:6724385) to generate Rank^iCx3cr1Δ/Δ^ and Tmem119-CreERT2 (MGI:6758066) to generate Rank^iTmem119Δ/Δ^ mouse models. The resulting Rank^iCx3cr1Δ/Δ^ mouse model was crossed with the reporter mouse line Rosa26tdtomato (MGI 155793) to label the recombinant cells. Rank^-/-^ mice (MGI1314891) were previously characterized (82). Some mouse lines used in this study were obtained under a Material Transfer Agreement and may be shared upon reasonable request by contacting the corresponding author, subject to approval by the material owner.. Rank^-/-^, Rank^ex1-^/-, and Rank^Csf1rΔ/Δ^ mice display osteopetrotic phenotype and absence of tooth eruption. Therefore, these mice were provided with a special liquid diet, which was changed daily, and maintained together with their littermates. Some Rank^Cx3cr1Δ/Δ^ mice also show defective teeth; therefore, all mice were maintained on the same diet Genotyping primers are indicated in Table S6. Analyses of mouse samples were designed to have a minimum of n ≥ 3. Based on previous experience, this sample size is considered suitable for obtaining confident results.

### Generation of Rank^e1-/-^ mice

The Rank^e1-/-^ mouse model was generated by inserting a CreERT2-IRES-EGFP-pA construct, followed by a frt-flanked PGK-neo cassette, immediately downstream of the ATG translation initiation codon of the *Rank* gene. The targeting vector was electroporated into G4 mouse embryonic stem cells, and clones were selected in 200 μg/ml of G418. Homologous recombinant clones were identified by Southern blot analysis of genomic DNA digested with the restriction enzymes EcoRV (5’ recombination) and KpnI (3’ recombination) using 5’ and 3’ external probes amplified from genomic DNA. The primers used to amplify the 5’-1 probe (335 bp) by PCR were as follows: Forward: 5’-ATTGTGGGAGGGGTAACTGG-3’ and Reverse: 5’-AAAAGAAAACAGAGCAGCGGG-3’. The primers used to amplify the 3’-2 probe (372 bp) were as follows: Forward: 5’-GCCATGAGTTCAACCCCTCA-3’ and Reverse: 5’-CACGAGAGGTCTGGCTTGTT-3’. The band sizes obtained for the 5’ homologous recombination were 7.2 kb (KI) and 18.0 kb (WT), and for the 3’ homologous recombination, 15.0 kb (WT) and 11.0 kb (KI). Chimeras were generated by microinjection into CD1 8-cell embryos. Germ line male chimeras were crossed with Tg.CAG-Flpe females to generate offspring in which the selection cassette was deleted. Deletion was confirmed by Southern blot analysis of genomic DNA digested from ear punch biopsies with DrdI and using a DNA fragment of 361 bp (probe 3’-1) as a probe. The probe 3’-1 was amplified by PCR from genomic DNA using the following primers: Forward: 5’-TTTTCAGGGTGGAGCATCCC-3’ and Reverse: 5’-CACCATTGCCCTGACCTGAT-3’. The band sizes obtained were as follows: KI (deleted): 8.6 kb, KI (undeleted): 7.0 kb, and WT: 15.77 kb. Rank^e1+/-^ mice were viable, fertile, and without overt abnormalities. In contrast, Rank^e1-/-^ mice were infertile and exhibited developmental phenotypes similar to other Rank^-/-^ mouse models (82). Rank^e1+/-^ and Rank^e1-/-^ mice were generated in Mendelian ratios (Table S7).

### *In vivo* treatment

CreERT2 activation from Rank^iUbcΔ/Δ^, Rank^iCx3cr1Δ/Δ^ and Rank^iTmem119Δ/Δ^ mice was achieved by intraperitoneal TAM administration (100 mg/kg). Three injections of TAM were administered every other day to mice at 4 weeks of age or in adulthood (10–12 weeks) in both experimental and control groups, and the animals were sacrificed 3 weeks after the last injection. For experiments of microglia depletion, AIN-76A rodent diet (Research Diets,333#D10001; control) and the same with CSF1R antagonist PLX3397334 (MedChemExpress, #D13050910; 600 ppm in chow) were used ad libitum from the corresponding stage (from 4 to 8 weeks).

### Intracranial injection

Mice (12-15 weeks) were anesthetized by intraperitoneal injection of ketamine/xylazine cocktail (ketamine 15 mg/kg BW/xylazine 3 mg/kg BW) and placed in a stereotaxic frame. Four-hydroxytamoxifen (4-OHT) 10 mM was injected in the median eminence (ME) bilaterally using a 32-gauge needle connected to a 1-ml syringe (Neuro-Syringe, Hamilton) and was delivered at a rate of 0.1 μl/min for 7 min (1 μl/injection site) according to the following coordinates: −1.5 mm posterior to the bregma, ±0.2 mm lateral to midline, and −6 mm below the surface of the skull as previously reported (83).

### Odor test

The odor test experiments were conducted in a plastic container from Kaiser+Kraft (40 cm×30cm×23cm). The odorants were administered using a cotton swab, which was impregnated with the relevant odorant and inserted through a small opening located 6 cm above the floor on one of the side walls. A 5-minute adaptation period was initiated, during which the swab was presented without any odorant (water), followed by six consecutive presentations of Odorant A (habituation phase) and six consecutive presentations of Odorant B (dishabituation phase), each lasting 1 minute (84). Olive and sunflower oil were used as A and B odorants, respectively.

### Fertility assay

A male was considered fertile if it had the capacity to impregnate a control female within a maximum period of 90 days, resulting in pregnancy and successful birth of pups. A female was considered fertile if she could be impregnated by a control male within the same period. A male or female was considered subfertile if more time was required to become pregnant or to impregnate compared to control mice.

### Pubertal onset and estrous cycle

To assess pubertal maturation phenotypically, we monitored the day of balanopreputial separation in males and vaginal opening in females, both established external markers of puberty onset. In females, the day of first estrous cycle was determined through daily vaginal cytology beginning from the day of vaginal opening. Vaginal smears were collected and spread onto air-dried glass slides. The slides were then examined under a light microscope to identify the stage of the estrous cycle, based on the presence of cell types characteristic of each phase (85).

### Evaluation of gonadal maturation

Gonadal maturation was assessed by morphometric analyses of H&E staining of a representative slide of testes and ovaries (86, 87). Assessment of the presence of corpora lutea was conducted as index of ovulation. In the case of the testicular maturation, analyses of the area of circular seminiferous tubules were used as an indicator of spermatogenesis progression.

### Cholesterol analysis in serum

Cholesterol levels were measured in mouse serum according to the manufacturer’s instructions in Pentra C200 Clinical Chemistry Analyzer (Cholesterol CP ABX Pentra, A11A01634).

### Enzyme-linked immunosorbent assay (ELISA) of serum samples

Testosterone (KGE010, R&D Systems) and estradiol (KGE014, R&D Systems) amounts were measured in mouse serum according to the manufacturer’s instructions.

### Whole mount staining

For whole mount analyses, inguinal mammary glands were collected at the time specified, fixed and stained with carmine dye (88).

### Cleared fat pad transplantation assay

Mammary epithelial cells were isolated from mouse mammary glands as previously described (89). Cells isolated from mammary glands were diluted 1:1 with Matrigel Matrix (BD Biosciences, San Diego, CA, for a final volume of 20-30 μL and injected in cleared mammary fat pad of 3-4 weeks old C57BL/6 mice. After 8 weeks, the transplanted fat pads were whole-mounted and carmine stained.

### Pharmacological studies in response to GnRH and kisspeptin treatment

For hormonal LH assays, blood samples were obtained from mouse tail at the designated times after intraperitoneal injection as reported in (90). We analyzed time-course LH responses to intraperitoneal injection of GnRH (0.25 nmol/animal) and Kisspeptin-10 (7.5 nmol/animal). Animals were allowed to recover for 72 hours between tests. For each sample, 4 μL of whole blood was diluted 46 μL of 0.1 M phosphate-buffered saline (PBS) with 0.05% Tween 20, snap-frozen on dry ice, and stored at -80ºC. Mice were handled (5-10 min) every week before 3wk before blood sampling, to habituate them for tail-tip bleeding.

### LH pulsatility measurements

Male mice were habituated by daily handling prior to testing. Blood samples (4 µL) were collected from the tip of the tail at 6-min intervals over a 3-h period (9:00–12:00). Samples were diluted in 46 µL of 1× PBS-T (0.05%), immediately frozen, and stored at −80 °C. LH levels were determined using a sensitive LH ELISA, and LH pulses were identified using the PULSAR (91).

### Highly sensitive assay of LH levels in blood

LH amounts in blood were measured using a highly sensitive enzyme linked immune sorbent assay (ELISA) as reported in (92). We use 50 μL of capture antibody diluted 1:1000 (Bovine LHβ 518B7 monoclonal Ab obtained from Lillian E Sibley @ UC Davis) and 50 μL of biotinylated detection antibody (Mouse Monoclonal LH antibody (Medix, 5303 SPRN-5) at 1:2000 in blocking Buffer 5% SMP-PBS-T (0.05% Tween-20). In addition, each well was incubated with 50 μL of Poly-HRP Streptavidin (Thermo Fisher, Cat# N200) and 100μl of OPD (o-Phenylenediamine) (1 Tablet-20 mg of OPD in 48ml of Citrate buffer +20μl of 30% H2O2; Sigma Aldrich).

### Microglia cell line and primary mouse microglia culture

The microglia cell line BV2 (ABC-TC212S, Accgene) was grown in DMEM medium with 10% fetal bovine serum (Gibco) and 100 UI/ml penicillin and 100 µg/ml streptomycin (Gibco) in a water-saturated atmosphere of 5% CO2 and 5% air. Microglia primary culture was obtained from a mixed astromicroglial primary culture from newborns C57BL/6 mice of both genders (93). The day before the procedure, 6-well plates were coated with 10% of Poly-D-Lysine and incubated overnight at 37°C. Brains from neonatal mice (P0-P5, preferably P3) were carefully extracted under a dissection microscope, ensuring the removal of meninges to prevent contamination with perivascular macrophages. Brains were stored in F50 Hibernate solution until trypsinization. Brain tissues were mechanically dissociated using scissors and pipetting and chemically dissociated with trypsin for 7 minutes at 37°C in a thermomixer. Following digestion, the brain tissue was further homogenized by pipetting. The dissociated tissue was inactivated using Micro Full Medium (DMEM-F12 (1:1), 10% heat-inactivated FBS, 1% MEM NEAA, gentamicin (10 µg/ml), 100 UI/ml penicillin and 100 µg/ml streptomycin, filtered through a 40 µm nylon filter, and seeded into Poly-D-Lysine-treated wells (previously washed twice with water and left to dry). One day after seeding, 1 mL of fresh Micro Full Medium was added to the wells without washing, allowing cells to adhere. Media changes were performed every 2–4 days without washing the cultures. Approximately 20 days after seeding, a gentle trypsinization (0.25% trypsin with 1 mM EDTA) was performed to remove astrocytes from the culture. The cultures were incubated at 37°C for 20–45 minutes, until separation occurred. Trypsin was then inactivated with Micro Full Medium. Subsequently, astrocyte-conditioned medium (collected from the astro-microglial plates and diluted 1:1 with Micro Full) was added, and cultures were incubated for 24 hours. Then, cells were treated with Rankl (100 ng/mL) for 6 hours. The cell pellet was then collected for RNA extraction.

### Tissue histology and immunostaining in paraffin blocks

Mouse tissue samples (mammary gland, bone, testes and ovaries) were fixed in formalin overnight at 4ºC and embedded in paraffin according to the CNIO Histopathology Unit protocols. Bone samples were decalcified by the CNIO Histopathology Unit prior to embedding. Five-μm sections were cut for histological analyses processed for H&E staining or immunostaining. Monoclonal anti-ERα antibody (non-diluted medium from hybridoma) was generated by CNIO Monoclonal Antibodies Unit and immunohistochemistry of mammary gland was performed by CNIO Histopathology Unit.

### Immunofluorescence staining of brain tissue

Adult mice were anesthetized with 50–100 mg/kg of Ketamine-HCl and 5–10 mg/kg Xylazine-HCl and perfused through the heart with 20 mL of PBS, followed by 50–100 mL of 4% PFA (pH 7.4). Brains were collected and fixed in the same fixative for 24 hours at 4°C, cryoprotected in 30% sucrose, embedded in optical cutting temperature (OCT) embedding medium (Tissue-Tek), frozen on dry ice, and stored at −80°C until use. Tissues were cryosectioned (Leica cryostat) between 45–80 μm. Coronal sections were washed in PBS and incubated for 60 minutes in blocking solution (0.3% BSA + 0.3% Triton X-100 in 1× PBS for PoA, or 0.3% Triton X-100, 10% normal donkey serum, and 1% BSA for mediobasal hypothalamus). Sections were then incubated overnight with primary antibodies diluted in the respective blocking solution: rabbit anti-GnRH (1:1000, 269501-AP, Proteintech), goat anti-Iba1 (1:500, AB5076, Abcam), chicken anti-Iba1 (1:1000, 234009, Synaptic Systems), and rat anti-CD68 (1:500, MCA1957GA, Bio-Rad). TdTomato fluorescence was detected by endogenous signal. After primary antibody incubation, sections were rinsed three times in PBS and incubated for 120 minutes at room temperature with fluorochrome-conjugated secondary antibodies (1:1000, Jackson ImmunoResearch). Finally, sections were washed, mounted with Permount containing DAPI, and coverslippe. For quantification of GnRH neuron population in the PoA, images were acquired using a Zeiss Axio Imager Z2 ApoTome microscope (Zeiss, Germany), as previously reported (94). The quantification was performed on one-third of the mouse brain and then normalized to account for the total GnRH population To analyze the number and morphology of microglia in the mediobasal hypothalamus, 3D confocal images were acquired using the z-stack function on an LSM 700 confocal microscope with a 40× oil-immersion objective (Zeiss, Oberkochen, Germany), a Leica Stellaris 8 microscope with 20×/63× glycerol-immersion objectives, or a Leica TCS SP8 confocal microscope with a 40× oil-immersion objective. Maximal Z-projections were generated using the same number of planes for each staining. Three-dimensional reconstruction was performed with Bitplane Imaris 9 software (Bitplane, Zurich, Switzerland) and Imaris x64 9.6.0 image analysis software (Oxford Instruments, Concord, MA). Images were first subjected to background subtraction and then processed using the surface and filament modules to reconstruct microglia, microglial cell bodies, microglial processes, GnRH, and CD68. Although most of the analysis was automated by the software, the origin of each process was independently verified to ensure correct cell assignment, and any erroneous connections were manually removed.

### Immunohistochemistry (IHC) of NKB

NKB expression was evaluated following validated protocols for IHC with modifications (95). Fixed Brains were cut (30 μm) and divided in four sets of coronal sections in a freezing microtome (Leica CM1850 UV). For IHC analyses, one set of sections encompassing the mediobasal hypothalamus was used. This set of free-floating sections was mounted in Super-Frost Plus slides (Thermo Fisher Scientific, Inc.), air dried and fixed with 4% Formalin solution for 15 minutes (Sigma-Aldrich, St Louis, MO, USA), washed with Tris-buffered saline (TBS; pH 7.6) for 5 minutes and finally dehydrated with increasing concentrations of ethanol. Endogenous peroxidases were blocked by incubation with H_2_O_2_-methanol solution for 10 min. Subsequently, slides were washed in TBS and incubated for 72 hours at 4 ºC with rabbit anti-pro-NKB (IS-39, dilution1:10000 kindly donated by Dr. P. Ciofi) in incubation buffer composed of 2% donkey serum and 0.3% Triton X-100 in TBS. Slides were then washed in TBS and incubated with a secondary biotinylated donkey anti-rabbit 1:500 (Ref 711-066-152, Jackson Immunoresearch) at RT for 1.5 hours. Then, sections were incubated tissue in A/B Vectastain Elite solution (Vectastain Elite ABC Kit reagents; Vector Laboratories, Burlingame, CA, USA) for 1.5 hours. Next, sections were washed in TBS and 0.1 M Acetate buffer, incubated with glucose oxidase and diaminobenzidine-nickel (DAB/Ni) for 20 min at RT, and washed in 0.1 M acetate buffer and TBS buffer. Finally, brain sections were dehydrated with ascending concentrations of alcohol and xylene, and cover-slipped using Eukitt mounting medium (MICROPTIC SL, Barcelona). Immunoreactivity was visualized using a Leica microscope (DM2500) with a 10X lens. Densitometric analyses were performed using ImageJ (https://imagej.net/ImageJ, NIH).

### iDISCO (immunolabeling-enabled three-dimensional imaging of solvent-cleared organs)

Briefly, tissues were fixed in 4% PFA by perfusion and stored in PBS. Samples were dehydrated in a methanol/PBS gradient (20%, 40%, 60%, 80%, and 100% twice, 1 hour each) and then delipidated with dichloromethane (2:1 dichloromethane/methanol) overnight at 4 °C. The following day, samples were bleached in a hydrogen peroxide/methanol solution (1:6 H_2_O_2_ to 5:6 methanol) overnight to reduce autofluorescence. On the third day, the samples were hydrated in a methanol/PBS gradient (100%, 80%, 60%, 40%, 20%, PBS; 1 hour each). After rehydration, samples were permeabilized and blocked in PBSGT (PBS, 0.2% gelatin, 1% Triton X-100, 0.05% sodium azide) for 4 days and then incubated with GnRH antibody (Proteintech, 26950-1-AP) for 14 days. Subsequently, samples were washed six times for 1 hour each in PBS + 1% Triton X-100 and then incubated with Alexa Fluor 555 anti-rabbit secondary antibody (Life technologies, SA) in PBSGT for 1 week. After secondary antibody incubation, samples were washed six times for 1 hour each in PBS + 1% Triton X-100. All immunolabeling steps were performed with gentle rotation and protection from light. For tissue clearing, samples were dehydrated again in a methanol/PBS gradient (20%, 40%, 60%, 80%, and 100% twice, 1 hour each) and then delipidated with dichloromethane (2:1 dichloromethane/methanol) overnight at 4 °C. The following day, samples were immersed in dibenzyl ether (DBE) to achieve optical transparency. Cleared tissues were stored in fresh DBE at room temperature, protected from light, and imaged as whole mounts. Three-dimensional imaging was obtained as reported(94). Imaging was performed using an Ultramicroscope I (LaVision BioTec) controlled with ImspectorPro software (LaVision BioTec). The light sheet was created with a laser in combination with two cylindrical lenses. A binocular stereomicroscope (MXV10, Olympus) equipped with a 2× objective (MVPLAPO, Olympus) allowed imaging at multiple magnifications (1.6×, 4×, 5×, and 6.3×). Samples were placed in a 100% quartz imaging chamber (LaVision BioTec) filled with DBE and illuminated laterally using the full width of the laser sheet. Image acquisition was performed with a PCO Edge SCMOS CCD camera (2,560 × 2,160 pixels, LaVision BioTec) with a z-step size of 2 μm. The resulting TIFF image stacks were converted to Imaris-compatible files (Imaris FileConverter, Bitplane) for three-dimensional reconstruction and imported into Imaris (Bitplane) for visualization and generation of snapshots.

### RNAscope detection of Kiss1

RNAscope® Multiplex v.2 (ACDbio) in situ hybridization was employed for the detection of Kiss1 mRNA in Kiss1 neurons from the hypothalamic arcuate (ARC) nucleus. Whole brains from male mice (n=4/group) were frozen and stored. Brain coronal sections of 16 µm were cut and distributed into five sets of slides containing the main hypothalamic area where Kiss1 neurons are located (ARC). Four representative brain sections per group were selected for RNAscope assay. RNAscope Multiplex Fluorescent Reagent Kit v2 Assay (Advanced Cell Diagnostics) was used, with the following RNA catalog probe: Rn-Kiss1-C3 probe (Cat No. 503421-C3) of *Rattus norvegicus* Kiss1 mRNA (NM_181692.1). Following the manufacturer’s protocol, ARC-containing brain sections were fixed in 4 % paraformaldehyde at 4 ºC for 30 min, then dehydrated in increasing concentrations of ethanol and a hydrophobic barrier was created around the tissue. H2O2 was added and incubated for 10 min to block the endogenous peroxidase activity. The tissue was then incubated with Protease III for 30 min. The target probe hybridized to ARC sections for 2 h at 40 ºC, the slides were washed twice with 1X wash buffer and immersed in 5X sodium citrate saline overnight at 4 ºC. Amplification steps were performed according to the kit protocol and specific fluorophore was used to detect the probe: Cyanine 5 for Rn-Kiss1-C3 probe. Finally, slides were cover-slipped using Fluoroshield with DAPI (Sigma-Aldrich) mounting medium. In situ hybridization was visualized on a Leica Thunder microscope at the Unit of Microscopy of IMIBIC. Quantification was performed counting the total number of cells bilaterally.

### RNA isolation, RT-PCR and gene expression analysis

Total RNA was isolated from mouse tissue using Maxwell® RSC simplyRNA Tissue Kit (AS1340 Promega). One thousand ng (testis/ovary/cell lines) or 500 ng (pituitary gland/hypothalamus) of RNA was reverse transcribed into cDNA using 200 U Superscript II plus random hexamer oligos (Invitrogen). Different genes were amplified with LightCycler® 480 Probes Master (Roche, 04707494001) and a LightCycler® 480 thermocycler (Roche) and normalized relative to the *Hprt1* or *Gapdh* mouse keeper gene. Primers sequences are indicated in Table S7.

### Public scRNAseq analyses

Analyses of gene expression in mouse hypothalamus (20), were conducted using the interactive CellxGene viewer (https://www.mrl.ims.cam.ac.uk), while analyses of gene expression in human hypothalamus were performed using R/Seurat (21) or CellxGene viewer (22).

### Metagene analyses and correlations

Metagenes and correlation analyses provided in the manuscript were generated by GEPIA2 (96). Pearson’s correlation coefficient was used as the statistical test for the correlation analyses. The microglial gene signature was extracted from PanglaoDB (97), a publicly available single-cell RNA sequencing database that provides well-characterized, cell-type specific gene signatures and is widely recommended for cell and tissue identification in scRNA-seq studies.

### scRNAseq of mouse hypothalamus

Single-cell suspensions were prepared from the hypothalamus of Rank^iUbcΔ/Δ^ mice enriched in microglia. These mice were treated with TAM (100 mg/kg) at puberty onset and analyzed four weeks after the first treatment. The weight of the testes was measured as an indicator of efficient Rank depletion. Hypothalamic tissue from 5 control and 5 Rank^iUbcΔ/Δ^ mice was pooled on ice. The tissue was then mechanically and enzymatically digested using DMEM/F12 (Gibco), 0.3% collagenase A (Sigma), 2.5 U/ml dispase (Sigma), 2 mM HEPES, and Penicillin/streptomycin (ThermoFisher Scientific) for 30 minutes at 37 ºC, with the digestion buffer changed every 10 minutes. After digestion, the cells were filtered through a 70 μm filter and centrifuged at 300 g for 5 minutes at 4 ºC. Erythrocytes were removed using ACK buffer, followed by using 40% Percoll to eliminate myelin debris. Subsequently, single cells from the hypothalamus were sorted to remove debris and select live cells for further analysis. Cell sample was loaded onto a 10x Chromium Single Cell controller chip B (10x Genomics) as described in the manufacturer’s protocol (PN-1000121, Chromium Next GEM Single Cell 3’ GEM, Library & Gel Bead Kit v3.1). Libraries were sequenced on the Illumina NextSeq 550 platform (with v2.5 reagent kits) with paired-end sequencing (28 bp + 56 bp bases).

### Data processing, normalization, and clustering annotations

Bollito (98) pipeline was employed to perform initial steps of the analysis as follows: sequencing quality was checked with FastQC (http://www.bioinformatics.babraham.ac.uk/projects/fastqc/); reads were aligned to the mouse reference genome (GRC39m from GENCODE (99)) with STARsolo (STAR v2.7.1) (100); Seurat v3.2.3 (101) software was used to check the quality of sequenced cells, and perform data normalization, dimensionality reduction and clustering. Cells expressing less than 200 features and more than 2x the median of features were filtered out. Moreover, a maximum cutoff of 10% and 40% were set for mitochondrial and ribosomal percentage, respectively. Mitochondrial percentage and merge effect were regressed out in order to minimize their effect on the samples. To perform the dimensionality reduction, the top 2500 variable genes are considered, and PCs and number of neighbors are both set to 20. The microglial subpopulation was subsetted for further clustering using the same approach. Annotation of the resulting clusters was first performed using singleR v2.0.0 (102) according to data provided by previous public brain datasets (26, 27) and afterwards this annotation was manually curated based on marker genes extracted from recent literature regarding hypothalamic cell subpopulations (20, 25).

### Differential gene expression analysis

Differential gene expression analysis (DEA) was performed using Seurat through the default Wilcoxon test, and significance is defined by a 0.05 adjusted P value threshold and an absolute log2-fold change (log2FC) > 0.25. In addition, default minimal threshold of 10% of expression in (at least) one of the compared populations is conserved.

### Gene Set Enrichment Analysis

Gene Set Enrichment Analysis (GSEA) was used to interpret gene expression data through GSEA Preranked (103) on a preranked gene list sorted according to log2FC resulting from previous DEA. Those gene sets with significant enrichment levels (FDR q-value < 0.25 or 0.05) were considered. 1.000 permutations are performed and a minimal and maximum number of genes per reference were defined as 10 and 500 respectively.

### Statistical analysis and reproducibility

Statistical analyses were performed using GraphPad Prism software version 8. Data are represented as the mean ± S.E.M. The measurements were taken from different mice, avoiding technical replicates. When comparisons were made between two experimental groups, an unpaired, two-tailed Student’s t-test was used. When comparing multiple variables between two experimental groups, an analysis of variance (two-way ANOVA) was employed, followed by post hoc tests for multiple comparisons (Tukey). For the analysis of pubertal onset, a Gehan–Breslow–Wilcoxon matched-pairs test was applied.

## Supplementary Material

Fig. S1

## Figures and Tables

**Table 1:** Clinical and genetic characteristics of CHH probands harboring rare RANK gene variants. Tier I (highlighted in grey) includes rare sequence variants predicted to be deleterious, with CADD scores > 20 and AlphaMissense scores > 0.5, indicating stronger pathogenic potential. Abbreviations: CADD, Combined Annotation Dependent Depletion; MAF, maximum minor allele frequency in gnomAD; HH, hypogonadotropic hypogonadism; KS, Kallmann syndrome (CHH with anosmia or hyposmia); M, male; F, female; /, absent.

Predicted pathogenicity	Proband	Sex	Diagnosis	*RANK* mutation	dbSNP 10	MAF	CADO	Alpha missense	Inheritance	Mutations in known CHH genes
Tier 1: Likely pathogenic	A	M	CHH reversal	C.1145A>G [p.E382G]	rs200791079	0.00003422	29.2	0.6398	No information	/
	B	M	KS	C.718A>G [p.K240E]	rs148185533	0.0007522	27.7	0.5288	Familial	/
Tier II: Uncertain or likely benign	C	M	CHH	C.323C>T [p.T1081]	rs376617343	0.00000195	19.8	0.113	Sporadic	/
	E	M	KS	C.932C>T [p.T311l]	rs764561352	0.00002074	5.328	0.0945	Familial	/
	F	F	KS reversal	C.543A>T [p.R181S]	rs762733251	0.00002474	0.141	0.1393	Familial	/
	G	M	KS	C.766A>T [p.S256C]	.	0	0.217	0.113	No information	/

## Data Availability

The scRNAseq results obtained in this work have been deposited at GEO, GSE240379. Mouse strains used in this study are subject to a Material Transfer Agreement and can be shared upon reasonable request and in accordance with the corresponding institutional regulations. The rest of the data used in the preparation of the manuscript are available in the main text or in the supplementary material file.
