# Supplementary material for: Microglia Rank signaling regulates GnRH neuronal function and the Hypothalamic-Pituitary-Gonadal axis
Source: Science. Author manuscript; Available in PMC 2026 May 7. (PMC7619046; doi:10.1126/science.aeb6999)
Supplement: Fig. S1 [file EMS213420-supplement-Fig__S1.pdf]

Science
AAAS

Supplementary Materials for

Microglia Rank signaling regulates GnRH neuronal function and the hypothalamic-pituitary-gonadal axis

A. Collado-Sole *et al.*

Corresponding author(s):
Eva González Suárez, egonzalez@cnio.es; Alejandro Collado-Sole, acollado@cnio.es

DOI: 10.1126/science.aeb6999

The PDF file includes:

Figs. S1 to S15
Tables S1 to S7

Other Supplementary Material for this manuscript includes the following:

Reproducibility Checklist
Movies S1 to S4

Figure S1

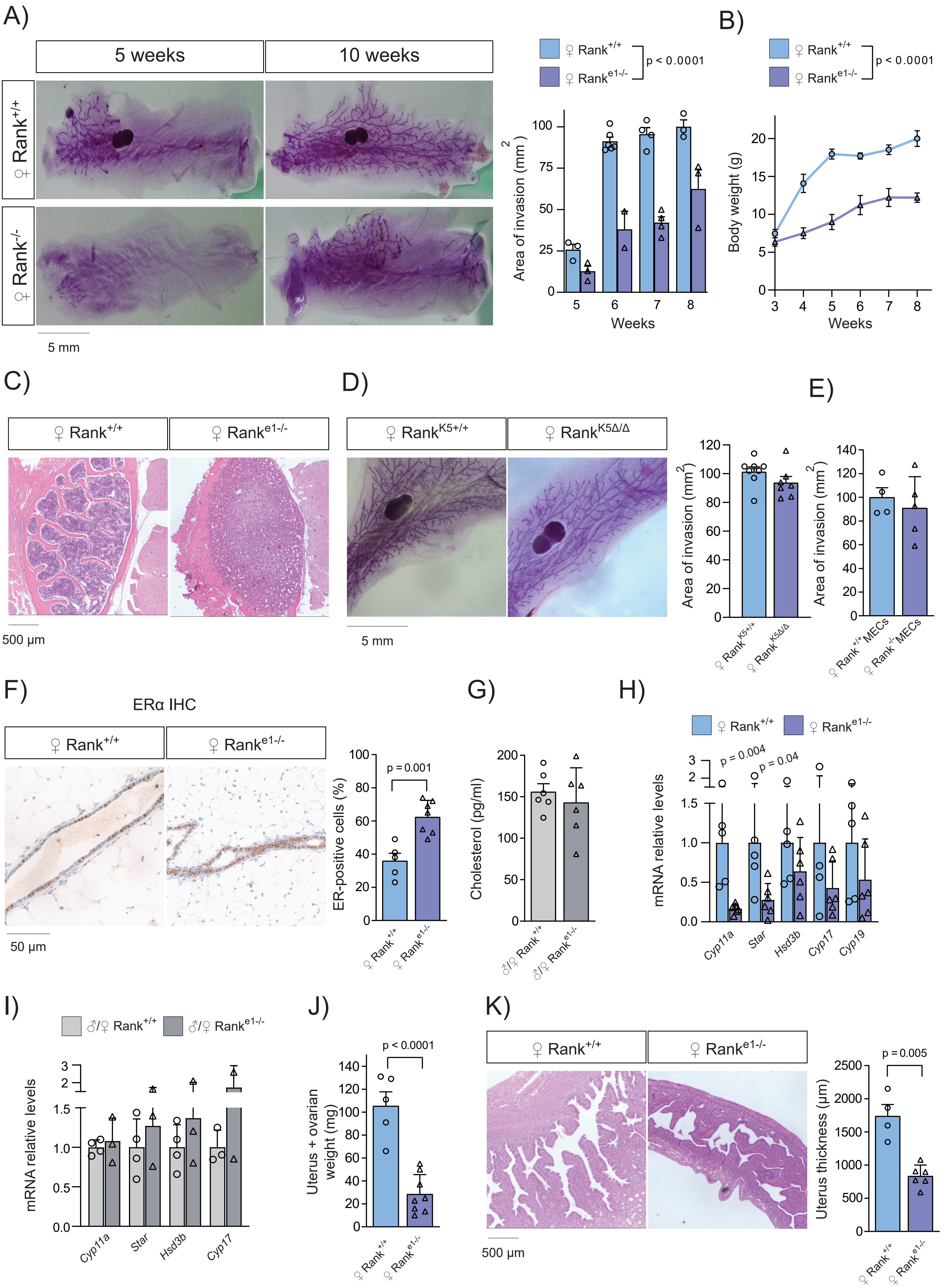

A)
5 weeks
10 weeks
♀ Rank+/+
♀ Rank-/-
5 mm
♀ Rank+/+
♀ Ranke1-/-
p < 0.0001
Area of invasion (mm2)
Weeks
B)
Body weight (g)
Weeks
C)
♀ Rank+/+
♀ Ranke1-/-
500 µm
D)
♀ RankK5+/+
♀ RankK5Δ/Δ
5 mm
E)
Area of invasion (mm2)
♀ Rank+/+ MECs
♀ Rank-/- MECs
F)
ERα IHC
♀ Rank+/+
♀ Ranke1-/-
50 µm
ER-positive cells (%)
p = 0.001
G)
Cholesterol (pg/ml)
♂/♀ Rank+/+
♂/♀ Ranke1-/-
H)
mRNA relative levels
p = 0.004
p = 0.04
Cyp11a
Star
Hsd3b
Cyp17
Cyp19
I)
♂/♀ Rank+/+
♂/♀ Ranke1-/-
mRNA relative levels
J)
p < 0.0001
Uterus + ovarian weight (mg)
K)
♀ Rank+/+
♀ Ranke1-/-
500 µm
p = 0.005
Uterus thickness (µm)

Fig. S1. Constitutive Rank loss induces defective mammary gland and gonadal development in females. (A) Representative images of whole mounts and quantification of invasion in mammary glands from wild-type (Rank$^{+/+}$) and constitutive whole-body Rank-deficient (Rank$^{-/-}$) mice at indicated ages. (B) Body weight of Rank$^{+/+}$ (n = 6) and Rank$^{e1-/-}$ (n = 8) females at indicated ages. (C) Representative images of H&E staining of vertebral bone from Rank$^{+/+}$ and Rank$^{e1-/-}$ female mice. (D) Representative whole mounts and quantification of the invaded area in mammary glands in Rank$^{K5+/+}$ and Rank$^{K5\Delta/\Delta}$ mice. (E) Quantification of the invaded area in mammary glands regenerated by transplantation of mammary tissue pieces from Rank$^{+/+}$ or Rank$^{-/-}$ mice into wild-type hosts. (F) Representative images of ERα expression and quantification relative to mammary epithelial cells in Rank$^{+/+}$ and Rank$^{e1-/-}$ mammary glands. (G) Cholesterol concentration in serum from control and Rank$^{e1-/-}$ mice in both sexes. (H) mRNA amounts of indicated steroidogenic genes in Rank$^{e1-/-}$ ovaries relative to the control littermates. (I) mRNA amounts of indicated steroidogenic genes in Rank$^{e1-/-}$ adrenal glands relative to the control littermates of both sexes. J) Uterus plus ovarian weight in Rank$^{e1-/-}$ and corresponding control females. K) Representative H&E staining of uteri from Rank$^{+/+}$ and Rank$^{e1-/-}$ females and quantification of the thickness of the uteri. Analyses were performed in 8-weeks old mice (C-K). Data are represented as mean +/-SEM with each dot representing a mouse. P values were calculated using two-way ANOVA followed by Tukey's multiple comparisons (A-B) or two-tailed unpaired t-test (D-K) and exact P values are indicated when statistically significant.

Figure S2

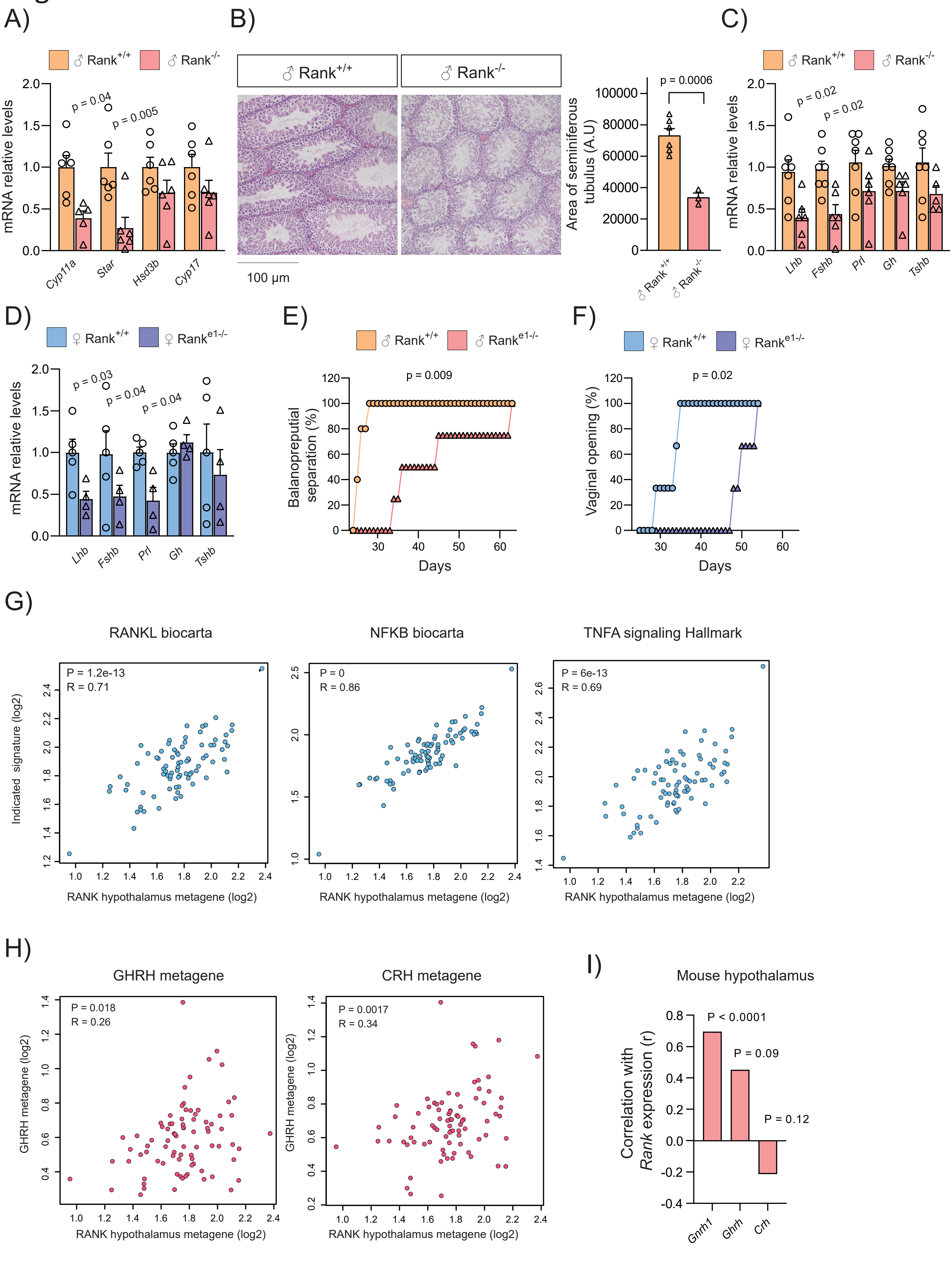

A)
♂ Rank+/+ ♂ Rank-/-
mRNA relative levels
p = 0.04
p = 0.005
Cyp11a Star Hsd3b Cyp17
B)
♂ Rank+/+
♂ Rank-/-
100 µm
Area of seminiferous tubulus (A.U)
p = 0.0006
C)
♂ Rank+/+ ♂ Rank-/-
mRNA relative levels
p = 0.02
p = 0.02
Lhb Fshb Prl Gh Tshb
D)
♀ Rank+/+ ♀ Ranke1-/-
mRNA relative levels
p = 0.03
p = 0.04
p = 0.04
Lhb Fshb Prl Gh Tshb
E)
♂ Rank+/+ ♂ Ranke1-/-
Balanopreputial separation (%)
p = 0.009
Days
F)
♀ Rank+/+ ♀ Ranke1-/-
Vaginal opening (%)
p = 0.02
Days
G)
RANKL biocarta
P = 1.2e-13
R = 0.71
NFKB biocarta
P = 0
R = 0.86
TNFA signaling Hallmark
P = 6e-13
R = 0.69
Indicated signature (log2)
RANK hypothalamus metagene (log2)
H)
GHRH metagene
P = 0.018
R = 0.26
CRH metagene
P = 0.0017
R = 0.34
GHRH metagene (log2)
RANK hypothalamus metagene (log2)
I)
Mouse hypothalamus
Correlation with Rank expression (r)
P < 0.0001
P = 0.09
P = 0.12
Gnrh1 Ghrh Crh

Fig. S2. Constitutive Rank loss impairs the establishment of the HPG axis. (A) mRNA amounts of indicated steroidogenic genes in $Rank^{-/-}$ testes relative to corresponding $Rank^{+/+}$ littermates. (B) Representative images of H&E staining of $Rank^{+/+}$ and $Rank^{-/-}$ testes and quantification of the area of their seminiferous tubules. (C) mRNA amounts of *Lhb*, *Fshb, Prl*, *Gh* and *Tshb* in pituitary glands from $Rank^{-/-}$ males relative to control littermates. (D) mRNA amounts of *Lhb*, *Fshb, Prl*, *Gh* and *Tshb* in pituitary glands from $Rank^{ex1-/-}$ females relative to control littermates. (E) Graphs represent the cumulative percentage of subjects that achieved balanopreputial separation state. Statistical differences were tested using a Gehan-Breslow-Wilcoxon matched-pairs test ($Rank^{+/+}$ vs. $Rank^{ex1-/-}$ mice, $\chi^2 = 5.24$, P = 0.02, n = 5 and n=3). (F) Graphs represent the cumulative percentage of animals that achieved vaginal opening state. Statistical differences were tested using a Gehan-Breslow-Wilcoxon matched-pairs test ($Rank^{+/+}$ vs. $Rank^{ex1-/-}$ mice, $\chi^2 = 5.05$, P = 0.02, n = 3 and n = 3). (G) Correlation between RANK metagene and public RANK/NFKB signatures (RANKL biocarta, NFKB biocarta and TNFA signaling from Hallmark, Table S1) analyzed in human hypothalamus (GTEx). The correlation index was calculated using Pearson correlation. (H) Correlation between RANK metagene and GHRH/CRH metagenes analyzed in the human hypothalamus (GTEx). The correlation index was calculated using Pearson correlation. (I) Correlation between *Rank* expression and specific neuropeptides (*Gnrh1* (n = 28), *Ghrh* (n = 15), and *Crh* (n = 13)) in the mouse hypothalamus from wild-type mice (8-10 weeks). The correlation index was calculated using Pearson correlation. Analyses were performed in 8-week-old mice (A-D; I). Data are represented as mean +/-SEM with each dot representing a mouse, P values were calculated using two-tailed unpaired t-test and exact P values are indicated when statistically significant (A-D).

Figure S3

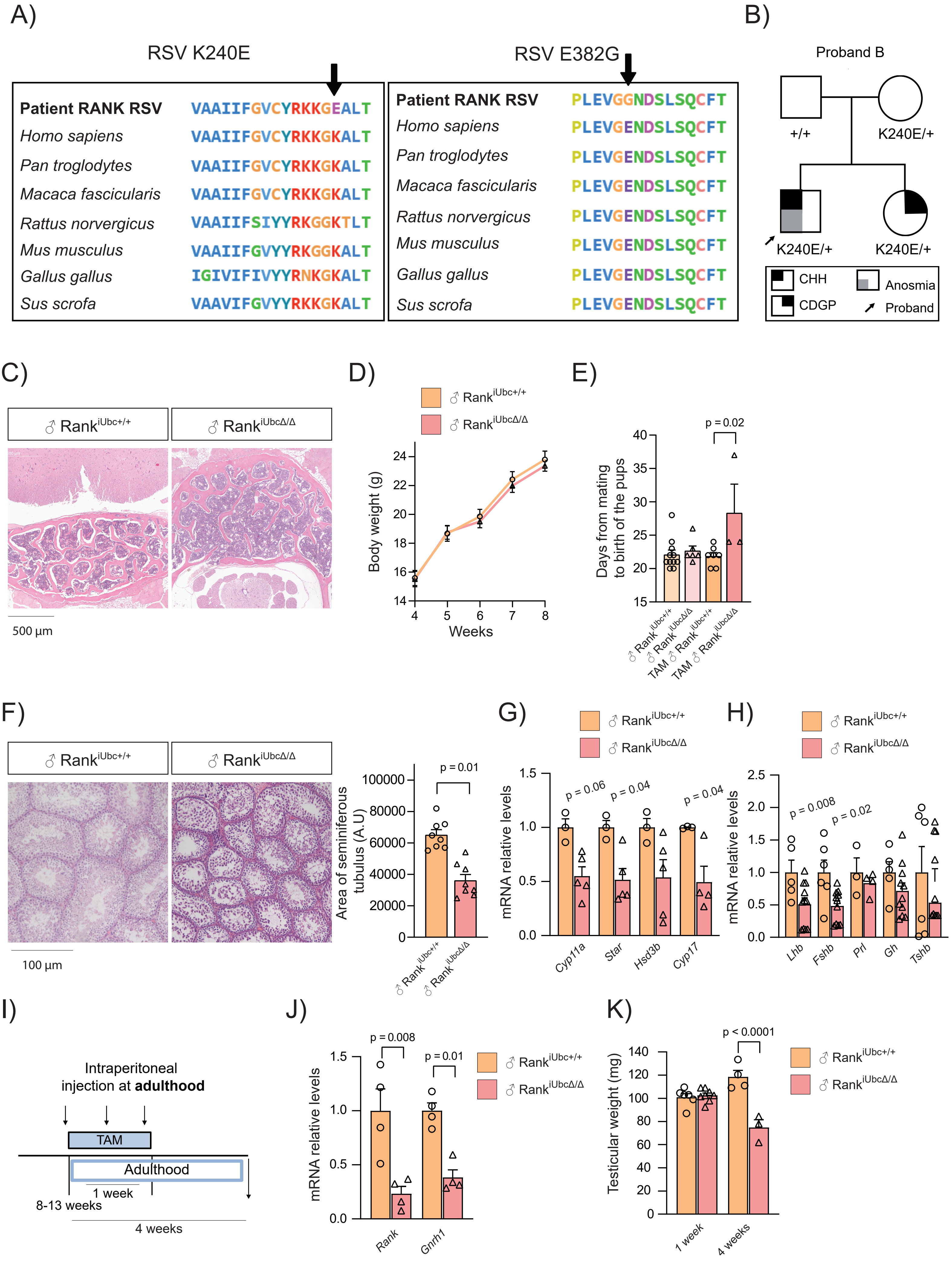

A)
RSV K240E
Patient RANK RSV VAAIIFGVCYRKKGEALT
Homo sapiens VAAIIFGVCYRKKGKALT
Pan troglodytes VAAIIFGVCYRKKGKALT
Macaca fascicularis VAAIIFGVCYRKKGKALT
Rattus norvergicus VAAIIFSIYYRKGGKTLT
Mus musculus VAAIIFGVYYRKGGKALT
Gallus gallus IGIVIFIVYYRNKGKALT
Sus scrofa VAAVIFGVYYRKKGKALT
RSV E382G
Patient RANK RSV PLEVGGNDSLSQCFT
Homo sapiens PLEVGENDSLSQCFT
Pan troglodytes PLEVGENDSLSQCFT
Macaca fascicularis PLEVGENDSLSQCFT
Rattus norvergicus PLEVGENDSLSQCFT
Mus musculus PLEVGENDSLSQCFT
Gallus gallus PLEVGENDSLSQCFT
Sus scrofa PLEVGENDSLSQCFT
B)
Proband B
+/+
K240E/+
K240E/+
K240E/+
CHH
Anosmia
CDGP
Proband
C)
♂ Rank iUbc+/+
♂ Rank iUbcΔ/Δ
500 µm
D)
♂ Rank iUbc+/+
♂ Rank iUbcΔ/Δ
Body weight (g)
Weeks
E)
p = 0.02
Days from mating to birth of the pups
♂ Rank iUbc+/+
♂ Rank iUbcΔ/Δ
TAM ♂ Rank iUbc+/+
TAM ♂ Rank iUbcΔ/Δ
F)
♂ Rank iUbc+/+
♂ Rank iUbcΔ/Δ
100 µm
p = 0.01
Area of seminiferous tubulus (A.U)
G)
♂ Rank iUbc+/+
♂ Rank iUbcΔ/Δ
mRNA relative levels
p = 0.06
p = 0.04
p = 0.04
Cyp11a
Star
Hsd3b
Cyp17
H)
♂ Rank iUbc+/+
♂ Rank iUbcΔ/Δ
mRNA relative levels
p = 0.008
p = 0.02
Lhb
Fshb
Prl
Gh
Tshb
I)
Intraperitoneal injection at adulthood
TAM
Adulthood
1 week
8-13 weeks
4 weeks
J)
p = 0.008
p = 0.01
mRNA relative levels
♂ Rank iUbc+/+
♂ Rank iUbcΔ/Δ
Rank
Gnrh1
K)
p < 0.0001
Testicular weight (mg)
♂ Rank iUbc+/+
♂ Rank iUbcΔ/Δ
1 week
4 weeks

Fig. S3. Postnatal Rank loss impairs the HPG axis in males. (A) Sequence comparison of the *RANK* gene across species, highlighting the conserved amino acid positions that are altered in p.K240E and p.E382G *RANK* mutation carriers. (B) Pedigree B of proband with CHH, carrying *RANK* (*TNFRSF11A K240E*) rare sequence variant, with affected family members and the specific amino acid changes associated with the RSV. Phenotypes related to CHH were indicated as described in the legend. CDGP = Constitutional Delay of Growth and Puberty. (C) Representative images of H&E staining of vertebral bones from $Rank^{iUbc+/+}$ and $Rank^{iUbc\Delta/\Delta}$ male mice. (D) Body weight of $Rank^{iUbc+/+}$ (n = 20) and $Rank^{iUbc\Delta/\Delta}$ (n = 30) males at indicated age (in weeks) upon pubertal Rank loss. (E) Number of pups born from WT females and male mice of panel 1K. (F) Representative images of H&E staining of $Rank^{iUbc+/+}$ and $Rank^{iUbc\Delta/\Delta}$ testes and quantification of the area of seminiferous tubules upon Rank deletion at puberty. (G) mRNA amounts of indicated steroidogenic genes in $Rank^{iUbc\Delta/\Delta}$ testes relative to the corresponding control littermates. (H) mRNA amounts of *Lhb*, *Fshb, Prl*, *Gh* and *Tshb* in pituitary glands from $Rank^{iUbc\Delta/\Delta}$ males relative to control mice upon Rank deletion at puberty. (I) TAM treatment procedure to induce Rank deletion at adulthood in $Rank^{iUbc\Delta/\Delta}$. (J) *Gnrh1* and *Rank* mRNA expression levels in the hypothalamus from $Rank^{iUbc\Delta/\Delta}$ males and control littermates upon Rank deletion at adulthood, 1 week after TAM treatment. (K) Testicular weight of control and $Rank^{iUbc\Delta/\Delta}$ male mice upon Rank deletion in adulthood (S3i). $Rank^{iUbc\Delta/\Delta}$ and $Rank^{iUbc+/+}$ male mice were TAM treated in puberty (as shown in Fig. 1J) (C-H) or adulthood (as shown in Fig. S1i) (J-K). Data are represented as mean +/-SEM, with each dot representing a mouse; P values were calculated using two-tailed unpaired t-test (E-H; J-K), and exact P values are indicated when statistically significant.

Figure S4

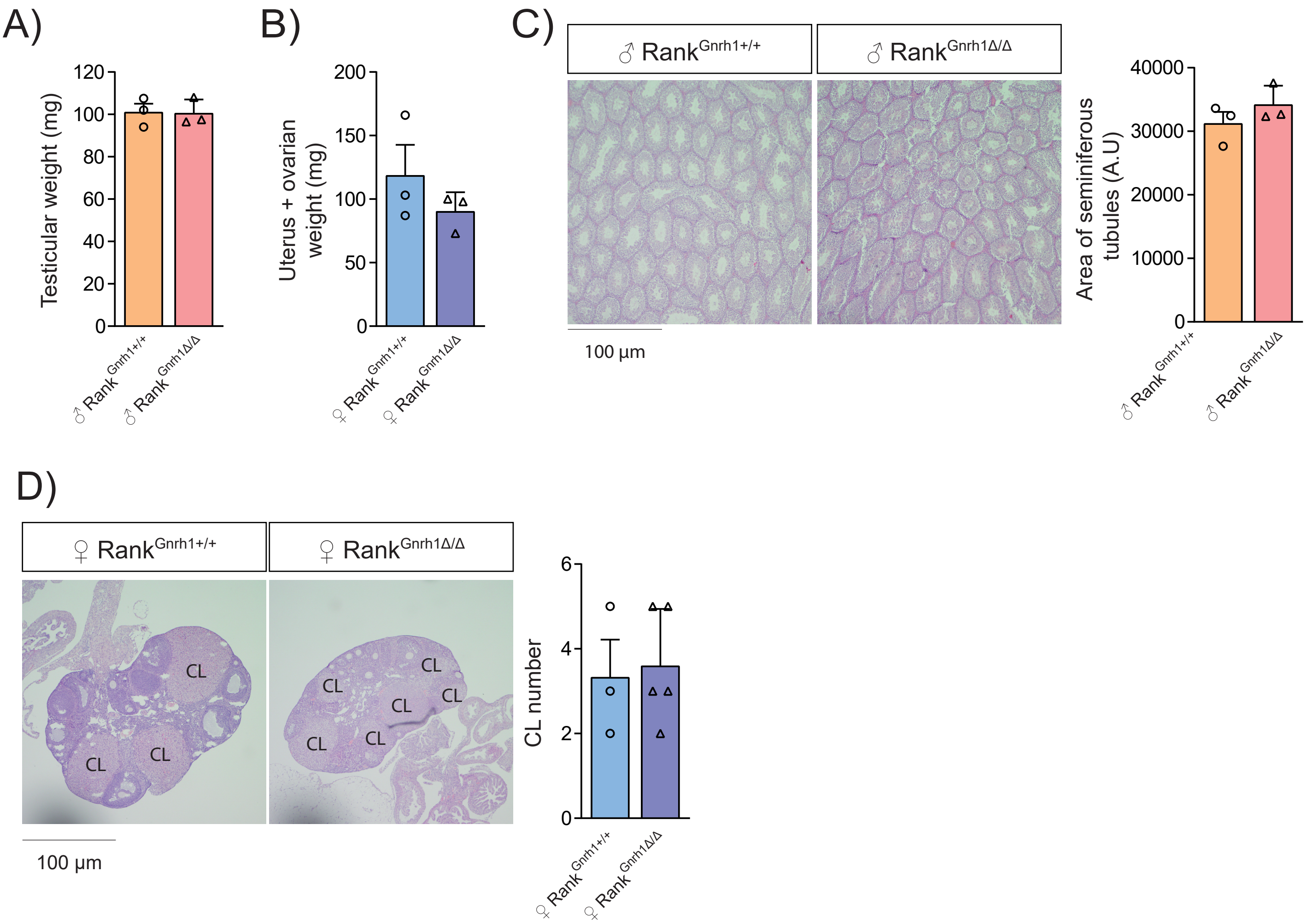
A)
Testicular weight (mg)
120
100
80
60
40
20
0
♂ Rank Gnrh1+/+
♂ Rank Gnrh1Δ/Δ
B)
Uterus + ovarian weight (mg)
200
150
100
50
0
♀ Rank Gnrh1+/+
♀ Rank Gnrh1Δ/Δ
C)
♂ Rank Gnrh1+/+
♂ Rank Gnrh1Δ/Δ
100 µm
Area of seminiferous tubules (A.U)
40000
30000
20000
10000
0
♂ Rank Gnrh1+/+
♂ Rank Gnrh1Δ/Δ
D)
♀ Rank Gnrh1+/+
♀ Rank Gnrh1Δ/Δ
CL
100 µm
CL number
6
4
2
0
♀ Rank Gnrh1+/+
♀ Rank Gnrh1Δ/Δ

Fig. S4. Rank deletion in GnRH neurons does not impair the HPG axis (A) Testicular weight of control and $Rank^{Gnrh1\Delta/\Delta}$ male mice. (B) Uterus plus ovarian weight in $Rank^{Gnrh1\Delta/\Delta}$ and control females. (C) Representative images of H&E staining of $Rank^{Gnrh1\Delta/\Delta}$ and $Rank^{Gnrh1+/+}$ testes and quantification of the area of their seminiferous tubules. (D) Representative images of H&E staining of $Rank^{Gnrh1\Delta/\Delta}$ ovaries and corresponding control. Analyses were performed in 8-week-old mice (A–D). Data are represented as mean +/-SEM, with each dot representing a mouse; P values were calculated using two-tailed unpaired t-test, and exact P values are indicated when statistically significant (A-D).

Figure S5

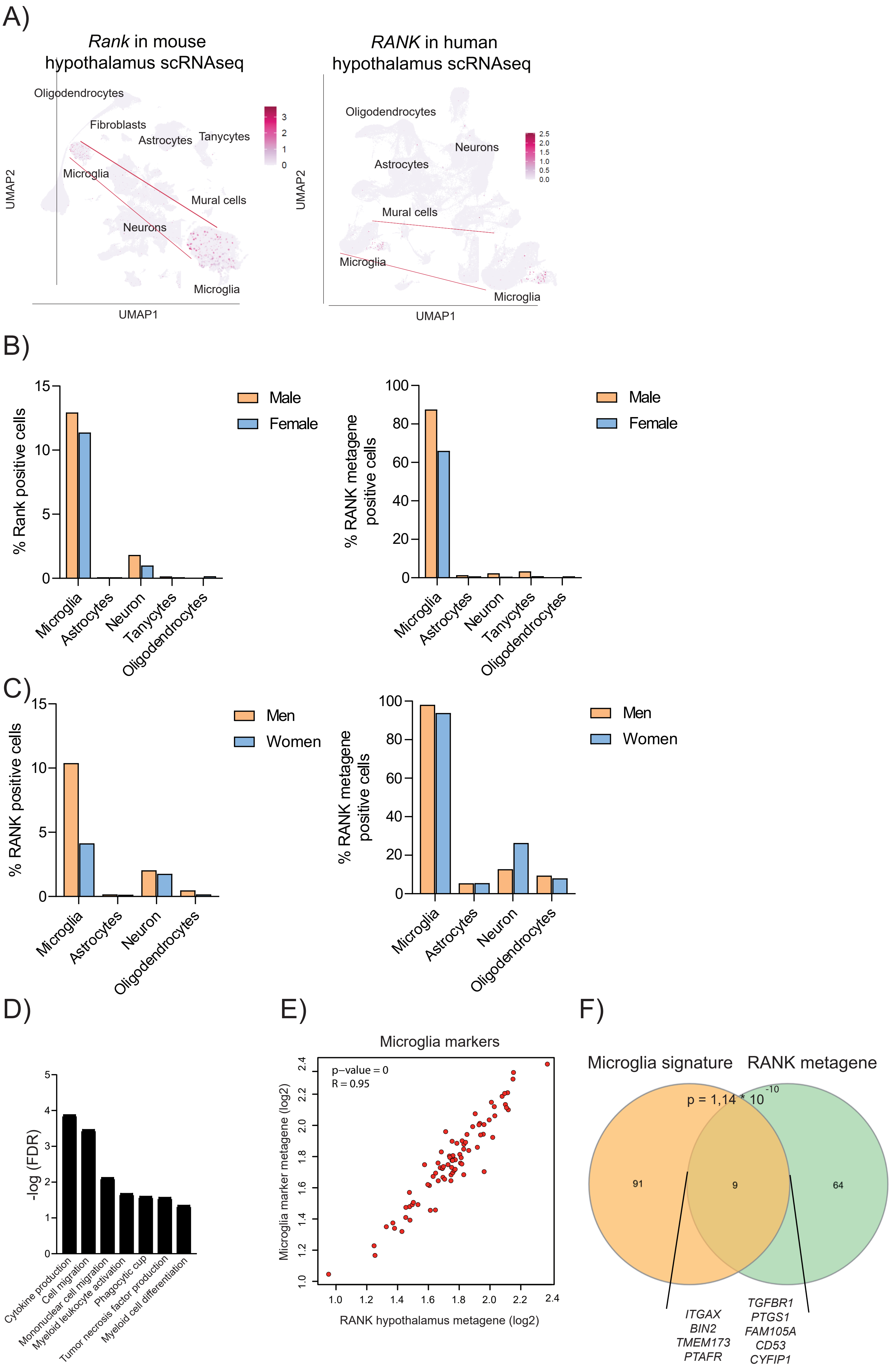

A)
Rank in mouse hypothalamus scRNAseq
RANK in human hypothalamus scRNAseq
Oligodendrocytes
Fibroblasts
Astrocytes
Tanycytes
Microglia
Mural cells
Neurons
UMAP1
UMAP2
B)
% Rank positive cells
% RANK metagene positive cells
Male
Female
Microglia
Astrocytes
Neuron
Tanycytes
Oligodendrocytes
C)
% RANK positive cells
Men
Women
D)
-log (FDR)
Cytokine production
Cell migration
Mononuclear cell migration
Myeloid leukocyte activation
Phagocytic cup
Tumor necrosis factor production
Myeloid cell differentiation
E)
Microglia markers
p−value = 0
R = 0.95
Microglia marker metagene (log2)
RANK hypothalamus metagene (log2)
F)
Microglia signature
RANK metagene
p = 1,14 * 10 -10
91
9
64
ITGAX
BIN2
TMEM173
PTAFR
TGFBR1
PTGS1
FAM105A
CD53
CYFIP1

Fig. S5. Hypothalamic microglia are the main Rank-positive population in human and mice (A) Left plot: *Rank* expression in mouse hypothalamus from an integrated analysis of 17 scRNAseq datasets, including embryonic and adult mice (PMID: 36266547); Right plot: *RANK* expression in human hypothalamus scRNAseq dataset from human embryos (GSE169109). (B-C) RANK and RANK metagene expression in the mouse hypothalamus from panel A, separated by sex, and in the human hypothalamus (PMID: 40000749) separated by sex. (D) Gene Ontology pathway analysis of RANK metagene. (E) Correlation between RANK metagene and microglia gene signature generated by panglaodb in human hypothalamus tissue (GTEx). (F) Venn diagram of RANK hypothalamic metagene and microglia gene signature by panglaodb indicating the common genes between both signatures.

Figure S6

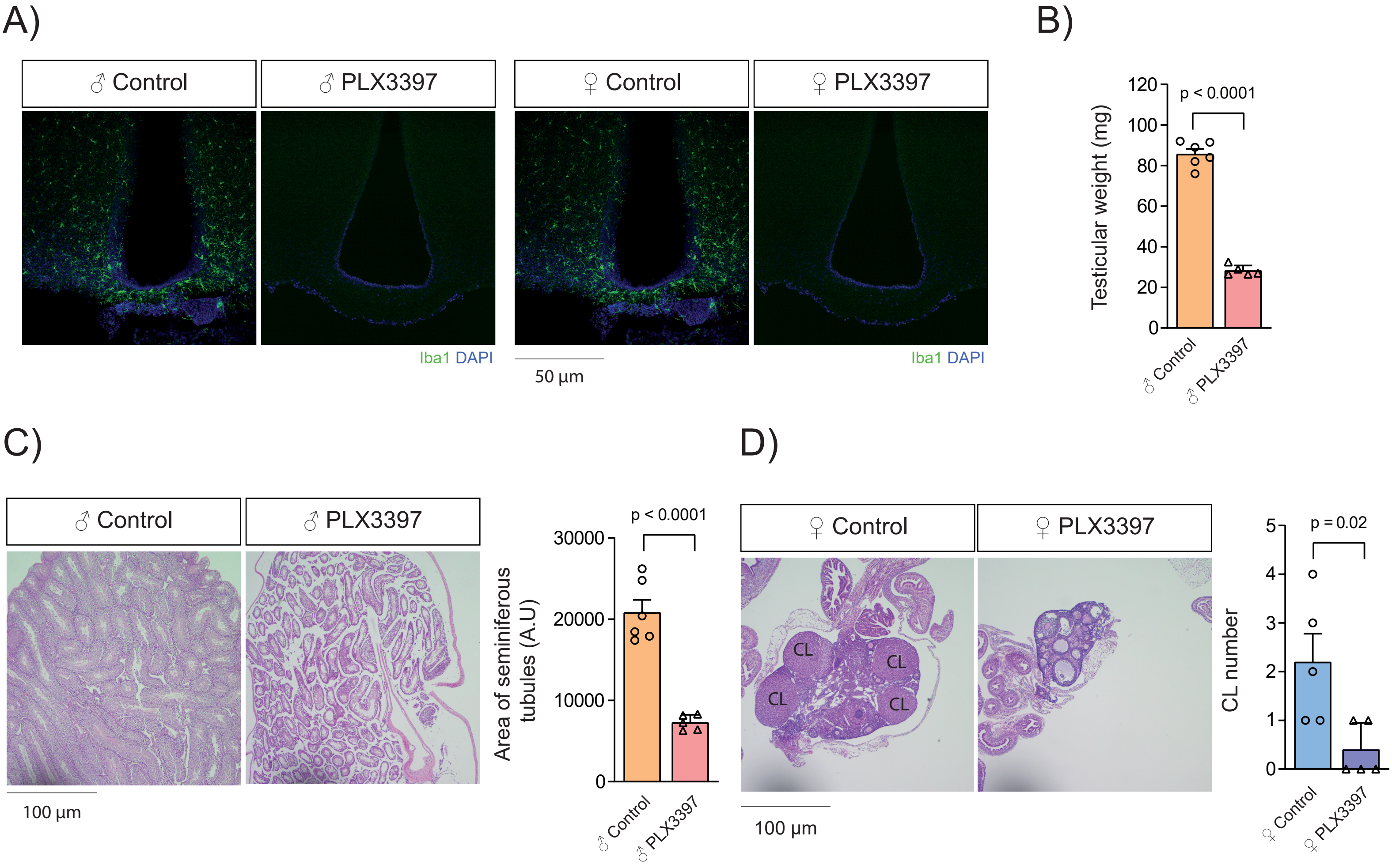

A)
♂ Control
♂ PLX3397
♀ Control
♀ PLX3397
Iba1 DAPI
Iba1 DAPI
50 µm
B)
p < 0.0001
Testicular weight (mg)
120
100
80
60
40
20
0
♂ Control
♂ PLX3397
C)
♂ Control
♂ PLX3397
100 µm
p < 0.0001
Area of seminiferous tubules (A.U)
30000
20000
10000
0
♂ Control
♂ PLX3397
D)
♀ Control
♀ PLX3397
CL
CL
CL
CL
100 µm
p = 0.02
CL number
5
4
3
2
1
0
♀ Control
♀ PLX3397

Fig. S6. Microglia depletion in mice disrupts gonadal development (A) Representative immunofluorescence (IF) images of microglia cells (Iba1+ in green) in the mediobasal hypothalamus from control and PLX3397-treated mice from both sexes. Nuclei are labelled with DAPI (blue). (B) Testicular weight of control and PLX3397-treated male mice. (C) Representative images of H&E staining of testes of control and PLX3397-treated male mice and quantification of the area of their seminiferous tubules. (D) Representative images of H&E staining of ovaries of control and PLX3397-treated female mice and quantification of corpora lutea (CL). Analyses were performed in 8-week-old mice (A-D). Data are represented as mean +/-SEM, with each dot representing a mouse; P values were calculated using two-tailed unpaired t-test and exact P values are indicated when statistically significant (B-D).

Figure S7

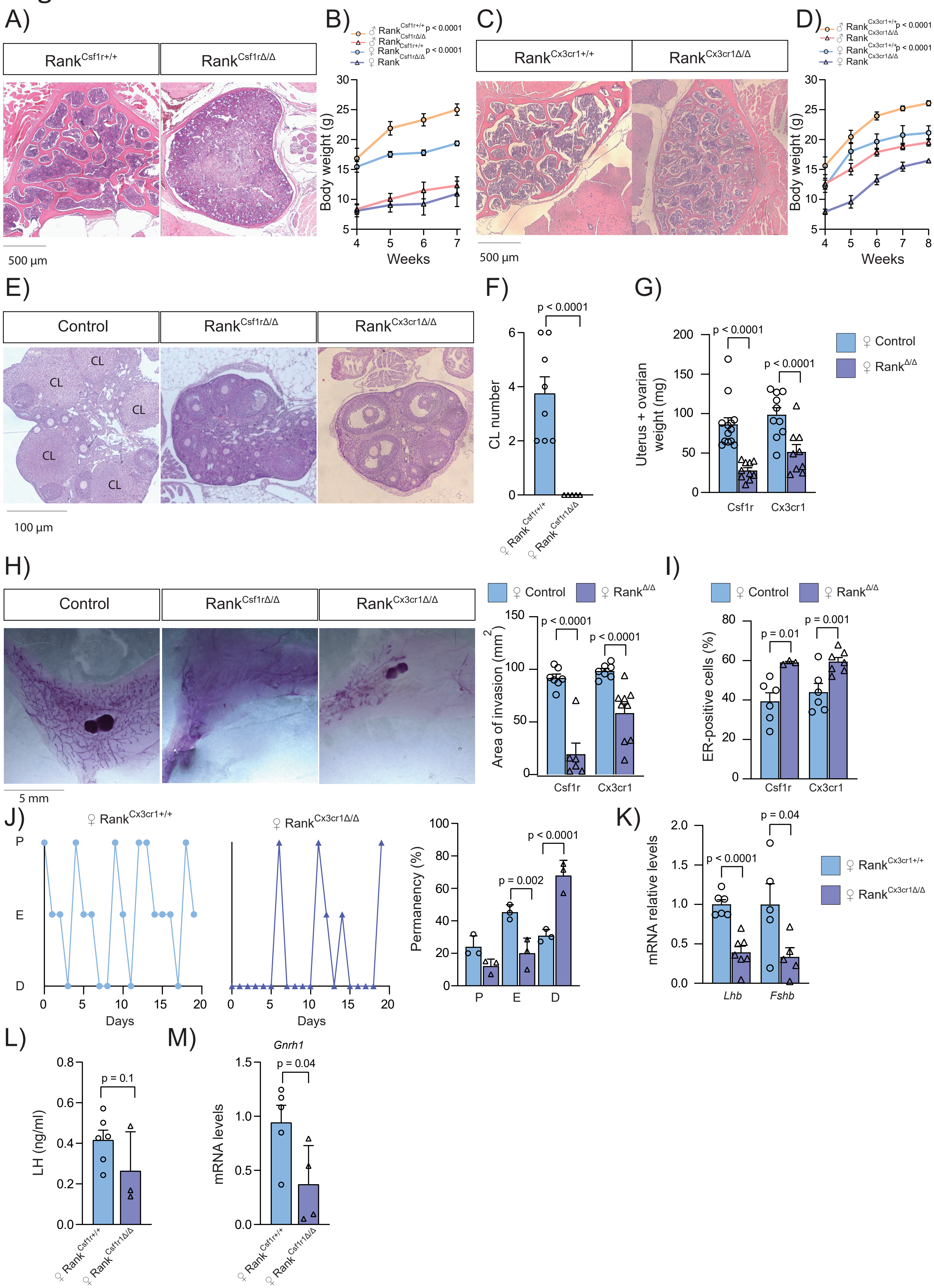

A)
B)
C)
D)
E)
F)
G)
H)
I)
J)
K)
L)
M)
Control
CL
500 µm
100 µm
5 mm
Body weight (g)
Weeks
CL number
Uterus + ovarian weight (mg)
Area of invasion (mm2)
ER-positive cells (%)
Permanency (%)
mRNA relative levels
LH (ng/ml)
mRNA levels
Days
Csf1r
Cx3cr1
Lhb
Fshb
Gnrh1
p < 0.0001
p = 0.01
p = 0.001
p = 0.002
p = 0.04
p = 0.1

Fig. S7. Rank deletion in Csf1r and Cx3cr1 cells impairs the HPG axis in females (A) H&E staining of vertebral bones from $Rank^{Csf1r\Delta/\Delta}$ and control females. (B) Body weight of $Rank^{Csf1r+/+}$ and $Rank^{Csf1r\Delta/\Delta}$ mice by age and sex. (C) Representative images of H&E staining of vertebral bones from $Rank^{Cx3cr1+/+}$ and $Rank^{Cx3cr1\Delta/\Delta}$ mice. (D) Body weight of $Rank^{Cx3cr1+/+}$ and $Rank^{Cx3cr1\Delta/\Delta}$ mice by age and sex. (E) Representative images of H&E staining of control, $Rank^{Csf1r\Delta/\Delta}$ and $Rank^{Cx3cr1\Delta/\Delta}$ ovaries. (D) Quantification of corpora lutea (CL) in ovaries from control and $Rank^{Csf1r\Delta/\Delta}$ mice. (G) Uterus plus ovarian weight from $Rank^{Csf1r\Delta/\Delta}$ and $Rank^{Cx3cr1\Delta/\Delta}$ mice and control mice. (H) Representative images of whole mounts and quantification of epithelial invasion in mammary glands from $Rank^{Csf1r\Delta/\Delta}$, $Rank^{Cx3cr1\Delta/\Delta}$ and corresponding control mice. (I) ER+ quantification in mammary glands from $Rank^{Csf1r\Delta/\Delta}$, $Rank^{Cx3cr1\Delta/\Delta}$ and corresponding control mice. (J) Estrous cyclicity in adult $Rank^{Cx3cr1\Delta/\Delta}$ females and controls. Percentage of permanency in proestrus (P), estrus (E), or diestrus (D) is provided. (K) *Lhb* and *Fshb* mRNA expression amounts in the pituitary gland from $Rank^{Cx3cr1\Delta/\Delta}$ females relative to control littermates. (L) LH concentration in blood from $Rank^{Csf1r+/+}$ and $Rank^{Cs1fr\Delta/\Delta}$ female mice (M) *Gnrh1* mRNA expression amounts in the hypothalamus from $Rank^{Csf1r\Delta/\Delta}$ females and control littermates. Analyses were performed in 8-week-old mice (E-I; K-M); data are represented as mean +/-SEM with each dot representing a mouse; P values were calculated using two-way ANOVA followed by Tukey's multiple comparisons (B-D) or two-tailed unpaired t-test (F-G, H-M) and exact P values are indicated when statistically significant.

Figure S8

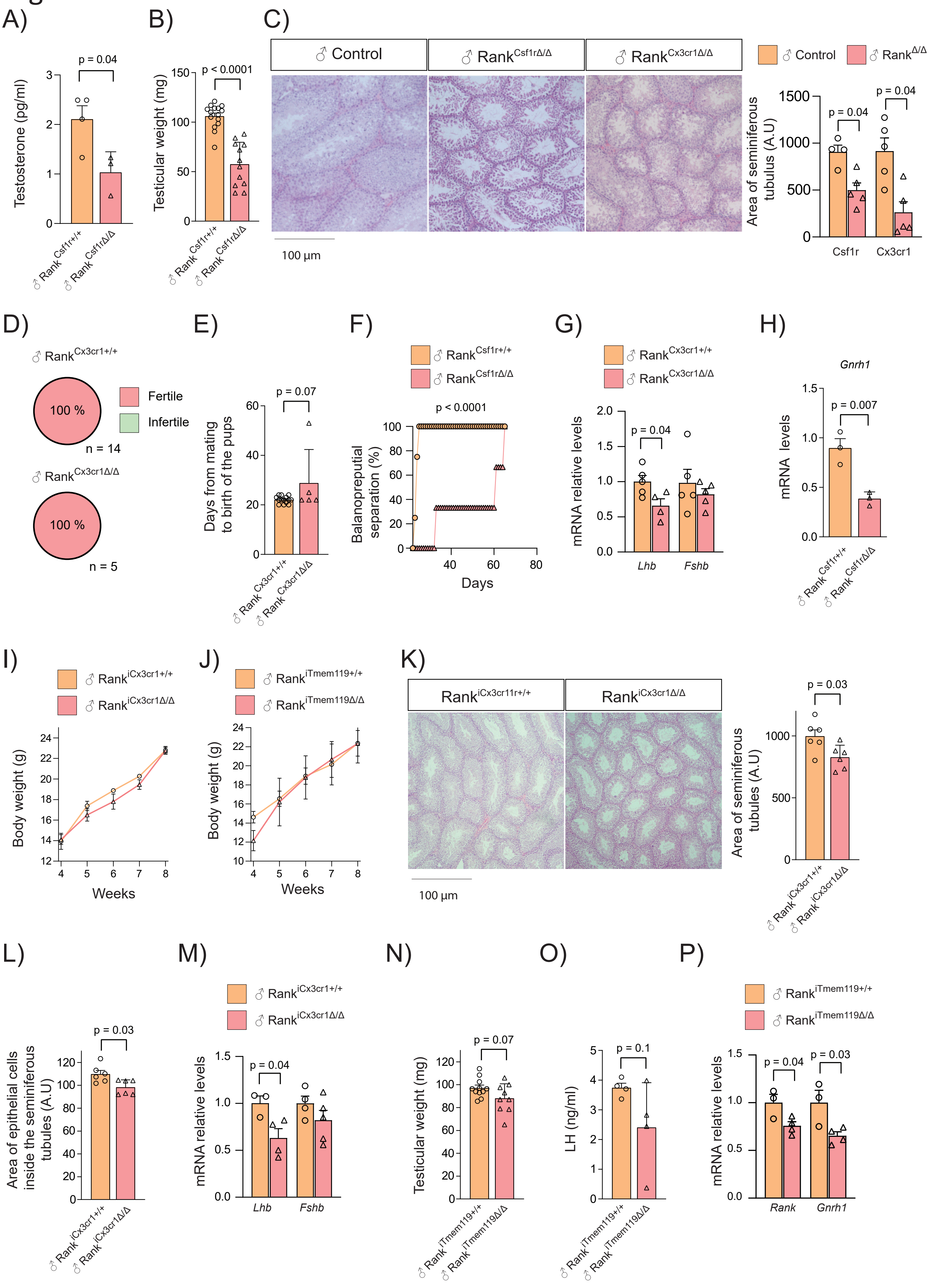
A)
p = 0.04
Testosterone (pg/ml)
♂ Rank Csf1r+/+
♂ Rank Csf1rΔ/Δ
B)
p < 0.0001
Testicular weight (mg)
C)
♂ Control
♂ RankCsf1rΔ/Δ
♂ RankCx3cr1Δ/Δ
100 µm
♂ Control
♂ RankΔ/Δ
Area of seminiferous tubulus (A.U)
Csf1r
Cx3cr1
D)
♂ RankCx3cr1+/+
100 %
n = 14
Fertile
Infertile
♂ RankCx3cr1Δ/Δ
n = 5
E)
p = 0.07
Days from mating to birth of the pups
F)
♂ RankCsf1r+/+
♂ RankCsf1rΔ/Δ
p < 0.0001
Balanopreputial separation (%)
Days
G)
♂ RankCx3cr1+/+
♂ RankCx3cr1Δ/Δ
p = 0.04
mRNA relative levels
Lhb
Fshb
H)
Gnrh1
p = 0.007
mRNA levels
I)
♂ RankiCx3cr1+/+
♂ RankiCx3cr1Δ/Δ
Body weight (g)
Weeks
J)
♂ RankiTmem119+/+
♂ RankiTmem119Δ/Δ
K)
RankiCx3cr11r+/+
RankiCx3cr1Δ/Δ
p = 0.03
Area of seminiferous tubules (A.U)
L)
Area of epithelial cells inside the seminiferous tubules (A.U)
M)
N)
p = 0.07
O)
p = 0.1
LH (ng/ml)
P)
p = 0.04
p = 0.03
Rank
Gnrh1

Fig. S8. Inducible and constitutive Rank deletion in microglia impairs the HPG axis in males (A) Testosterone concentration in serum from $Rank^{Csf1r\Delta/\Delta}$ male mice and control littermates**.** (B) Testicular weight of $Rank^{Csf1r\Delta/\Delta}$ male mice and control littermates. (C) Representative images of H&E staining of testes and quantification of the area of their seminiferous tubules in $Rank^{Csf1r\Delta/\Delta}$, $Rank^{Csf1r\Delta/\Delta}$ and corresponding control littermates. (D) Percentage of fertile $Rank^{Cx3cr1+/+}$ (n = 14) and $Rank^{Cx3cr1\Delta/\Delta}$ male mice (n=5). (E) Days from mating to the birth of the pups of animals from panel (D). (F) Graphs showing the cumulative percentage of males that achieved balanopreputial separation in $Rank^{Cx3cr1\Delta/\Delta}$ and control male mice. Statistical differences were tested using the Gehan-Breslow-Wilcoxon matched-pairs test: $\chi^2 = 4.898$, P = 0.03, n = 4 and n = 3. (G) *Lhb* and *Fshb* mRNA amounts in the pituitary gland from $Rank^{Cx3cr1\Delta/\Delta}$ males relative to control littermates. (H) *Gnrh1* mRNA amounts in the hypothalamus from $Rank^{Csf1r\Delta/\Delta}$ male and control littermates. (I) Body weight of $Rank^{iCx3cr1+/+}$ and $Rank^{iCx3cr1\Delta/\Delta}$ males at indicated age (in weeks) upon Rank deletion at puberty. (J) Body weight of $Rank^{iTmem119+/+}$ and $Rank^{iTmem119\Delta/\Delta}$ males at indicated age (in weeks) upon Rank deletion at puberty. (K) Representative images of H&E staining of control and $Rank^{iCx3cr1\Delta/\Delta}$ testes and quantification of the area of their seminiferous tubules. (L) Area of the epithelial cells inside each seminiferous tubule from mice in panel K. (M) *Lhb* and *Fshb* mRNA amounts in the pituitary gland from $Rank^{iCx3cr1\Delta/\Delta}$ male mice and control littermates. (N) Testicular weight of $Rank^{iTmem119\Delta/\Delta}$ male mice and control littermates (O) LH concentration in blood from $Rank^{iTmem119\Delta/\Delta}$ and control male mice. (P) *Rank* and *Gnrh1* mRNA amounts in the hypothalamus from $Rank^{iTmem119\Delta/\Delta}$ and control male mice. Analyses were performed in 8-week-old mice (A-E; G-H;K-P); data are represented as mean +/-SEM with each dot representing a mouse; P values were calculated using two-way ANOVA followed by Tukey's multiple comparisons (I-J) or two-tailed unpaired t-test (A-C; E; G-H, L-P), and exact P values are indicated when statistically significant. $Rank^{iCx3cr1\Delta/\Delta}$, $Rank^{iTmem119\Delta/\Delta}$ male mice and corresponding controls were TAM treated as shown in Fig. 1J.

Figure S9

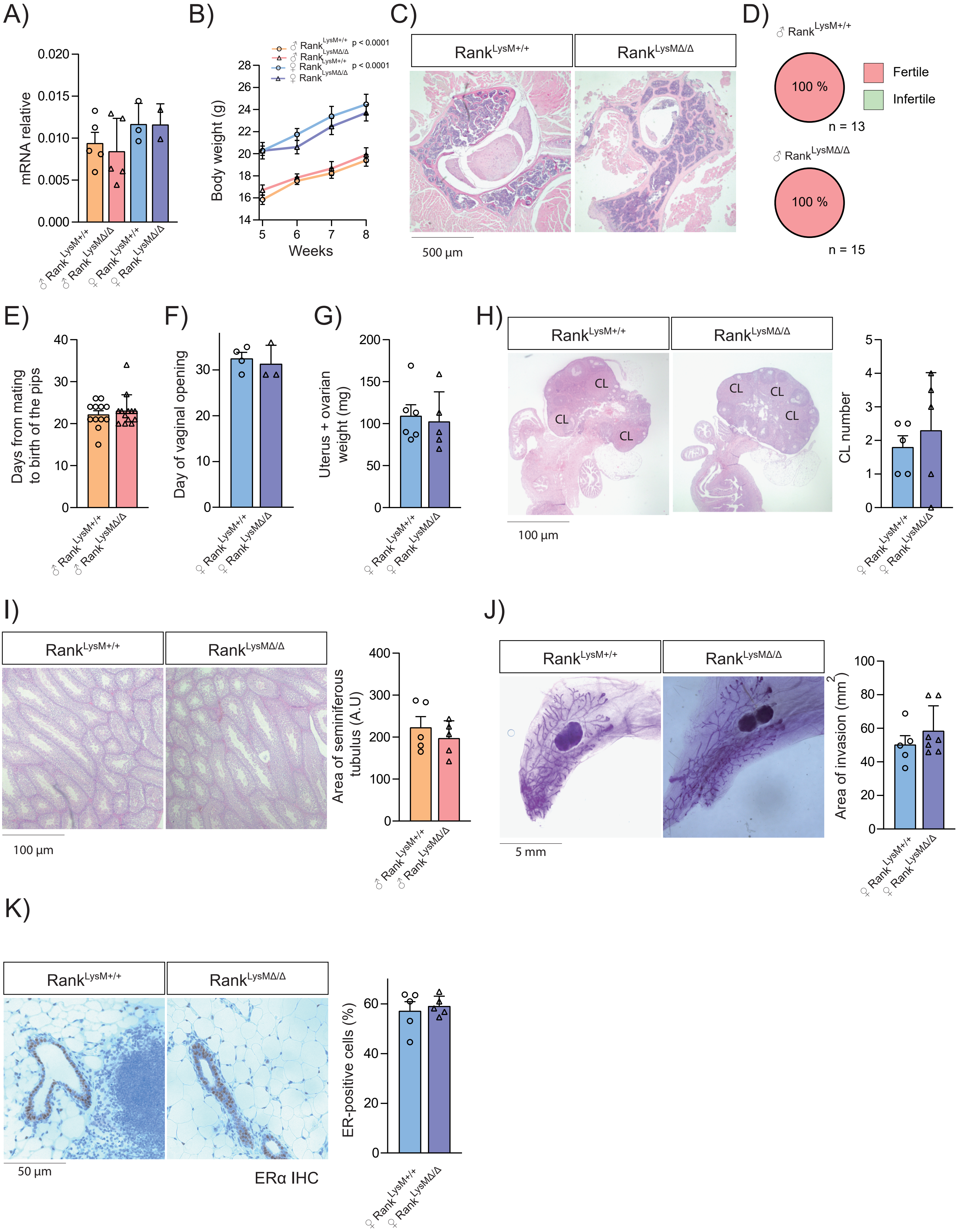

A)
mRNA relative
0.020
0.015
0.010
0.005
0.000
♂ Rank LysM+/+
♂ Rank LysMΔ/Δ
♀ Rank LysM+/+
♀ Rank LysMΔ/Δ
B)
Body weight (g)
Weeks
p < 0.0001
p < 0.0001
C)
Rank LysM+/+
Rank LysMΔ/Δ
500 µm
D)
♂ Rank LysM+/+
♂ Rank LysMΔ/Δ
100 %
n = 13
n = 15
Fertile
Infertile
E)
Days from mating to birth of the pips
F)
Day of vaginal opening
G)
Uterus + ovarian weight (mg)
H)
CL
CL number
100 µm
I)
Area of seminiferous tubulus (A.U)
100 µm
J)
Area of invasion (mm2)
5 mm
K)
ER-positive cells (%)
50 µm
ERα IHC

Fig. S9. Rank loss in peripheral macrophages does not impair the HPG axis. (A) mRNA of *Rank* amounts in the hypothalamus of $\text{Rank}^{\text{LysM}\Delta/\Delta}$ male and female mice and control littermates. (B) Body weight of $\text{Rank}^{\text{LysM}+/+}$ and $\text{Rank}^{\text{LysM}\Delta/\Delta}$ mice at the indicated age and sex. (C) Representative images of H&E staining of vertebral bones from $\text{Rank}^{\text{LysM}/+}$ and $\text{Rank}^{\text{LysM}\Delta/\Delta}$ mice. (D) Percentage of fertile $\text{Rank}^{\text{LysM}+/+}$ (n=13) and $\text{Rank}^{\text{LysM}\Delta/\Delta}$ (n=16) male mice. (E) Days from mating to the birth of the pups of animals from panel d. (F) Day of vaginal opening in $\text{Rank}^{\text{LysM}+/+}$ and $\text{Rank}^{\text{LysM}\Delta/\Delta}$ female mice. (G) Uterus plus ovarian weight in $\text{Rank}^{\text{LysM}\Delta/\Delta}$ and control littermates. (H) Representative images of H&E staining of $\text{Rank}^{\text{LysM}+/+}$ and $\text{Rank}^{\text{LysM}\Delta/\Delta}$ ovaries and quantification of corpora lutea (CL). (I) Representative images of H&E staining of $\text{Rank}^{\text{LysM}+/+}$ and $\text{Rank}^{\text{LysM}\Delta/\Delta}$ testes and quantification of the area of seminiferous tubules. (J) Representative images of whole mounts and quantification of epithelial invasion in mammary glands from $\text{Rank}^{\text{LysM}+/+}$ and $\text{Rank}^{\text{LysM}\Delta/\Delta}$ females. (K) Representative images of ERα expression and quantifications of ER-positive cells relative to epithelial cells in $\text{Rank}^{\text{LysM}+/+}$ and $\text{Rank}^{\text{LysM}\Delta/\Delta}$ mammary glands. Analyses were performed in 8-week-old mice (A, C-K); data are represented as mean +/-SEM with each dot representing a mouse; P values were calculated using two-way ANOVA followed by Tukey's multiple comparisons (A,B) or two-tailed unpaired t-test (E-K), and exact P values are indicated when statistically significant.

Figure S10

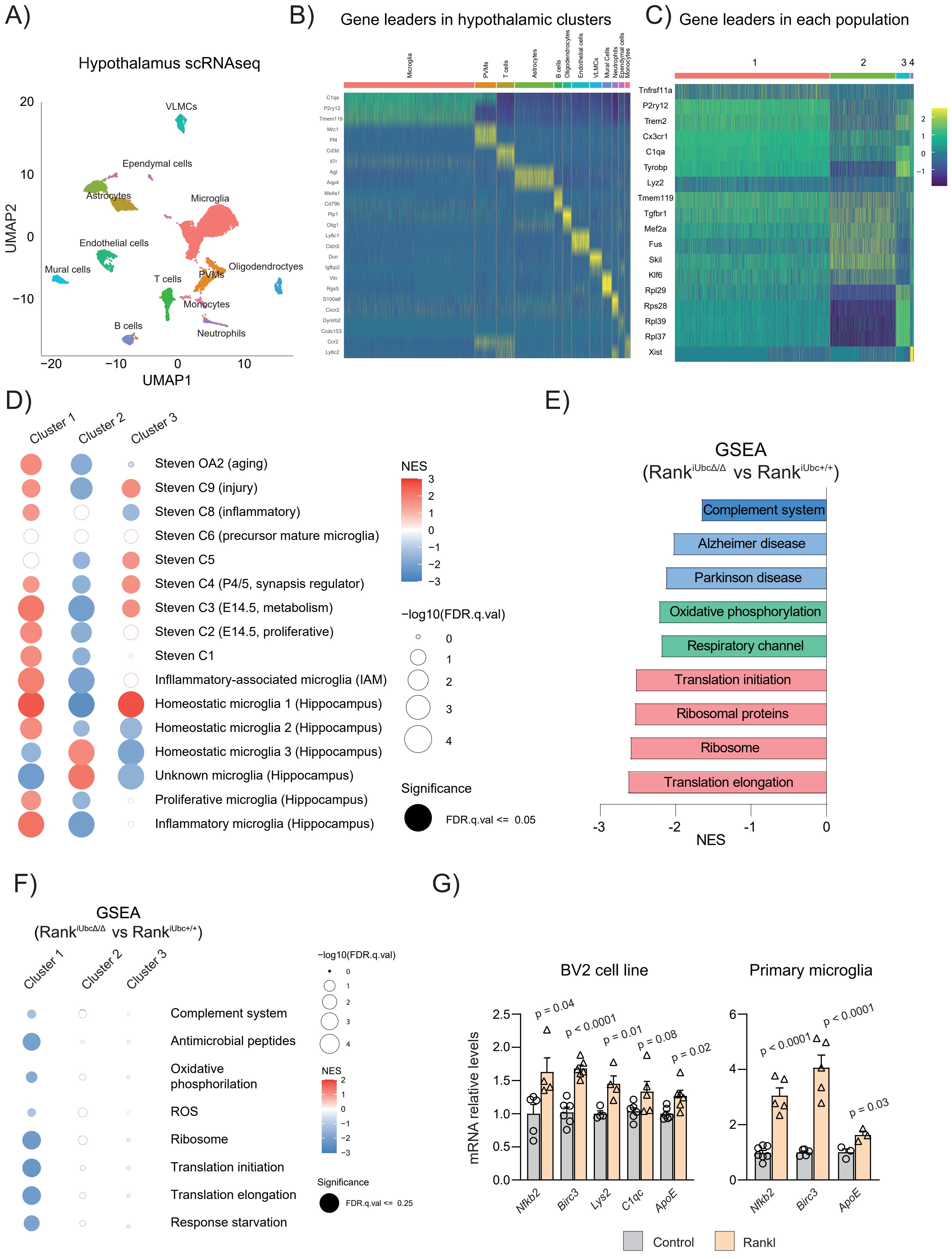

A)
Hypothalamus scRNAseq
VLMCs
Ependymal cells
Astrocytes
Microglia
Endothelial cells
Mural cells
Oligodendroctyes
PVMs
T cells
Monocytes
B cells
Neutrophils
UMAP2
UMAP1
B)
Gene leaders in hypothalamic clusters
Microglia
PVMs
T cells
Astrocytes
B cells
Oligodendrocytes
Endothelial cells
VLMCs
Mural Cells
Neutrophils
Ependymal cells
Monocytes
C1qa
P2ry12
Tmem119
Mrc1
Pf4
Cd3d
Il7r
Agt
Aqp4
Ms4a1
Cd79b
Plp1
Olig1
Ly6c1
Cldn5
Dcn
Igfbp2
Vtn
Rgs5
S100a8
Cxcr2
Dynlrb2
Ccdc153
Ccr2
Ly6c2
C)
Gene leaders in each population
1
2
3 4
Tnfrsf11a
P2ry12
Trem2
Cx3cr1
C1qa
Tyrobp
Lyz2
Tmem119
Tgfbr1
Mef2a
Fus
Skil
Klf6
Rpl29
Rps28
Rpl39
Rpl37
Xist
D)
Cluster 1
Cluster 2
Cluster 3
Steven OA2 (aging)
Steven C9 (injury)
Steven C8 (inflammatory)
Steven C6 (precursor mature microglia)
Steven C5
Steven C4 (P4/5, synapsis regulator)
Steven C3 (E14.5, metabolism)
Steven C2 (E14.5, proliferative)
Steven C1
Infllammatory-associated microglia (IAM)
Homeostatic microglia 1 (Hippocampus)
Homeostatic microglia 2 (Hippocampus)
Homeostatic microglia 3 (Hippocampus)
Unknown microglia (Hippocampus)
Proliferative microglia (Hippocampus)
Inflammatory microglia (Hippocampus)
NES
−log10(FDR.q.val)
Significance
FDR.q.val <= 0.05
E)
GSEA
(Rank iUbcΔ/Δ vs Rank iUbc+/+)
Complement system
Alzheimer disease
Parkinson disease
Oxidative phosphorylation
Respiratory channel
Translation initiation
Ribosomal proteins
Ribosome
Translation elongation
NES
F)
GSEA
(Rank iUbcΔ/Δ vs Rank iUbc+/+)
Cluster 1
Cluster 2
Cluster 3
Complement system
Antimicrobial peptides
Oxidative phosphorylation
ROS
Ribosome
Translation initiation
Translation elongation
Response starvation
−log10(FDR.q.val)
NES
Significance
FDR.q.val <= 0.25
G)
BV2 cell line
Primary microglia
mRNA relative levels
p = 0.04
p < 0.0001
p = 0.01
p = 0.08
p = 0.02
p < 0.0001
p < 0.0001
p = 0.03
Nfkb2
Birc3
Lys2
C1qc
ApoE
Control
Rankl

Fig. S10. scRNA-seq analysis of hypothalamic microglia reveals a predominant active microglial population that is severely affected by Rank loss. (A) Uniform Manifold Approximation and Projection (UMAP) technique identified 12 cell clusters, represented in the graph as different cell populations. UMAP plot shows 5.807 $Rank^{iUbc+/+}$ cells and 3.990 $Rank^{iUbc\Delta/\Delta}$ cells. PVMs = Perivascular macrophages. VLMCs = Vascular and leptomeningeal cells. (B) Gene-expression heatmap of the selected marker genes of each cluster compared to all other clusters. Genes are represented in rows and cell clusters in columns. (C) Gene-expression heatmap of selected markers of each microglia cluster compared to all other clusters in scRNAseq of hypothalamus. Genes are represented in rows and cell clusters in columns. The fourth microglia cluster did not pass the quality control due to its limited abundance (57 out of 2,100 microglia cells) and the expression of the female-specific gene *Xist* in males. (D) Bubble plots depicting the variation of selected gene set signatures obtained from published scRNAseq data sets in cluster 1, 2 and 3 compared to other clusters. Filled bubbles indicate enriched significant gene signatures (FDR < 0.05). Red and blue colors denote upregulated and downregulated, respectively. (E) Bar plot depicting the variation of selected gene set signatures from $Rank^{iUbc\Delta/\Delta}$ versus control $Rank^{iUbc+/+}$ in microglia cells (FDR > 0.05; NES < -1.5). (F) Bubble plots depicting the variation of selected gene set signatures between $Rank^{iUbc\Delta/\Delta}$ and control microglia within cluster 1, 2 and 3. Filled bubbles indicate enriched significant gene signatures (FDR < 0.25). Red color denotes upregulated, and blue downregulated gene sets. $Rank^{iUbc\Delta/\Delta}$ and $Rank^{iUbc+/+}$ male mice were TAM treated as shown in Fig. 1J and analyses were performed on 8-week-old mice (A-G). Data (A-D) derive from the combination of the two genotypes (H) mRNA analysis of the indicated genes in control and Rankl-treated BV2 cell line and primary microglial cells 6 hours after Rankl treatment (100ng/µL). Data are represented as mean +/-SEM. P values were calculated using two-tailed unpaired t-test and exact P values are indicated.

Figure S11

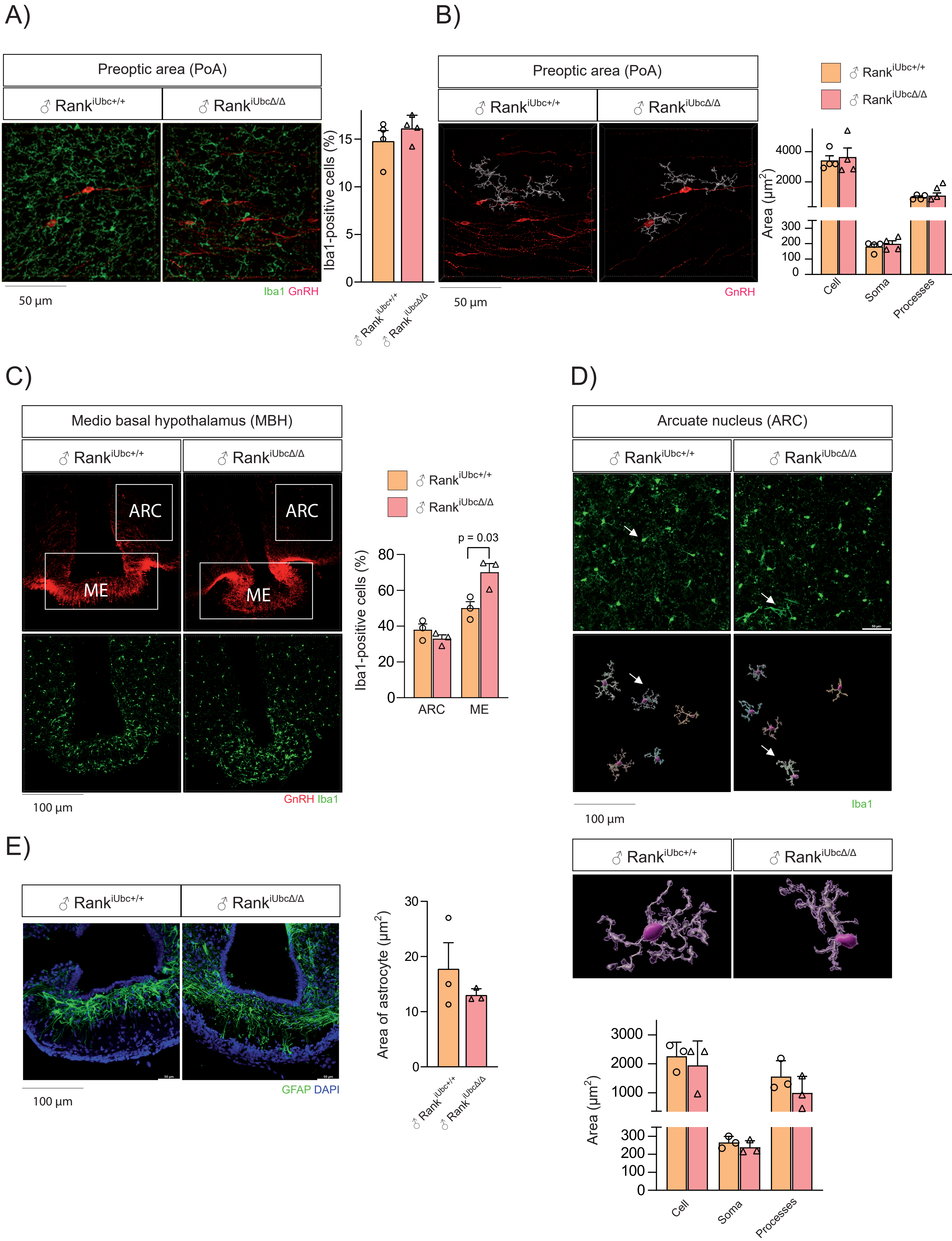
A)
Preoptic area (PoA)
♂ RankiUbc+/+
♂ RankiUbcΔ/Δ
50 µm
Iba1 GnRH
Iba1-positive cells (%)
B)
Preoptic area (PoA)
♂ RankiUbc+/+
♂ RankiUbcΔ/Δ
50 µm
GnRH
Area (µm2)
Cell
Soma
Processes
C)
Medio basal hypothalamus (MBH)
♂ RankiUbc+/+
♂ RankiUbcΔ/Δ
ARC
ME
100 µm
GnRH Iba1
p = 0.03
Iba1-positive cells (%)
D)
Arcuate nucleus (ARC)
♂ RankiUbc+/+
♂ RankiUbcΔ/Δ
100 µm
Iba1
Area (µm2)
Cell
Soma
Processes
E)
♂ RankiUbc+/+
♂ RankiUbcΔ/Δ
100 µm
GFAP DAPI
Area of astrocyte (µm2)

Fig. S11. Rank loss does not alter microglia morphology or number in the preoptic area (PoA) or arcuate nucleus (ARC). (A) Representative IF images of the hypothalamic microglia cells (Iba1+ in green) and GnRH neurons (red) in the Organum Vasculosum of the Lamina Terminalis (OVLT) of the PoA of $Rank^{iUbc\Delta/\Delta}$ and control $Rank^{iUbc+/+}$ upon pubertal Rank loss. Nuclei are labelled with DAPI (blue). Corresponding quantification of number microglia cells around GnRH cells bodies was provided. (B) Morphological analysis of control and $Rank^{iUbc\Delta/\Delta}$ hypothalamic microglia in the PoA, showing reconstructed microglia using IMARIS surrounding GnRH neurons (red). Quantification includes the total area, area of the microglia soma, and processes area. (C) Representative IF images of microglia cells (Iba1+ in green) and GnRH particles (GnRH in red) in the medio basal hypothalamus from $Rank^{iUbc\Delta/\Delta}$ and controls in the arcuate nucleus (ARC) and median eminence (ME). Nuclei are labelled with DAPI (blue). Corresponding quantification of microglia cells in the ARC and ME are provided. (D) Representative IF images of hypothalamic microglia cells (Iba1+ in green) in the ARC of $Rank^{iUbc\Delta/\Delta}$ and controls. Reconstructed microglia images (white arrows) generated using IMARIS software are shown. Quantification of microglia morphological parameters, including total microglial area, cell body area and microglia processes area are shown. (E) Representative IF images of astrocytes (GFAP+ in green) in the ME of $Rank^{iUbc\Delta/\Delta}$ and controls. Nuclei are labelled with DAPI (blue). Corresponding quantification of astrocyte area is provided. Data are represented as mean +/-SEM with each dot representing a mouse; P values were calculated using two-tailed unpaired t-test (A-C, E-F), and exact P values are indicated when statistically significant. $Rank^{iUbc\Delta/\Delta}$ and $Rank^{iUbc+/+}$ male mice were TAM treated as shown in Fig. 1J.

Figure S12

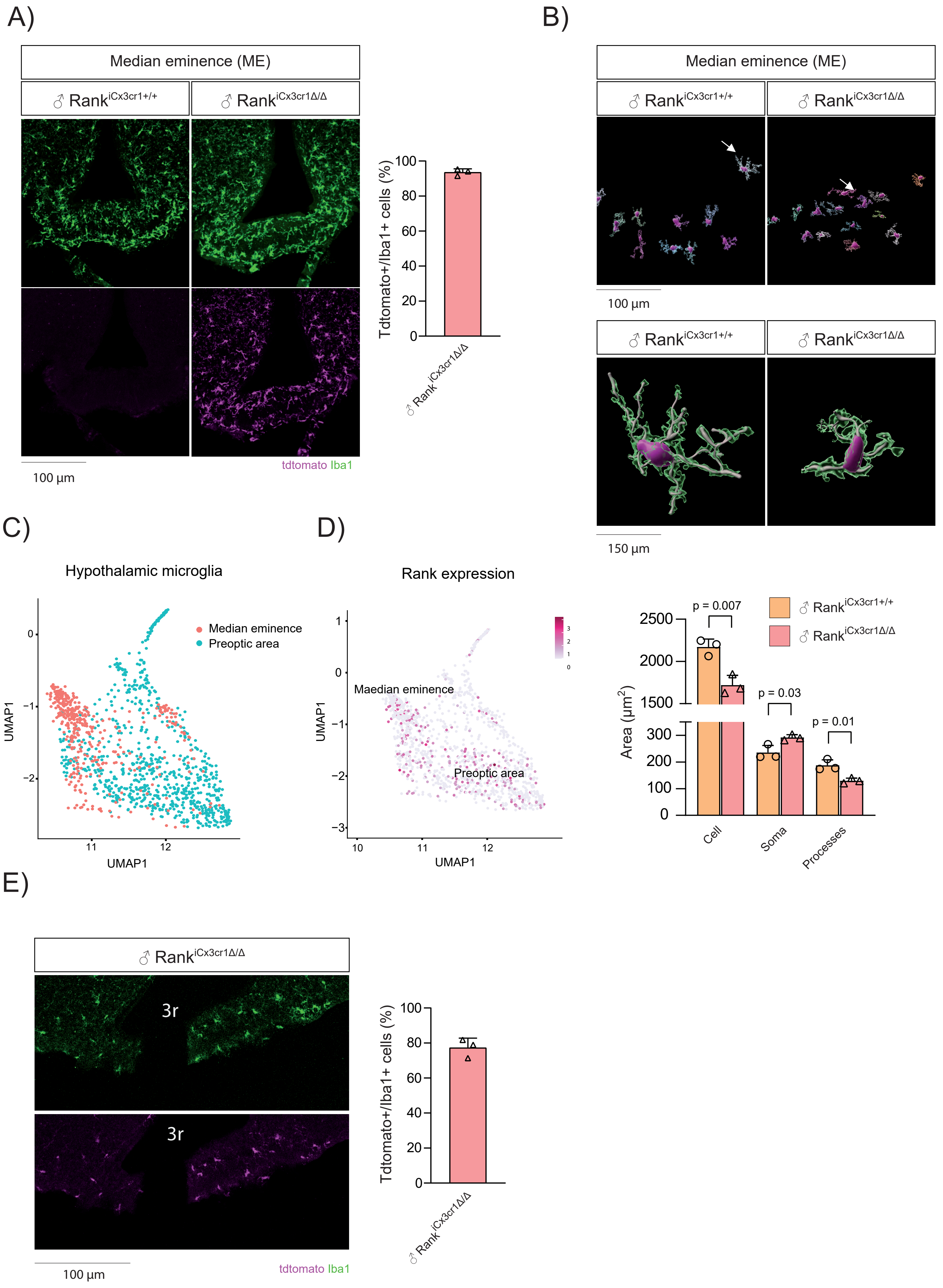

A)
Median eminence (ME)
♂ RankiCx3cr1+/+
♂ RankiCx3cr1Δ/Δ
100 µm
tdtomato Iba1
Tdtomato+/Iba1+ cells (%)
0
20
40
60
80
100
♂ RankiCx3cr1Δ/Δ
B)
Median eminence (ME)
♂ RankiCx3cr1+/+
♂ RankiCx3cr1Δ/Δ
100 µm
♂ RankiCx3cr1+/+
♂ RankiCx3cr1Δ/Δ
150 µm
C)
Hypothalamic microglia
Median eminence
Preoptic area
UMAP1
UMAP1
0
−1
−2
11
12
D)
Rank expression
Maedian eminence
Preoptic area
UMAP1
UMAP1
1
0
−1
−2
−3
10
11
12
3
2
1
0
p = 0.007
p = 0.03
p = 0.01
♂ RankiCx3cr1+/+
♂ RankiCx3cr1Δ/Δ
Area (µm2)
0
100
200
300
1500
2000
2500
Cell
Soma
Processes
E)
♂ RankiCx3cr1Δ/Δ
3r
3r
100 µm
tdtomato Iba1
Tdtomato+/Iba1+ cells (%)
0
20
40
60
80
100
♂ RankiCx3cr1Δ/Δ

Fig. S12. Microglia Rank loss induces morphological changes in microglia from the median eminence (ME). (A) Representative IF images of the microglia cells (Iba1+ in green) and tdtomato staining (magenta) in the ME of control and Rank$^{iCx3cr1\Delta/\Delta}$ mice bred with tdtomato reporter line. Quantification of recombination efficiency, showing the percentage of double-positive iba1/tdtomato-positive cells, is shown. (B) Morphological analysis of control and Rank$^{iCx3cr1\Delta/\Delta}$ microglia in the ME, showing reconstructed microglia using IMARIS. Corresponding quantification of total area, area of the soma, and processes area is provided. P values were calculated using two-tailed unpaired t-test and exact P values are indicated. Rank$^{iCx3cr1\Delta/\Delta}$ male mice and corresponding controls were TAM treated as shown in Fig. 1J (A-B). (C) UMAP of hypothalamic microglia with the combination of two public scRNAseq from the PoA (PMID: 30385464) and ME (PMID: 34260928). (D) Rank expression in microglia cells from the ME and PoA from panel C. (E) Representative IF images of the microglia cells (Iba1+ in green) and tdtomato staining (magenta) in the ME from control and Rank$^{iCx3cr1\Delta/\Delta}$ mice bred with tdtomato reporter line upon intracraneal tamoxifen treatment in the ME (as shown in Fig 3I) and analyses was performed in 18-week old mice. Quantification of recombination efficiency, showing the percentage of double-positive iba1/tdtomato-positive cells, is shown. Data are represented as mean +/-SEM with each dot representing a mouse (A-B, E).

Figure S13

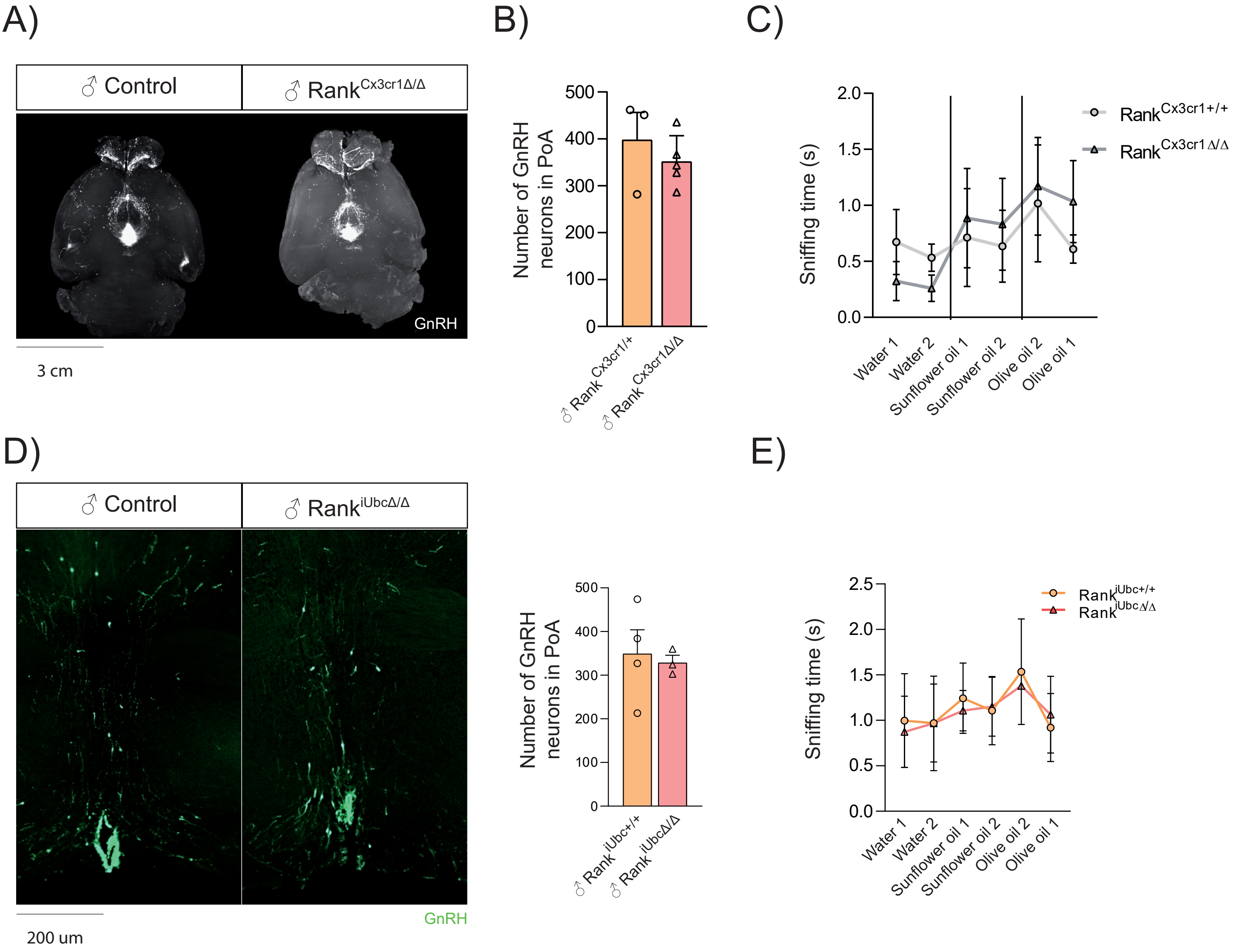

A)
♂ Control
♂ RankCx3cr1Δ/Δ
GnRH
3 cm
B)
Number of GnRH neurons in PoA
500
400
300
200
100
0
♂ RankCx3cr1/+
♂ RankCx3cr1Δ/Δ
C)
Sniffing time (s)
2.0
1.5
1.0
0.5
0.0
Water 1
Water 2
Sunflower oil 1
Sunflower oil 2
Olive oil 2
Olive oil 1
RankCx3cr1+/+
RankCx3cr1Δ/Δ
D)
♂ Control
♂ RankiUbcΔ/Δ
200 um
GnRH
Number of GnRH neurons in PoA
500
400
300
200
100
0
♂ RankiUbc+/+
♂ RankiUbcΔ/Δ
E)
Sniffing time (s)
2.5
2.0
1.5
1.0
0.5
0.0
Water 1
Water 2
Sunflower oil 1
Sunflower oil 2
Olive oil 2
Olive oil 1
RankiUbc+/+
RankiUbcΔ/Δ

Fig. S13. Embryonic or inducible Rank loss does not alter the number of GnRH neurons. (A) Representative view of whole-mount GnRH immunoreactivity in adult $Rank^{Cx3cr1\Delta/\Delta}$ males and controls by iDISCO. (B) Quantification of number of GnRH immunoreactive cell bodies in the preoptic area (PoA) of control and $Rank^{Cx3cr1\Delta/\Delta}$ mice. (C) Sniffing time of $Rank^{Cx3cr1\Delta/\Delta}$ (n = 4) and control (n = 5) male and female mice in habituation and dishabituation tests after exposure to water, sunflower oil, and olive oil. (D) Representative IF image of GnRH immunoreactive cell bodies in the Organum Vasculosum of the Lamina Terminalis (OVLT) of the preoptic area of control and $Rank^{iUbc\Delta/\Delta}$ mice after Rank depletion at puberty and quantification. (E) Sniffing time of $Rank^{iUbc\Delta/\Delta}$ (n = 4) and control (n = 5) males in habituation and dishabituation tests after exposure to water, sunflower oil, and olive oil. Data are represented as mean +/-SEM, with each dot representing a mouse; P values were calculated by unpaired two-tailed t-test (B, D) and two-way ANOVA followed by Tukey's multiple comparisons (C, E) and P values are indicated when statistically significant. $Rank^{iUbc\Delta/\Delta}$ and $Rank^{iUbc+/+}$ male mice were TAM treated as shown in Fig. 1J.

Figure S14

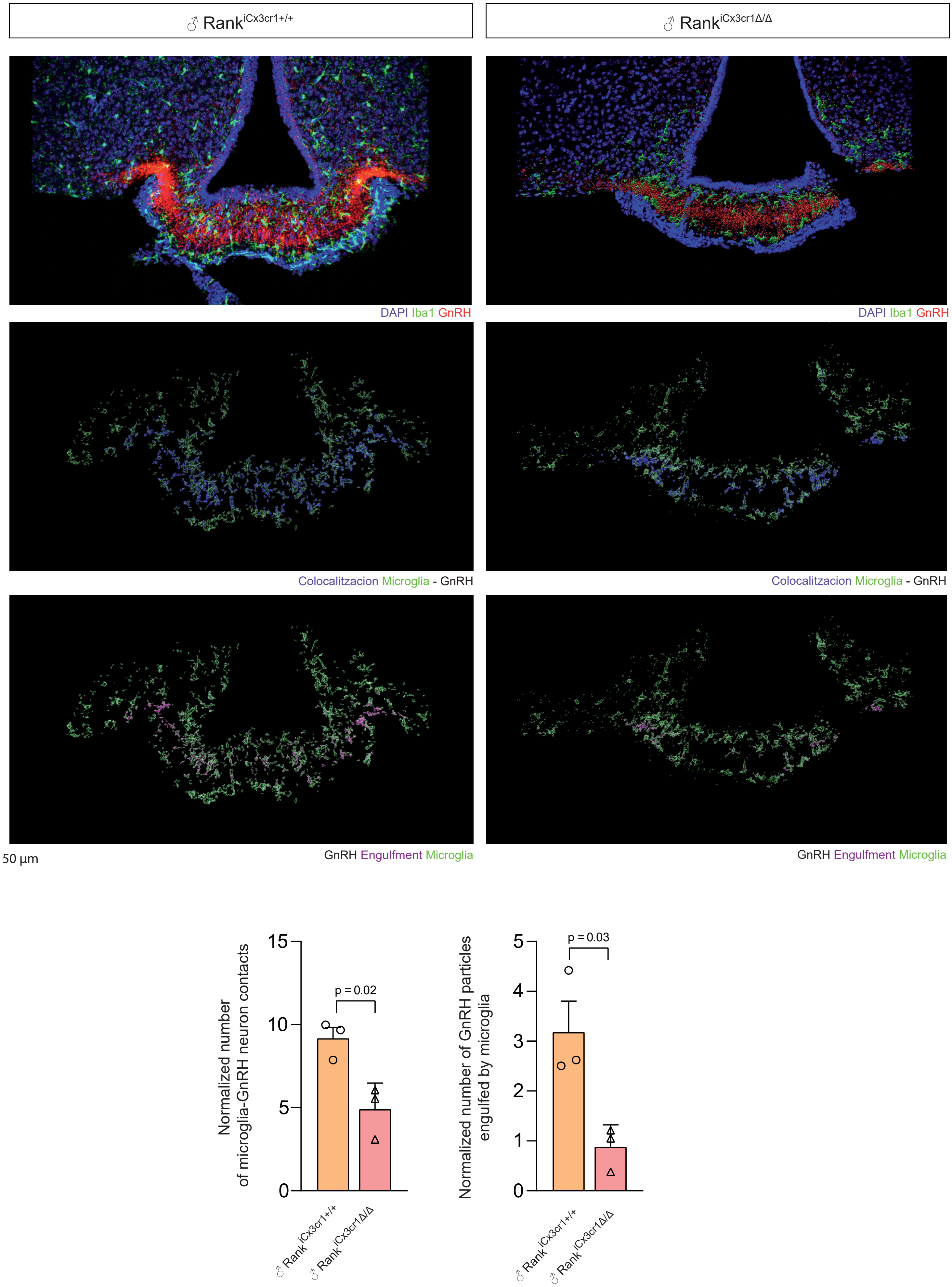

♂ Rank iCx3cr1+/+
♂ Rank iCx3cr1Δ/Δ
DAPI Iba1 GnRH
DAPI Iba1 GnRH
Colocalitzacion Microglia - GnRH
Colocalitzacion Microglia - GnRH
50 µm
GnRH Engulfment Microglia
GnRH Engulfment Microglia
Normalized number of microglia-GnRH neuron contacts
0
5
10
15
p = 0.02
♂ Rank iCx3cr1+/+
♂ Rank iCx3cr1Δ/Δ
Normalized number of GnRH particles engulfed by microglia
0
1
2
3
4
5
p = 0.03
♂ Rank iCx3cr1+/+
♂ Rank iCx3cr1Δ/Δ

Fig. S14. Pubertal Rank loss in microglia reduces GnRH-microglia contact in the median eminence. Upper panel**:** Representative IF images of microglial cells (Iba1+, green) and GnRH neurons (GnRH, red) in the median eminence (ME) of control and Rank$^{iCx3cr1\Delta/\Delta}$ mice. Nuclei are labeled with DAPI (blue). Middle panel: Reconstruction of microglia cells using IMARIS in the ME of control and Rank$^{iCx3cr1\Delta/\Delta}$ mice. Colocalization (blue) indicates areas of interaction between microglia and GnRH neurons. Bottom panel: Reconstruction of microglia cells using IMARIS in the ME of control and Rank$^{iCx3cr1\Delta/\Delta}$ mice. Colocalization (pink) indicates areas of GnRH particles engulfed by microglia. Quantification of GnRH neuron-microglia contacts and GnRH particles engulfed by microglia normalized to microglia cell area is provided. Data are represented as mean ± SEM, with each dot representing a mouse; P values were calculated using two-tailed unpaired t-test and exact P values are indicated when statistically significant.

Figure S15

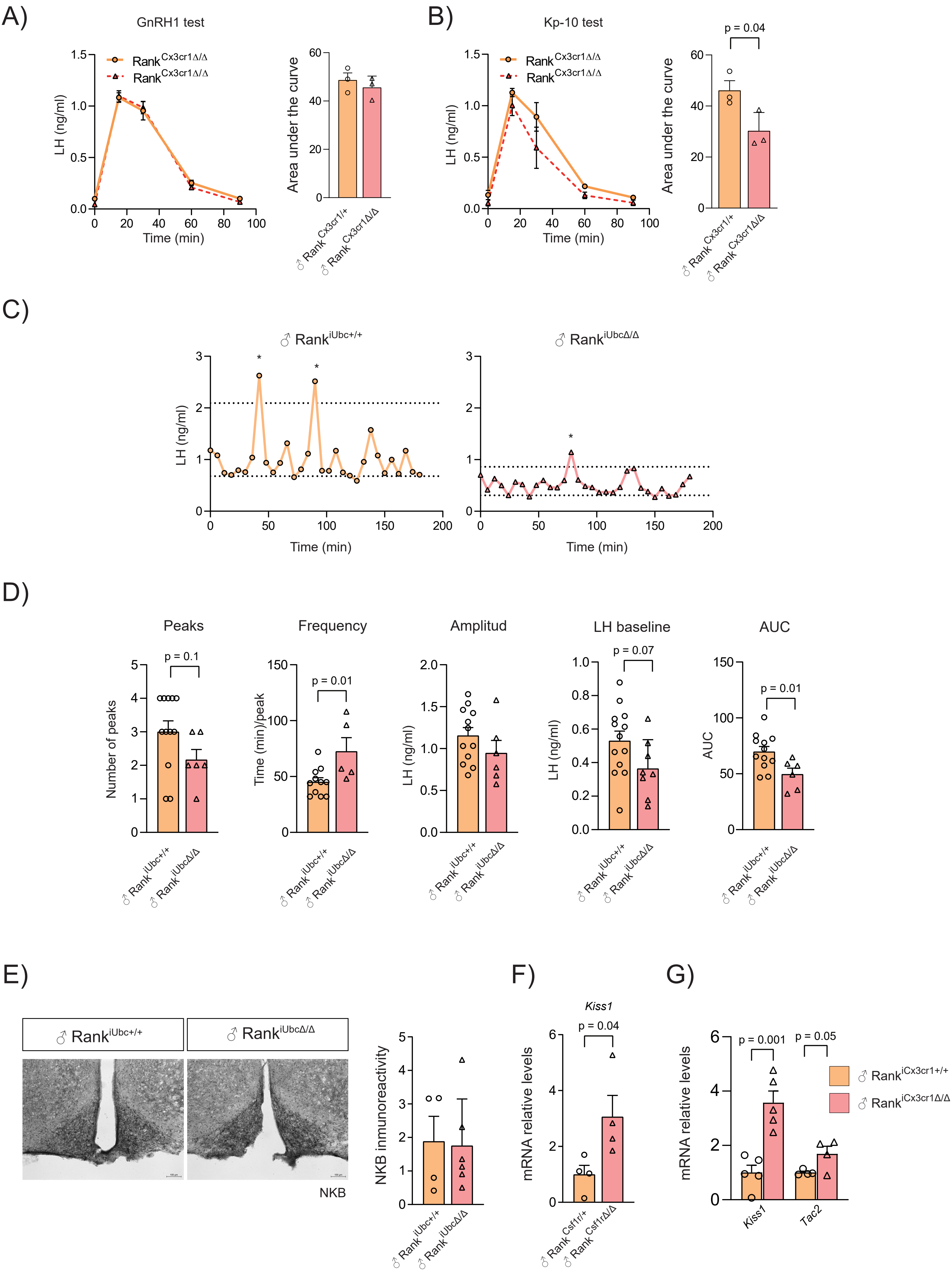
A)
GnRH1 test
Rank$^{Cx3cr1\Delta/\Delta}$
Rank$^{Cx3cr1\Delta/\Delta}$
LH (ng/ml)
Time (min)
Area under the curve
♂ Rank$^{Cx3cr1/+}$
♂ Rank$^{Cx3cr1\Delta/\Delta}$
B)
Kp-10 test
p = 0.04
C)
♂ Rank$^{iUbc+/+}$
♂ Rank$^{iUbc\Delta/\Delta}$
D)
Peaks
Frequency
Amplitud
LH baseline
AUC
p = 0.1
p = 0.01
p = 0.07
p = 0.01
Number of peaks
Time (min)/peak
LH (ng/ml)
AUC
E)
NKB
NKB inmunoreactivity
F)
Kiss1
p = 0.04
mRNA relative levels
♂ Rank$^{Csf1r/+}$
♂ Rank$^{Csf1r\Delta/\Delta}$
G)
p = 0.001
p = 0.05
Kiss1
Tac2
♂ Rank$^{iCx3cr1+/+}$
♂ Rank$^{iCx3cr1\Delta/\Delta}$

Fig. S15. Rank loss in microglia impairs GnRH neuronal response to kisspeptin. (A) Time-course of LH levels in response to GnRH treatment in $Rank^{Cx3cr1\Delta/\Delta}$ (n = 3) and control (n=3) male mice. Right panel shows the quantification of area under the curve (AUC). (B) Time-course LH response to Kisspeptin-10 (Kp-10) in $Rank^{Cx3cr1+/+}$ (n = 3) $Rank^{Cxc3r1\Delta/\Delta}$ (n = 3) male mice. The right panel shows the AUC quantification from the graph on the left panel. (C) Representative graph of time-course of LH levels during 3 hours in $Rank^{iUbc\Delta/\Delta}$ and control male mice. (D) Quantification of LH pulsatility test: number of peaks, interpulse interval, amplitude, LH baseline and AUC. (E) Neurokinin B (*Tac2* gene) immunostaining in the mediobasal hypothalamus of control and $Rank^{iUbc\Delta/\Delta}$ mice. Quantification of the immunoreactive area in the ARC is shown. (F) mRNA amount of *Kiss1* in control and $Rank^{Csf1r\Delta/\Delta}$ hypothalamus. (G) Gene expression of *Kiss1* and *Tac2* in control and $Rank^{iCx3cr1\Delta/\Delta}$ hypothalamus. Data are represented as mean +/-SEM, with each dot representing a mouse; P values were calculated by Unpaired two-tailed t-test and indicated when statistically significant (A-B; D-G). $Rank^{iUbc\Delta/\Delta}$, $Rank^{iCx3cr1\Delta/\Delta}$, and corresponding controls were TAM treated as shown in Fig. 1J and analyses were performed on 8-week-old mice (C-E; G).

Table S1. Gene composition of metagenes and scRNA-seq signatures. This table lists the genes that compose the RANK, GnRH, GHRH, and CRH metagenes, as well as the scRNA-seq-derived cell-type signatures employed throughout the study.

Table S2. Genetic variations in RANK metagene genes in patients with CHH. The table summarizes the genetic analysis of patients with congenital hypogonadotropic hypogonadism. For each patient, genes from the RANK metagene are shown along with the corresponding mutation type (nonsynonymous, stop, insertion, etc.), cDNA changes, protein changes, CADD scores, minor allele frequency (MAF), and Alphamissense scores. Patient sex, inheritance pattern (sporadic or familial), and diagnosis (Kallmann syndrome [KS] or CHH) are also indicated. The table also displays mutations in genes related to CHH.

Table S3. Differential gene expression and pathway enrichment analyses in hypothalamic cell population from Rank$^{iUbc\Delta/\Delta}$ mice obtained by single-cell RNA sequencing. The table contains the differentially expressed genes (DEGs) identified across distinct hypothalamic cell populations (microglia, monocytes, ependymal cells, neutrophils, mural cells, vascular and leptomeningeal cells (VLMCs), endothelial cells, oligodendrocytes, B cells, astrocytes, T cells, and perivascular macrophages (PVMs)) in the comparison Rank$^{iUbc\Delta/\Delta}$ vs Rank$^{iUbc+/+.}$ In addition, gene set enrichment analysis for microglia is shown, based on Hallmark, Reactome, and KEGG gene sets.

Table S4. Differential gene expression and pathway enrichment analyses across the four microglial subpopulations identified in the hypothalamus by single-cell RNA sequencing. For these analyses, data from Rank$^{iUbc\Delta/\Delta}$ and Rank$^{iUbc+/+}$ mice were integrated. The table presents the differentially expressed genes (DEGs) for each microglial cluster, obtained by comparing each subpopulation against the global microglia population, together with the corresponding gene set enrichment analysis based on Hallmark, Reactome, and KEGG gene sets. Microglial population 4 was excluded from downstream analyses because it did not pass quality control criteria.

Table S5. Differential gene expression and pathway enrichment analyses for each microglial population from Rank$^{iUbc\Delta/\Delta}$ mice in the hypothalamus. The table lists the differentially expressed genes (DEGs) for each microglial cluster in the direct comparison between Rank$^{iUbc\Delta/\Delta}$ and Rank$^{iUbc+/+}$ mice, along with the corresponding gene set enrichment analysis (GSEA) results based on Hallmark, Reactome, and KEGG gene sets, reported separately for each microglial population

Table S6. Primers used for genotyping and qPCR. List of primer sequences used for mouse genotyping and quantitative real-time PCR (qPCR) analyses. The qPCR primers include the gene name and forward (F) and reverse (R) sequences (5′–3′). The genotyping primers include two or more primers required for accurate mouse genotyping.

Table S7. Mendelian frequencies of Rank ex1$^{-/-}$ mice. Observed and expected Mendelian genotype frequencies from Rank ex1 heterozygous matings. The table includes the number of mice obtained for each genotype, the expected number according to Mendelian inheritance (1:2:1 ratio), and the corresponding expected percentages. Total number of analyzed mice is indicated.

Movie S1. Three-dimensional reconstruction of microglia in the median eminence of Rank$^{iUbc+/+}$ mice. Representative video of the median eminence from control mice treated as indicated in Fig 1J. Nuclei are labeled with DAPI (blue) and microglia are labeled in green. The movie shows a three-dimensional (3D) reconstruction highlighting microglial morphology, including the soma and cellular processes.

Movie S2. Three-dimensional reconstruction of microglia in the median eminence of Rank$^{iUbc\Delta/\Delta}$ mice. Representative video of the median eminence from Rank$^{iUbc\Delta/\Delta}$ mice upon

pubertal RANK loss treated as indicated in Fig 1J. Nuclei are labeled with DAPI (blue) and microglia are labeled in green. The movie shows a three-dimensional (3D) reconstruction highlighting microglial morphology, including the soma and cellular processes.

Movie S3. GnRH–microglia interactions in the median eminence of Rank$^{iUbc+/+}$ animals. 3D reconstruction of the median eminence from control mice treated as indicated in Fig 1J. GnRH neuronal terminals are shown in red, microglia in green, and an example of a microglia cell are shown. Regions of close GnRH–microglia interactions are indicated in blue.

Movie S4. GnRH–microglia interactions in the median eminence of Rank$^{iUbc\Delta/\Delta}$ animals. 3D reconstruction of the median eminence from Rank$^{iUbc\Delta/\Delta}$ mice upon pubertal RANK loss treated as indicated in Fig 1J. GnRH neuronal terminals are shown in red, microglia in green, and an example of a microglia cell are shown. Regions of close GnRH–microglia interactions are indicated in blue.

References and Notes

1. J. E. Fata, Y. Y. Kong, J. Li, T. Sasaki, J. Irie-Sasaki, R. A. Moorehead, R. Elliott, S. Scully, E. B. Voura, D. L. Lacey, W. J. Boyle, R. Khokha, J. M. Penninger, The osteoclast differentiation factor osteoprotegerin-ligand is essential for mammary gland development. *Cell* **103**, 41–50 (2000). doi:10.1016/S0092-8674(00)00103-3 Medline

2. M. Beleut, R. D. Rajaram, M. Caikovski, A. Ayyanan, D. Germano, Y. Choi, P. Schneider, C. Brisken, Two distinct mechanisms underlie progesterone-induced proliferation in the mammary gland. *Proc. Natl. Acad. Sci. U.S.A.* **107**, 2989–2994 (2010). doi:10.1073/pnas.0915148107 Medline

3. T. Tanos, G. Sflomos, P. C. Echeverria, A. Ayyanan, M. Gutierrez, J.-F. Delaloye, W. Raffoul, M. Fiche, W. Dougall, P. Schneider, O. Yalcin-Ozuysal, C. Brisken, Progesterone/RANKL is a major regulatory axis in the human breast. *Sci. Transl. Med.* **5**, 182ra55 (2013). doi:10.1126/scitranslmed.3005654 Medline

4. A. Cordero, P. Pellegrini, A. Sanz-Moreno, E. M. Trinidad, J. Serra-Musach, C. Deshpande, W. C. Dougall, M. A. Pujana, E. González-Suárez, Rankl Impairs Lactogenic Differentiation Through Inhibition of the Prolactin/Stat5 Pathway at Midgestation. *Stem Cells* **34**, 1027–1039 (2016). doi:10.1002/stem.2271 Medline

5. B. L. Riggs, The mechanisms of estrogen regulation of bone resorption. *J. Clin. Invest.* **106**, 1203–1204 (2000). doi:10.1172/JCI11468 Medline

6. B. Lunenfeld, K. Bühler, The neuro control of the ovarain cycle - a hypothesis. *Gynecol. Endocrinol.* **34**, 278–282 (2018). doi:10.1080/09513590.2017.1405933 Medline

7. A. E. Herbison, Control of puberty onset and fertility by gonadotropin-releasing hormone neurons. *Nat. Rev. Endocrinol.* **12**, 452–466 (2016). doi:10.1038/nrendo.2016.70 Medline

8. L. Pinilla, E. Aguilar, C. Dieguez, R. P. Millar, M. Tena-Sempere, Kisspeptins and reproduction: Physiological roles and regulatory mechanisms. *Physiol. Rev.* **92**, 1235–1316 (2012). doi:10.1152/physrev.00037.2010 Medline

9. W. C. Dougall, M. Glaccum, K. Charrier, K. Rohrbach, K. Brasel, T. De Smedt, E. Daro, J. Smith, M. E. Tometsko, C. R. Maliszewski, A. Armstrong, V. Shen, S. Bain, D. Cosman, D. Anderson, P. J. Morrissey, J. J. Peschon, J. Schuh, RANK is essential for osteoclast and lymph node development. *Genes Dev.* **13**, 2412–2424 (1999). doi:10.1101/gad.13.18.2412 Medline

10. A. S. Rocha, A. Collado-Solé, O. Graña-Castro, J. Redondo-Pedraza, G. Soria-Alcaide, A. Cordero, P. G. Santamaría, E. González-Suárez, Luminal Rank loss decreases cell fitness leading to basal cell bipotency in parous mammary glands. *Nat. Commun.* **14**, 6213 (2023). doi:10.1038/s41467-023-41741-5 Medline

11. C. W. Daniel, G. B. Silberstein, P. Strickland, Direct action of 17 β-estradiol on mouse mammary ducts analyzed by sustained release implants and steroid autoradiography. *Cancer Res.* **47**, 6052–6057 (1987). Medline

12. S. A. Best, K. J. Hutt, N. Y. Fu, F. Vaillant, S. H. Liew, L. Hartley, C. L. Scott, G. J. Lindeman, J. E. Visvader, Dual roles for Id4 in the regulation of estrogen signaling in the mammary gland and ovary. *Development* **141**, 3159–3164 (2014). doi:10.1242/dev.108498 Medline

13. G. Fink, Oestrogen and progesterone interactions in the control of gonadotrophin and prolactin secretion. *J. Steroid Biochem.* **30**, 169–178 (1988). doi:10.1016/0022-4731(88)90090-8 Medline

14. B. J. Strober, X. Wen, V. Wucher, A. Kwong, T. Lappalainen, X. Li, Y. Liang, The GTEx Consortium atlas of genetic regulatory effects across human tissues. *Man. Muñoz-Aguirre* **18**, 1318–1330 (2020).

15. D. Cassatella, S. R. Howard, J. S. Acierno, C. Xu, G. E. Papadakis, F. A. Santoni, A. A. Dwyer, S. Santini, G. P. Sykiotis, C. Chambion, J. Meylan, L. Marino, L. Favre, J. Li, X. Liu, J. Zhang, P. M. Bouloux, C. Geyter, A. Paepe, W. S. Dhillo, J. M. Ferrara, M. Hauschild, M. Lang-Muritano, J. R. Lemke, C. Flück, A. Nemeth, F. Phan-Hug, D. Pignatelli, V. Popovic, S. Pekic, R. Quinton, G. Szinnai, D. l'Allemand, D. Konrad, S. Sharif, Ö. T. Iyidir, B. J. Stevenson, H. Yang, L. Dunkel, N. Pitteloud, Congenital hypogonadotropic hypogonadism and constitutional delay of growth and puberty have distinct genetic architectures. *Eur. J. Endocrinol.* **178**, 377–388 (2018). doi:10.1530/EJE-17-0568 Medline

16. K. J. Karczewski, L. C. Francioli, G. Tiao, B. B. Cummings, J. Alföldi, Q. Wang, R. L. Collins, K. M. Laricchia, A. Ganna, D. P. Birnbaum, L. D. Gauthier, H. Brand, M. Solomonson, N. A. Watts, D. Rhodes, M. Singer-Berk, E. M. England, E. G. Seaby, J. A. Kosmicki, R. K. Walters, K. Tashman, Y. Farjoun, E. Banks, T. Poterba, A. Wang, C. Seed, N. Whiffin, J. X. Chong, K. E. Samocha, E. Pierce-Hoffman, Z. Zappala, A. H. O'Donnell-Luria, E. V. Minikel, B. Weisburd, M. Lek, J. S. Ware, C. Vittal, I. M. Armean, L. Bergelson, K. Cibulskis, K. M. Connolly, M. Covarrubias, S. Donnelly, S. Ferriera, S. Gabriel, J. Gentry, N. Gupta, T. Jeandet, D. Kaplan, C. Llanwarne, R. Munshi, S. Novod, N. Petrillo, D. Roazen, V. Ruano-Rubio, A. Saltzman, M. Schleicher, J. Soto, K. Tibbetts, C. Tolonen, G. Wade, M. E. Talkowski, C. A. Aguilar Salinas, T. Ahmad, C. M. Albert, D. Ardissino, G. Atzmon, J. Barnard, L. Beaugerie, E. J. Benjamin, M. Boehnke, L. L. Bonnycastle, E. P. Bottinger, D. W. Bowden, M. J. Bown, J. C. Chambers, J. C. Chan, D. Chasman, J. Cho, M. K. Chung, B. Cohen, A. Correa, D. Dabelea, M. J. Daly, D. Darbar, R. Duggirala, J. Dupuis, P. T. Ellinor, R. Elosua, J. Erdmann, T. Esko, M. Färkkilä, J. Florez, A. Franke, G. Getz, B. Glaser, S. J. Glatt, D. Goldstein, C. Gonzalez, L. Groop, C. Haiman, C. Hanis, M. Harms, M. Hiltunen, M. M. Holi, C. M. Hultman, M. Kallela, J. Kaprio, S. Kathiresan, B. J. Kim, Y. J. Kim, G. Kirov, J. Kooner, S. Koskinen, H. M. Krumholz, S. Kugathasan, S. H. Kwak, M. Laakso, T. Lehtimäki, R. J. F. Loos, S. A. Lubitz, R. C. W. Ma, D. G. MacArthur, J. Marrugat, K. M. Mattila, S. McCarroll, M. I. McCarthy, D. McGovern, R. McPherson, J. B. Meigs, O. Melander, A. Metspalu, B. M. Neale, P. M. Nilsson, M. C. O'Donovan, D. Ongur, L. Orozco, M. J. Owen, C. N. A. Palmer, A. Palotie, K. S. Park, C. Pato, A. E. Pulver, N. Rahman, A. M. Remes, J. D. Rioux, S. Ripatti, D. M. Roden, D. Saleheen, V. Salomaa, N. J. Samani, J. Scharf, H. Schunkert, M. B. Shoemaker, P. Sklar, H. Soininen, H. Sokol, T. Spector, P. F. Sullivan, J. Suvisaari, E. S. Tai, Y. Y. Teo, T. Tiinamaija, M. Tsuang, D. Turner, T. Tusie-Luna, E. Vartiainen, H. Watkins, R. K. Weersma, M. Wessman, J. G. Wilson, R. J. Xavier, The mutational constraint spectrum quantified from variation in 141,456 humans. *Nature* **581**, 434–443 (2020). doi:10.1038/s41586-020-2308-7

17. S. H. Kim, Congenital hypogonadotropic hypogonadism and Kallmann syndrome: Past, present, and future. *Endocrinol. Metab. (Seoul)* **30**, 456–466 (2015). doi:10.3803/EnM.2015.30.4.456 Medline

18. N. M. Petersen, A. L. Briggs, Selective estrogen receptor modulators. *Clin. Rev. Bone Miner. Metab.* **3**, 19–30 (2005). doi:10.1385/BMM:3:1:019

19. M. Shehata, R. van Amerongen, A. L. Zeeman, R. R. Giraddi, J. Stingl, The influence of tamoxifen on normal mouse mammary gland homeostasis. *Breast Cancer Res.* **16**, 411 (2014). doi:10.1186/s13058-014-0411-0 Medline

20. L. Steuernagel, B. Y. H. Lam, P. Klemm, G. K. C. Dowsett, C. A. Bauder, J. A. Tadross, T. S. Hitschfeld, A. Del Rio Martin, W. Chen, A. J. de Solis, H. Fenselau, P. Davidsen, I. Cimino, S. N. Kohnke, D. Rimmington, A. P. Coll, A. Beyer, G. S. H. Yeo, J. C. Brüning, HypoMap-a unified single-cell gene expression atlas of the murine hypothalamus. *Nat. Metab.* **4**, 1402–1419 (2022). doi:10.1038/s42255-022-00657-y Medline

21. X. Zhou, Y. Lu, F. Zhao, J. Dong, W. Ma, S. Zhong, M. Wang, B. Wang, Y. Zhao, Y. Shi, Q. Ma, T. Lu, J. Zhang, X. Wang, Q. Wu, Deciphering the spatial-temporal transcriptional landscape of human hypothalamus development. *Cell Stem Cell* **29**, 328–343.e5 (2022). doi:10.1016/j.stem.2021.11.009 Medline

22. J. A. Tadross, L. Steuernagel, G. K. C. Dowsett, K. A. Kentistou, S. Lundh, M. Porniece, P. Klemm, K. Rainbow, H. Hvid, K. Kania, J. Polex-Wolf, L. B. Knudsen, C. Pyke, J. R. B. Perry, B. Y. H. Lam, J. C. Brüning, G. S. H. Yeo, A comprehensive spatio-cellular map of the human hypothalamus. *Nature* **639**, 708–716 (2025). doi:10.1038/s41586-024-08504-8 Medline

23. O. Franzén, L. M. Gan, J. L. M. Björkegren, D. B. Panglao, PanglaoDB: A web server for exploration of mouse and human single-cell RNA sequencing data. *Database* **2019**, 1–9 (2019). doi:10.1093/database/baz046 Medline

24. C. L. Abram, G. L. Roberge, Y. Hu, C. A. Lowell, Comparative analysis of the efficiency and specificity of myeloid-Cre deleting strains using ROSA-EYFP reporter mice. *J. Immunol. Methods* **408**, 89–100 (2014). doi:10.1016/j.jim.2014.05.009

25. R. E. Henn, K. Guo, S. E. Elzinga, M. H. Noureldein, F. E. Mendelson, J. M. Hayes, D. M. Rigan, M. G. Savelieff, J. Hur, E. L. Feldman, Single-cell RNA sequencing identifies hippocampal microglial dysregulation in diet-induced obesity. *iScience* **26**, 106164 (2023). doi:10.1016/j.isci.2023.106164 Medline

26. J. N. Campbell, E. Z. Macosko, H. Fenselau, A. Lyubetskaya, D. Tenen, M. Goldman, A. M. J. Verstegen, J. M. Resch, S. A. Mccarroll, E. D. Rosen, B. B. Lowell, L. T. Tsai, A molecular census of arcuate hypothalamus and median eminence cell types. *Nat. Neurosci.* **20**, 484–496 (2017). doi:10.1038/nn.4495

27. R. Chen, X. Wu, L. Jiang, Y. Zhang, Single-Cell RNA-Seq Reveals Hypothalamic Cell Diversity. *Cell Rep.* **18**, 3227–3241 (2017). doi:10.1016/j.celrep.2017.03.004 Medline

28. T. R. Hammond, C. Dufort, L. Dissing-Olesen, S. Giera, A. Young, A. Wysoker, A. J. Walker, F. Gergits, M. Segel, J. Nemesh, S. E. Marsh, A. Saunders, E. Macosko, F. Ginhoux, J. Chen, R. J. M. Franklin, X. Piao, S. A. McCarroll, B. Stevens, Single cell RNA sequencing of microglia throughout the mouse lifespan and in the injured brain reveals complex cell-state changes. *Immunity* **50**, 253–271.e6 (2019). doi:10.1016/j.immuni.2018.11.004 Medline

29. C. Sousa, A. Golebiewska, S. K. Poovathingal, T. Kaoma, Y. Pires-Afonso, S. Martina, D. Coowar, F. Azuaje, A. Skupin, R. Balling, K. Biber, S. P. Niclou, A. Michelucci, Single-cell transcriptomics reveals distinct inflammation-induced microglia signatures. *EMBO Rep.* **19**, e46171 (2018). doi:10.15252/embr.201846171 Medline

30. O. Matcovitch-Natan, D. R. Winter, A. Giladi, S. Vargas Aguilar, A. Spinrad, S. Sarrazin, H. Ben-Yehuda, E. David, F. Zelada González, P. Perrin, H. Keren-Shaul, M. Gury, D. Lara-Astaiso, C. A. Thaiss, M. Cohen, K. Bahar Halpern, K. Baruch, A. Deczkowska, E. Lorenzo-Vivas, S. Itzkovitz, E. Elinav, M. H. Sieweke, M. Schwartz, I. Amit, Microglia development follows a stepwise program to regulate brain homeostasis. *Science* **353**, aad8670 (2016). doi:10.1126/science.aad8670 Medline

31. O. Butovsky, M. P. Jedrychowski, C. S. Moore, R. Cialic, A. J. Lanser, G. Gabriely, T. Koeglsperger, B. Dake, P. M. Wu, C. E. Doykan, Z. Fanek, L. Liu, Z. Chen, J. D. Rothstein, R. M. Ransohoff, S. P. Gygi, J. P. Antel, H. L. Weiner, Identification of a unique TGF-β-dependent molecular and functional signature in microglia. *Nat. Neurosci.* **17**, 131–143 (2014). doi:10.1038/nn.3599 Medline

32. A. E. Ayoub, A. K. Salm, Increased morphological diversity of microglia in the activated hypothalamic supraoptic nucleus. *J. Neurosci.* **23**, 7759–7766 (2003). doi:10.1523/JNEUROSCI.23-21-07759.2003 Medline

33. J. R. Moffitt, D. Bambah-Mukku, S. W. Eichhorn, E. Vaughn, K. Shekhar, J. D. Perez, N. D. Rubinstein, J. Hao, A. Regev, C. Dulac, X. Zhuang, Molecular, spatial, and functional single-cell profiling of the hypothalamic preoptic region. *Science* **362**, eaau5324 (2018). doi:10.1126/science.aau5324 Medline

34. S. Kohnke, S. Buller, D. Nuzzaci, K. Ridley, B. Lam, H. Pivonkova, M. A. Bentsen, K. M. Alonge, C. Zhao, J. Tadross, S. Holmqvist, T. Shimizu, H. Hathaway, H. Li, W. Macklin, M. W. Schwartz, W. D. Richardson, G. S. H. Yeo, R. J. M. Franklin, R. T. Karadottir, D. H. Rowitch, C. Blouet, Nutritional regulation of oligodendrocyte differentiation regulates perineuronal net remodeling in the median eminence. *Cell Rep.* **36**, 109362 (2021). doi:10.1016/j.celrep.2021.109362 Medline

35. T. L. Stincic, J. Qiu, A. M. Connors, M. J. Kelly, O. K. Rønnekleiv, Arcuate and Preoptic Kisspeptin Neurons Exhibit Differential Projections to Hypothalamic Nuclei and Exert Opposite Postsynaptic Effects on Hypothalamic Paraventricular and Dorsomedial Nuclei in the Female Mouse. *eNeuro* **8**, ENEURO.0093-21.2021 (2021). doi:10.1523/ENEURO.0093-21.2021 Medline

36. D. G. de Paula, T. M. Bohlen, T. T. Zampieri, N. S. Mansano, H. R. Vieira, D. O. Gusmao, F. Wasinski, J. Donato Jr., R. Frazao, Distinct effects of growth hormone deficiency and disruption of hypothalamic kisspeptin system on reproduction of male mice. *Life Sci.* **285**, 119970 (2021). doi:10.1016/j.lfs.2021.119970 Medline

37. M. Manfredi-Lozano, J. Roa, M. Tena-Sempere, Connecting metabolism and gonadal function: Novel central neuropeptide pathways involved in the metabolic control of puberty and fertility. *Front. Neuroendocrinol.* **48**, 37–49 (2018). doi:10.1016/j.yfrne.2017.07.008 Medline

38. M. Blomberg Jensen, C. H. Andreassen, A. Jørgensen, J. E. Nielsen, L. Juel Mortensen, I. M. Boisen, P. Schwarz, J. Toppari, R. Baron, B. Lanske, A. Juul, RANKL regulates male

reproductive function. *Nat. Commun.* **12**, 2450 (2021). doi:10.1038/s41467-021-22734-8 Medline

39. Y. Lu, P. Liu, R. R. Recker, H. W. Deng, V. Dvornyk, TNFRSF11A and TNFSF11 are associated with age at menarche and natural menopause in white women. *Menopause* **17**, 1048–1054 (2010). doi:10.1097/gme.0b013e3181d5d523 Medline

40. R. Pan, Y. Z. Liu, H. W. Deng, V. Dvornyk, Association analyses suggest the effects of RANK and RANKL on age at menarche in Chinese women. *Climacteric* **15**, 75–81 (2012). doi:10.3109/13697137.2011.587556 Medline

41. P. Tu, P. Duan, R. S. Zhang, D. B. Xu, Y. Wang, H. P. Wu, Y. H. Liu, L. Si, Polymorphisms in genes in the RANKL/RANK/OPG pathway are associated with bone mineral density at different skeletal sites in post-menopausal women. *Osteoporos. Int.* **26**, 179–185 (2015). doi:10.1007/s00198-014-2854-7 Medline

42. A. S. Kauffman, V. M. Navarro, J. Kim, D. K. Clifton, R. A. Steiner, Sex differences in the regulation of Kiss1/NKB neurons in juvenile mice: Implications for the timing of puberty. *Am. J. Physiol. Endocrinol. Metab.* **297**, E1212–E1221 (2009). doi:10.1152/ajpendo.00461.2009 Medline

43. S. Kang, E. Y. Ko, A. E. Andrews, J. E. Shin, K. J. Nance, P. K. Barman, P. S. Heeger, W. M. Freeman, B. A. Benayoun, H. S. Goodridge, Microglia undergo sex-dimorphic transcriptional and metabolic rewiring during aging. *J. Neuroinflammation* **21**, 150 (2024). doi:10.1186/s12974-024-03130-7 Medline

44. J. E. Sánchez-Criado, P. Guelmes, C. Bellido, M. Gónzalez, G. Hernández, R. Aguilar, J. C. Garrido-Gracia, A. R. Bello, R. Alonso, Tamoxifen but not other selective estrogen receptor modulators antagonizes estrogen actions on luteinizing hormone secretion while inducing gonadotropin-releasing hormone self-priming in the rat. *Neuroendocrinology* **76**, 203–213 (2002). doi:10.1159/000065952 Medline

45. R. Verma, A. Krishna, Effect of tamoxifen on spermatogenesis and testicular steroidogenesis. *Biochem. Biophys. Res. Commun.* **486**, 36–42 (2017). doi:10.1016/j.bbrc.2017.02.092 Medline

46. S. H. Patel, L. O'Hara, N. Atanassova, S. E. Smith, M. K. Curley, D. Rebourcet, A. L. Darbey, A.-L. Gannon, R. M. Sharpe, L. B. Smith, Low-dose tamoxifen treatment in juvenile males has long-term adverse effects on the reproductive system: Implications for inducible transgenics. *Sci. Rep.* **7**, 8991 (2017). doi:10.1038/s41598-017-09016-4 Medline

47. N. de Roux, E. Genin, J. C. Carel, F. Matsuda, J. L. Chaussain, E. Milgrom, Hypogonadotropic hypogonadism due to loss of function of the KiSS1-derived peptide receptor GPR54. *Proc. Natl. Acad. Sci. U.S.A.* **100**, 10972–10976 (2003). doi:10.1073/pnas.1834399100 Medline

48. S. B. Seminara, S. Messager, E. E. Chatzidaki, R. R. Thresher, J. S. Acierno Jr., J. K. Shagoury, Y. Bo-Abbas, W. Kuohung, K. M. Schwinof, A. G. Hendrick, D. Zahn, J. Dixon, U. B. Kaiser, S. A. Slaugenhaupt, J. F. Gusella, S. O'Rahilly, M. B. L. Carlton, W. F. Crowley Jr., S. A. J. R. Aparicio, W. H. Colledge, The GPR54 Gene as a Regulator of Puberty. *Obstet. Gynecol. Surv.* **59**, 351–353 (2004). doi:10.1097/00006254-200405000-00020

49. C. E. Roselli, R. Amodei, K. P. Gribbin, K. Corder, F. Stormshak, C. T. Estill, Excess Testosterone Exposure Alters Hypothalamic-Pituitary-Testicular Axis Dynamics and Gene Expression in Sheep Fetuses. *Endocrinology* **157**, 4234–4245 (2016). doi:10.1210/en.2016-1411 Medline

50. Y. Uenoyama, N. Inoue, S. Nakamura, H. Tsukamura, Kisspeptin Neurons and Estrogen-Estrogen Receptor α Signaling: Unraveling the Mystery of Steroid Feedback System Regulating Mammalian Reproduction. *Int. J. Mol. Sci.* **22**, 9229 (2021). doi:10.3390/ijms22179229 Medline

51. R. Oleari, V. Massa, A. Cariboni, A. Lettieri, The Differential Roles for Neurodevelopmental and Neuroendocrine Genes in Shaping GnRH Neuron Physiology and Deficiency. *Int. J. Mol. Sci.* **22**, 9425 (2021). doi:10.3390/ijms22179425 Medline

52. Q. Xie, Y. Kang, C. Zhang, Y. Xie, C. Wang, J. Liu, The Role of Kisspeptin in the Control of the Hypothalamic-Pituitary- Gonadal Axis and Reproduction. *Front. Endocrinol.* **13**, 925206 (2022).

53. V. Prevot, B. Dehouck, A. Sharif, P. Ciofi, P. Giacobini, J. Clasadonte, The versatile tanycyte: A hypothalamic integrator of reproduction and energy metabolism. *Endocr. Rev.* **39**, 333–368 (2018). doi:10.1210/er.2017-00235 Medline

54. G. Pellegrino, M. Martin, C. Allet, T. Lhomme, S. Geller, D. Franssen, V. Mansuy, M. Manfredi-Lozano, A. Coutteau-Robles, V. Delli, S. Rasika, D. Mazur, A. Loyens, M. Tena-Sempere, J. Siepmann, F. P. Pralong, P. Ciofi, G. Corfas, A. S. Parent, S. R. Ojeda, A. Sharif, V. Prevot, GnRH neurons recruit astrocytes in infancy to facilitate network integration and sexual maturation. *Nat. Neurosci.* **24**, 1660–1672 (2021). doi:10.1038/s41593-021-00960-z Medline

55. A. Sharif, M. Baroncini, V. Prevot, Role of glia in the regulation of gonadotropin-releasing hormone neuronal activity and secretion. *Neuroendocrinology* **98**, 1–15 (2013). doi:10.1159/000351867 Medline

56. B. Erblich, L. Zhu, A. M. Etgen, K. Dobrenis, J. W. Pollard, Absence of colony stimulation factor-1 receptor results in loss of microglia, disrupted brain development and olfactory deficits. *PLOS ONE* **6**, e26317 (2011). doi:10.1371/journal.pone.0026317 Medline

57. N. M. Lainez, C. R. Jonak, M. G. Nair, I. M. Ethell, E. H. Wilson, M. J. Carson, D. Coss, Diet-induced obesity elicits macrophage infiltration and reduction in spine density in the hypothalami of male but not female mice. *Front. Immunol.* **9**, 1992 (2018). doi:10.3389/fimmu.2018.01992 Medline

58. P. E. Cohen, L. Zhu, K. Nishimura, J. W. Pollard, Colony-stimulating factor 1 regulation of neuroendocrine pathways that control gonadal function in mice. *Endocrinology* **143**, 1413–1422 (2002). doi:10.1210/endo.143.4.8754 Medline

59. Q. Li, B. A. Barres, Microglia and macrophages in brain homeostasis and disease. *Nat. Rev. Immunol.* **18**, 225–242 (2018). doi:10.1038/nri.2017.125 Medline

60. L. Muzio, A. Viotti, G. Martino, Microglia in Neuroinflammation and Neurodegeneration: From Understanding to Therapy. *Front. Neurosci.* **15**, 742065 (2021). doi:10.3389/fnins.2021.742065 Medline

61. M. Augusto-Oliveira, G. P. Arrifano, A. Lopes-Araújo, L. Santos-Sacramento, P. Y. Takeda, D. C. Anthony, J. O. Malva, M. E. Crespo-Lopez, What do microglia really do in healthy adult brain? *Cells* **8**, 1293 (2019). doi:10.3390/cells8101293 Medline

62. M. Shimamura, H. Nakagami, M. K. Osako, H. Kurinami, H. Koriyama, P. Zhengda, H. Tomioka, A. Tenma, K. Wakayama, R. Morishita, OPG/RANKL/RANK axis is a critical inflammatory signaling system in ischemic brain in mice. *Proc. Natl. Acad. Sci. U.S.A.* **111**, 8191–8196 (2014). doi:10.1073/pnas.1400544111 Medline

63. A. Glasnović, N. O'Mara, N. Kovačić, D. Grčević, S. Gajović, RANK/RANKL/OPG Signaling in the Brain: A Systematic Review of the Literature. *Front. Neurol.* **11**, 590480 (2020). doi:10.3389/fneur.2020.590480 Medline

64. A. Kichev, P. Eede, P. Gressens, C. Thornton, H. Hagberg, Implicating Receptor Activator of NF-κB (RANK)/RANK Ligand Signalling in Microglial Responses to Toll-Like Receptor Stimuli. *Dev. Neurosci.* **39**, 192–206 (2017). doi:10.1159/000464244 Medline

65. L. Keane, I. Antignano, S. P. Riechers, R. Zollinger, A. A. Dumas, N. Offermann, M. E. Bernis, J. Russ, F. Graelmann, P. N. McCormick, J. Esser, D. Tejera, A. Nagano, J. Wang, C. Chelala, Y. Biederbick, A. Halle, P. Salomoni, M. T. Heneka, M. Capasso, mTOR-dependent translation amplifies microglia priming in aging mice. *J. Clin. Invest.* **131**, e155208 (2021). doi:10.1172/JCI155208 Medline

66. Z. X. Xu, G. H. Kim, J. W. Tan, A. E. Riso, Y. Sun, E. Y. Xu, G. Y. Liao, H. Xu, S. H. Lee, N. Y. Do, C. H. Lee, A. E. Clipperton-Allen, S. Kwon, D. T. Page, K. J. Lee, B. Xu, Elevated protein synthesis in microglia causes autism-like synaptic and behavioral aberrations. *Nat. Commun.* **11**, 1797 (2020). doi:10.1038/s41467-020-15530-3 Medline

67. A. Vidal-Itriago, R. A. W. Radford, J. A. Aramideh, C. Maurel, N. M. Scherer, E. K. Don, A. Lee, R. S. Chung, M. B. Graeber, M. Morsch, Microglia morphophysiological diversity and its implications for the CNS. *Front. Immunol.* **13**, 997786 (2022). doi:10.3389/fimmu.2022.997786 Medline

68. B. Basilico, L. Ferrucci, P. Ratano, M. T. Golia, A. Grimaldi, M. Rosito, V. Ferretti, I. Reverte, C. Sanchini, M. C. Marrone, M. Giubettini, V. De Turris, D. Salerno, S. Garofalo, M. K. St-Pierre, M. Carrier, M. Renzi, F. Pagani, B. Modi, M. Raspa, F. Scavizzi, C. T. Gross, S. Marinelli, M. È. Tremblay, D. Caprioli, L. Maggi, C. Limatola, S. Di Angelantonio, D. Ragozzino, Microglia control glutamatergic synapses in the adult mouse hippocampus. *Glia* **70**, 173–195 (2022). doi:10.1002/glia.24101 Medline

69. U. Boehm, P. M. Bouloux, M. T. Dattani, N. de Roux, C. Dodé, L. Dunkel, A. A. Dwyer, P. Giacobini, J. P. Hardelin, A. Juul, M. Maghnie, N. Pitteloud, V. Prevot, T. Raivio, M. Tena-Sempere, R. Quinton, J. Young, European Consensus Statement on congenital hypogonadotropic hypogonadism—pathogenesis, diagnosis and treatment. *Nat. Rev. Endocrinol.* **11**, 547–564 (2015). doi:10.1038/nrendo.2015.112 Medline

70. H. M. Lewkowitz-Shpuntoff, V. A. Hughes, L. Plummer, M. G. Au, R. L. Doty, S. B. Seminara, Y. M. Chan, N. Pitteloud, W. F. Crowley Jr., R. Balasubramanian, Olfactory phenotypic spectrum in idiopathic hypogonadotropic hypogonadism: Pathophysiological and genetic implications. *J. Clin. Endocrinol. Metab.* **97**, E136–E144 (2012). doi:10.1210/jc.2011-2041 Medline

71. H. Li, R. Durbin, Fast and accurate short read alignment with Burrows-Wheeler transform. *Bioinformatics* **25**, 1754–1760 (2009). doi:10.1093/bioinformatics/btp324 Medline

72. M. A. Depristo, E. Banks, R. E. Poplin, K. V. Garimella, J. R. Maguire, C. Hartl, M. A. Rivas, M. Hanna, A. Mckenna, T. J. Fennell, A. Y. Sivachenko, K. Cibulskis, S. B. Gabriel, D. Altshuler, P. Genetics, M. G. Hospital, R. B. Simches, A framework for variation discovery and genotyping using nextgeneration DNA sequencing data. *Nat. Genet.* **43**, 491–498 (2011). doi:10.1038/ng.806

73. K. Wang, M. Li, H. Hakonarson, ANNOVAR: Functional annotation of genetic variants from high-throughput sequencing data. *Nucleic Acids Res.* **38**, e164 (2010). doi:10.1093/nar/gkq603 Medline

74. P. Rentzsch, M. Schubach, J. Shendure, M. Kircher, CADD-Splice-improving genome-wide variant effect prediction using deep learning-derived splice scores. *Genome Med.* **13**, 31 (2021). doi:10.1186/s13073-021-00835-9 Medline

75. J. Cheng, G. Novati, J. Pan, C. Bycroft, A. Žemgulytė, T. Applebaum, A. Pritzel, L. H. Wong, M. Zielinski, T. Sargeant, R. G. Schneider, A. W. Senior, J. Jumper, D. Hassabis, P. Kohli, Ž. Avsec, Accurate proteome-wide missense variant effect prediction with AlphaMissense. *Science* **381**, eadg7492 (2023). doi:10.1126/science.adg7492 Medline

76. A. Santani, J. Murrell, B. Funke, Z. Yu, M. Hegde, R. Mao, A. Ferreira-Gonzalez, K. V. Voelkerding, K. E. Weck, Development and validation of targeted next-generation sequencing panels for detection of germline variants in inherited diseases. *Arch. Pathol. Lab. Med.* **141**, 787–797 (2017). doi:10.5858/arpa.2016-0517-RA Medline

77. R. Hanada, A. Leibbrandt, T. Hanada, S. Kitaoka, T. Furuyashiki, H. Fujihara, J. Trichereau, M. Paolino, F. Qadri, R. Plehm, S. Klaere, V. Komnenovic, H. Mimata, H. Yoshimatsu, N. Takahashi, A. von Haeseler, M. Bader, S. S. Kilic, Y. Ueta, C. Pifl, S. Narumiya, J. M. Penninger, Central control of fever and female body temperature by RANKL/RANK. *Nature* **462**, 505–509 (2009). doi:10.1038/nature08596 Medline

78. V. Pons, P. Lévesque, M. M. Plante, S. Rivest, Conditional genetic deletion of CSF1 receptor in microglia ameliorates the physiopathology of Alzheimer’s disease. *Alzheimers Res. Ther.* **13**, 8 (2021). doi:10.1186/s13195-020-00747-7 Medline

79. X. F. Zhao, M. M. Alam, Y. Liao, T. Huang, R. Mathur, X. Zhu, Y. Huang, Targeting microglia using Cx3cr1-Cre lines: Revisiting the specificity. *eNeuro* **6**, ENEURO.0114-19.2019 (2019). doi:10.1523/ENEURO.0114-19.2019 Medline

80. M. Quiñones, O. Al-Massadi, C. Folgueira, S. Bremser, R. Gallego, L. Torres-Leal, R. Haddad-Tóvolli, C. García-Caceres, R. Hernandez-Bautista, B. Y. H. Lam, D. Beiroa, E. Sanchez-Rebordelo, A. Senra, J. A. Malagon, P. Valerio, M. F. Fondevila, J. Fernø, M. M. Malagon, R. Contreras, P. Pfluger, J. C. Brüning, G. Yeo, M. Tschöp, C. Diéguez, M. López, M. Claret, P. Kloppenburg, G. Sabio, R. Nogueiras, p53 in AgRP neurons is required for protection against diet-induced obesity via JNK1. *Nat. Commun.* **9**, 3432 (2018). doi:10.1038/s41467-018-05711-6 Medline

81. Y. Aguilera, N. Mellado-Damas, L. Olmedo-Moreno, V. López, C. Panadero-Morón, M. Benito, H. Guerrero-Cázares, C. Márquez-Vega, A. Martín-Montalvo, V. Capilla-González, Preclinical Safety Evaluation of Intranasally Delivered Human Mesenchymal

Stem Cells in Juvenile Mice. *Cancers* **13**, 1–18 (2021). doi:10.3390/cancers13051169 Medline

82. L. K. Pantier, J. Li, C. A. Christian, Estrous Cycle Monitoring in Mice with Rapid Data Visualization and Analysis. *Bio Protoc.* **9**, e3354 (2019). doi:10.21769/BioProtoc.3354 Medline

83. T. Endo, E. Freinkman, D. G. de Rooij, D. C. Page, Periodic production of retinoic acid by meiotic and somatic cells coordinates four transitions in mouse spermatogenesis. *Proc. Natl. Acad. Sci. U.S.A.* **114**, E10132–E10141 (2017). doi:10.1073/pnas.1710837114 Medline

84. F. Gaytan, C. Morales, S. Leon, V. Heras, A. Barroso, M. S. Avendaño, M. J. Vazquez, J. M. Castellano, J. Roa, M. Tena-Sempere, Development and validation of a method for precise dating of female puberty in laboratory rodents: The puberty ovarian maturation score (Pub-Score). *Sci. Rep.* **7**, 46381 (2017). doi:10.1038/srep46381 Medline

85. C. Brisken, S. Park, T. Vass, J. P. Lydon, B. W. O'Malley, R. A. Weinberg, A paracrine role for the epithelial progesterone receptor in mammary gland development. *Proc. Natl. Acad. Sci. U.S.A.* **95**, 5076–5081 (1998). doi:10.1073/pnas.95.9.5076 Medline

86. P. Pellegrini, A. Cordero, M. I. Gallego, W. C. Dougall, P. Muñoz, M. A. Pujana, E. Gonzalez-Suarez, D. C. William, P. Muñoz, M. Purificación, M. A. Pujana, P. M. Angel, E. Gonzalez-Suarez, G.-S. Eva, Constitutive activation of RANK disrupts mammary cell fate leading to tumorigenesis. *Stem Cells* **31**, 1954–1965 (2013). doi:10.1002/stem.1454 Medline

87. E. Torres, I. Velasco, D. Franssen, V. Heras, F. Gaytan, S. Leon, V. M. Navarro, R. Pineda, M. L. Candenas, A. Romero-Ruiz, M. Tena-Sempere, Congenital ablation of *Tacr2* reveals overlapping and redundant roles of NK2R signaling in the control of reproductive axis. *Am. J. Physiol. Endocrinol. Metab.* **320**, E496–E511 (2021). doi:10.1152/ajpendo.00346.2020 Medline

88. R. Porteous, P. Haden, E. C. R. Hackwell, A. Singline, M. K. Herde, R. Desai, D. J. Handelsman, D. R. Grattan, A. E. Herbison, Reformulation of PULSAR for Analysis of Pulsatile LH Secretion and a Revised Model of Estrogen-Negative Feedback in Mice. *Endocrinology* **162**, bqab165 (2021). doi:10.1210/endocr/bqab165 Medline

89. F. J. Steyn, Y. Wan, J. Clarkson, J. D. Veldhuis, A. E. Herbison, C. Chen, Development of a methodology for and assessment of pulsatile luteinizing hormone secretion in juvenile and adult male mice. *Endocrinology* **154**, 4939–4945 (2013). doi:10.1210/en.2013-1502 Medline

90. B. Mora-Romero, N. Capelo-Carrasco, J. J. Pérez-Moreno, M. I. Alvarez-Vergara, L. Trujillo-Estrada, C. Romero-Molina, E. Martinez-Marquez, N. Morano-Catalan, M. Vizuete, J. Lopez-Barneo, J. L. Nieto-Gonzalez, P. Garcia-Junco-Clemente, J. Vitorica, A. Gutierrez, D. Macias, A. E. Rosales-Nieves, A. Pascual, Microglia mitochondrial complex I deficiency during development induces glial dysfunction and early lethality. *Nat. Metab.* **6**, 1479–1491 (2024). doi:10.1038/s42255-024-01081-0 Medline

91. M. Manfredi-Lozano, V. Leysen, M. Adamo, I. Paiva, R. Rovera, J. M. Pignat, F. E. Timzoura, M. Candlish, S. Eddarkaoui, S. A. Malone, M. S. B. Silva, S. Trova, M. Imbernon, L. Decoster, L. Cotellessa, M. Tena-Sempere, M. Claret, A. Paoloni-

Giacobino, D. Plassard, E. Paccou, N. Vionnet, J. Acierno, A. M. Maceski, A. Lutti, F. Pfrieger, S. Rasika, F. Santoni, U. Boehm, P. Ciofi, L. Buée, N. Haddjeri, A. L. Boutillier, J. Kuhle, A. Messina, B. Draganski, P. Giacobini, N. Pitteloud, V. Prevot, GnRH replacement rescues cognition in Down syndrome. *Science* **377**, eabq4515 (2022). doi:10.1126/science.abq4515 Medline

92. A. Overgaard, F. Ruiz-Pino, J. M. Castellano, M. Tena-Sempere, J. D. Mikkelsen, Disparate changes in kisspeptin and neurokinin B expression in the arcuate nucleus after sex steroid manipulation reveal differential regulation of the two KNDy peptides in rats. *Endocrinology* **155**, 3945–3955 (2014). doi:10.1210/en.2014-1200 Medline

93. Z. Tang, B. Kang, C. Li, T. Chen, Z. Zhang, GEPIA2: An enhanced web server for large-scale expression profiling and interactive analysis. *Nucleic Acids Res.* **47** (W1), W556–W560 (2019). doi:10.1093/nar/gkz430 Medline

94. L. García-Jimeno, C. Fustero-Torre, M. J. Jiménez-Santos, G. Gómez-López, T. Di Domenico, F. Al-Shahrour, bollito: A flexible pipeline for comprehensive single-cell RNA-seq analyses. *Bioinformatics* **38**, 1155–1156 (2022). doi:10.1093/bioinformatics/btab758 Medline

95. A. Frankish, M. Diekhans, I. Jungreis, J. Lagarde, J. E. Loveland, J. M. Mudge, C. Sisu, J. C. Wright, J. Armstrong, I. Barnes, A. Berry, A. Bignell, C. Boix, S. Carbonell Sala, F. Cunningham, T. Di Domenico, S. Donaldson, I. T. Fiddes, C. García Girón, J. M. Gonzalez, T. Grego, M. Hardy, T. Hourlier, K. L. Howe, T. Hunt, O. G. Izuogu, R. Johnson, F. J. Martin, L. Martínez, S. Mohanan, P. Muir, F. C. P. Navarro, A. Parker, B. Pei, F. Pozo, F. C. Riera, M. Ruffier, B. M. Schmitt, E. Stapleton, M. M. Suner, I. Sycheva, B. Uszczynska-Ratajczak, M. Y. Wolf, J. Xu, Y. T. Yang, A. Yates, D. Zerbino, Y. Zhang, J. S. Choudhary, M. Gerstein, R. Guigó, T. J. P. Hubbard, M. Kellis, B. Paten, M. L. Tress, P. Flicek, GENCODE 2021. *Nucleic Acids Res.* **49** (D1), D916–D923 (2021). doi:10.1093/nar/gkaa1087 Medline

96. A. Dobin, C. A. Davis, F. Schlesinger, J. Drenkow, C. Zaleski, S. Jha, P. Batut, M. Chaisson, T. R. Gingeras, STAR: Ultrafast universal RNA-seq aligner. *Bioinformatics* **29**, 15–21 (2013). doi:10.1093/bioinformatics/bts635 Medline

97. T. Stuart, A. Butler, P. Hoffman, C. Hafemeister, E. Papalexi, W. M. Mauck 3rd, Y. Hao, M. Stoeckius, P. Smibert, R. Satija, Comprehensive Integration of Single-Cell Data. *Cell* **177**, 1888–1902.e21 (2019). doi:10.1016/j.cell.2019.05.031 Medline

98. D. Aran, A. P. Looney, L. Liu, E. Wu, V. Fong, A. Hsu, S. Chak, R. P. Naikawadi, P. J. Wolters, A. R. Abate, A. J. Butte, M. Bhattacharya, Reference-based analysis of lung single-cell sequencing reveals a transitional profibrotic macrophage. *Nat. Immunol.* **20**, 163–172 (2019). doi:10.1038/s41590-018-0276-y Medline

99. A. Subramanian, P. Tamayo, V. K. Mootha, S. Mukherjee, B. L. Ebert, M. A. Gillette, A. Paulovich, S. L. Pomeroy, T. R. Golub, E. S. Lander, J. P. Mesirov, Gene set enrichment analysis: A knowledge-based approach for interpreting genome-wide expression profiles. *Proc. Natl. Acad. Sci. U.S.A.* **102**, 15545–15550 (2005). doi:10.1073/pnas.0506580102 Medline
